# Synthesis of Oxazoles Containing CF_3_-Substituted Alcohol Unit via Tandem Cycloisomerization/Hydroxyalkylation from *N*-Propargylamides with Trifluoropyruvates

**DOI:** 10.3390/molecules29245848

**Published:** 2024-12-11

**Authors:** Juan-Juan Gao, Long-Hui Wu, Shu-Qin Yu, Xue Zhu, Yu Zeng, Kai Yang, Zhao-Yang Wang

**Affiliations:** 1 Jiangxi Province Key Laboratory of Pharmacology of Traditional Chinese Medicine, College of Pharmacy, Gannan Medical University, Ganzhou 341000, China; gaoya0758@163.com (J.-J.G.); 13970997583@163.com (L.-H.W.); yu20241211@163.com (S.-Q.Y.); 14760556396@139.com (X.Z.); 2 School of Chemistry, South China Normal University, Guangzhou Key Laboratory of Analytical Chemistry for Biomedicine, GDMPA Key Laboratory for Process Control and Quality Evaluation of Chiral Pharmaceuticals, Key Laboratory of Theoretical Chemistry of Environment, Ministry of Education, Guangzhou 510006, China; 2023022534@m.scnu.edu.cn

**Keywords:** oxazole, trifluoromethyl carbinol unit, tandem reaction, cycloisomerization, hydroxyalkylation, *N*-propargylamide

## Abstract

Oxazoles are important five-membered heterocycles that contain both nitrogen and oxygen atoms. Due to their wide range of biological activities, many oxazoles demonstrate potential for extensive application in various fields, including medicinal chemistry. Trifluoromethyl carbinol, an important pharmacophore, contains both trifluoromethyl and hydroxyl groups and is common in molecules with important biological activities. Constructing oxazoles that contain a trifluoromethyl carbinol unit is undoubtedly important and valuable for expanding the chemical space in drug discovery. In this study, a simple and efficient method was developed for the synthesis of oxazoles containing a CF_3_-substituted alcohol unit via the tandem cycloisomerization/hydroxyalkylation of *N*-propargylamides with trifluoropyruvates through a rational Lewis acid catalytic mechanism. This Zn(OTf)_2_-catalyzed synthetic protocol is operationally simple and provides a series of oxazoles in moderate to good yields. The protocol demonstrates broad substrate scope, high functional group tolerance, and high atom economy and can achieve gram-level reactions, indicating the strong possibility of its practical application.

## 1. Introduction

**Figure 1:**
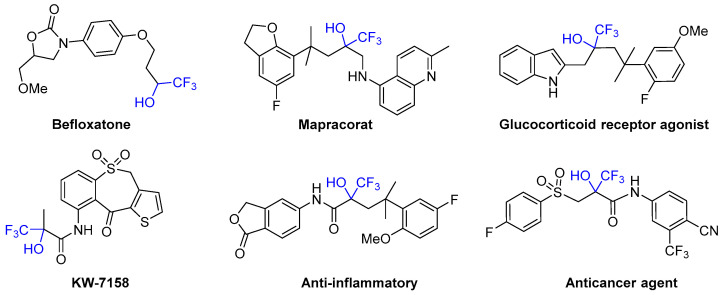
Examples of bioactive molecules containing trifluoromethyl carbinol.

Due to their unique pharmacokinetic properties, such as bioavailability, binding selectivity, metabolic stability, and cell membrane permeability, fluorine-containing groups have been widely used in drug design in recent years [1]. In particular, trifluoromethyl carbinols containing both trifluoromethyl and hydroxyl groups are a class of molecules that often exhibit significant biological activities [2,3]; some typical examples can be seen in Figure 1 [4,5,6]. Therefore, constructing molecules that contain trifluoromethyl carbinol is undoubtedly important and valuable for expanding the chemical space in drug discovery [7,8].

**Figure 2:**
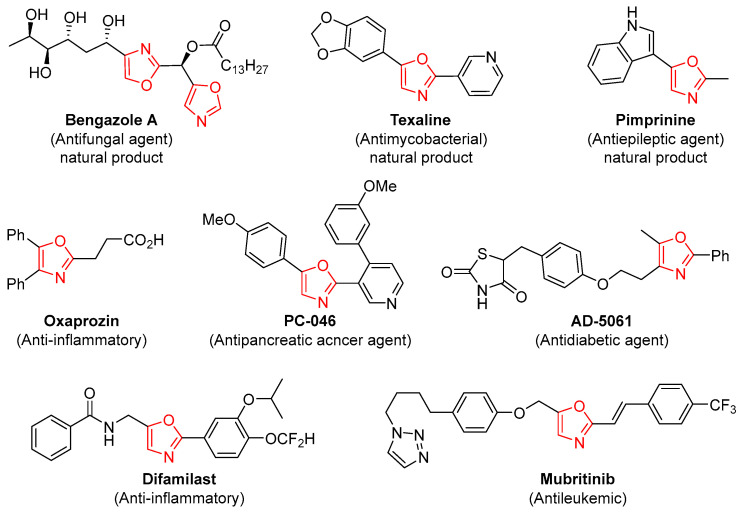
Examples of natural products and biologically active molecules containing an oxazole unit.

The oxazole moiety is one of the most common heterocycles in structurally diverse natural products and pharmaceutically active ingredients [9,10]. As an important class of heterocycles in medicinal chemistry, oxazole-containing compounds typically exhibit significant biological activities, such as anticancer [11], antidiabetic [12], antiepileptic [13], anti-inflammatory [14], antimalarial [15], antimicrobial [16], antitubercular [17], and antiviral activities [18,19] (Figure 2). Moreover, oxazole derivatives have important applications as intermediates and metal catalysis ligands in organic synthesis [20]. Additionally, oxazole compounds have important structural motif applications in fluorescent probes [21] and agricultural chemicals [22].

Therefore, the synthesis of oxazole derivatives containing multiple substituents has received widespread attention [23,24]. It should be noted that although methods of synthesizing structurally diverse oxazole derivatives have been continuously developed and successfully applied to the synthesis of various bioactive molecules [25,26], efficient and green synthesis methods for oxazole derivatives containing special functionalized components still require further research and development [27].

**Scheme 1:**
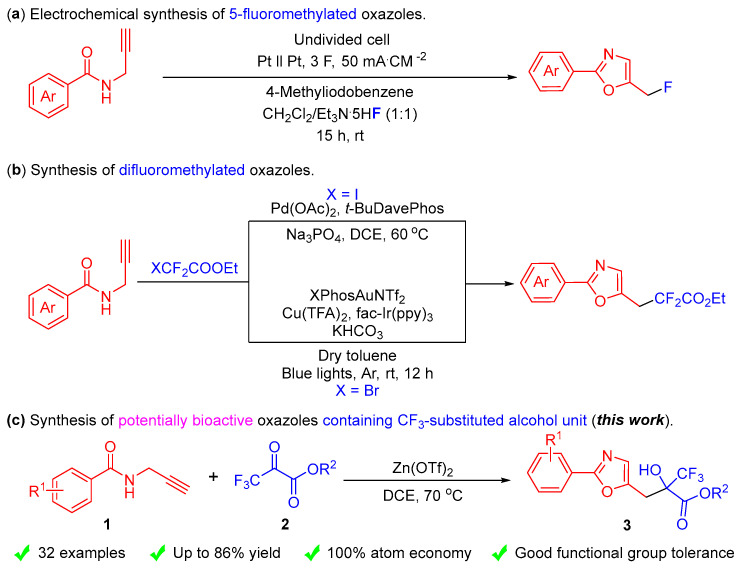
Typical examples of synthesis methods using tandem reactions to obtain fluorinated oxazoles.

Among the different synthesis methods, those involving fluorinated oxazole derivatives are constantly being investigated [28,29,30]. For example, Doherty’s group [31] synthesized oxazolines through the 5-exo-dig cycloisomerization of *N*-propargylamides catalyzed using gold complexes, and then, via the carbonyl-ene reaction of oxazolines with ethyl trifluoropyruvate, they synthesized the corresponding hydroxytrifluoroalkylated oxazoles under the catalysis of [PdCl_2_{(S)-BINAP}]. However, a drawback of this method is that, in addition to requiring two steps, it uses Au and Pd catalysts, which are costly, thus necessitating the further development of novel methods involving the tandem reaction shown in Scheme 1.

In fact, it has been demonstrated that the use of a tandem reaction involving the cyclization of *N*-propargylamides with various fluorinating reagents is a common, efficient strategy for synthesizing 5-substituted oxazole derivatives (including fluorinated oxazoles) [32,33]. For example, Waldvogel’s group utilized electrochemistry to synthesize 5-fluoromethyl-2-oxazoles starting with easily available *N*-propargylamides (Scheme 1a) [34].

Notably, Liang’s group [35] and Li’s group [36] successfully used the palladium-catalyzed and photo-mediated cascade reactions of ethyl halodifluoroacetates, respectively, to synthesize 5-difluoromethylated oxazole compounds from *N*-propargylamides (Scheme 1b).

Although the abovementioned methods are useful for constructing fluorinated oxazoles, it is important to develop other green, simple, and effective routes, including the cascade reaction of trifluoropyruvates [7,8], for the synthesis of more oxazole compounds with diverse fluorinated structures, particularly bioactive oxazoles containing a trifluoromethyl carbinol unit, from low-cost fluorinating reagents.

Therefore, due to the potential applications of fluorinated heterocycles and based on our interest in methods of synthesizing heterocycles [37,38,39], particularly the synthesis and derivation of fluorinated heterocycles [40,41], we herein disclose a facile, efficient, and Zn(OTf)_2_-catalyzed method for the synthesis of oxazoles containing CF_3_-substituted alcohols via tandem cycloisomerization/hydroxyalkylation using *N*-propargylamides with trifluoropyruvates (Scheme 1c).

This synthetic protocol has the following advantages: simple operation, 100% atom economy, lack of oxidants, good functional group tolerance, and applicability to a wide range of substrates. Critically, this newly developed strategy will contribute to the further design and rapid synthesis of fluorinated oxazole derivatives with potential biological activities.

## 2. Results and Discussion

### 2.1. Optimization of Reaction Conditions

Initially, the reaction of *N*-(prop-2-yn-1-yl)benzamide **1a** (0.3 mmol) and ethyl trifluoropyruvate **2a** (0.36 mmol) was selected to screen proper conditions for the expected reaction. The reaction conditions were optimized, and the reaction parameters were established, which are listed in Table 1.

Obviously, the desired product, **3a**, cannot be obtained via a reaction in dichloroethane (DCE) at 70 °C for 12 h in the absence of any catalysts (Table 1, entry 1). Fortunately, when 20 mol% FeCl_3_, a Lewis acid, was added as a catalyst, compound **3a** could be successfully obtained with a yield of 56% (entry 2). Other Lewis acids, such as InCl_3_, Y(OTf)_3_, Sc(OTf)_3_, In(OTf)_3_, Cu(OTf)_2_, and Zn(OTf)_2_, were also examined (entries 3–8). The results show that Zn(OTf)_2_ is the most efficient and can result in 83% yield (entry 8).

To remove the effect of TfOH liberated from Zn(OTf)_2_, we conducted the experiment using trifluoromethanesulfonic acid (TfOH) as a catalyst. The research results indicate that **3a** (entry 9) could not be achieved using only TfOH as a catalyst. The amount of Zn(OTf)_2_ was further optimized (entries 8, 10–12). The results reveal that using 15 mol% Zn(OTf)_2_ is the most effective strategy (entry 11).

The solvents used were further optimized. Compared to other solvents (such as toluene, 1,4-dioxane, acetonitrile, chloroform, THF, and DMF), DCE provided the best result (entry 11 vs. entries 13–18). Upon examining the reaction temperatures, it was found that 70 °C was the optimum temperature (entry 11 vs. entries 19, 20). In addition, 12 h was the most suitable reaction time (entry 11 vs. entries 21, 22). Thus, the optimized reaction conditions were identified as **1a** (0.3 mmol), **2a** (0.36 mmol), the use of 15 mol% Zn(OTf)_2_ as a catalyst, and the use of 1.5 mL of DCE as the solvent at 70 °C for 12 h.

### 2.2. Scope of Substrates

With the optimized conditions in hand, we explored the generality of the developed method using a variety of *N*-propargylamides (Table 2).

Representative arylamide substrates with a hydrocarbyl group (e.g., methyl, ethyl, methoxy, chloromethyl, and phenyl), as well as a halogen group (e.g., fluoro, chloro, and bromo) in the para position, all worked well with **2a** to afford the desired trifluoroalkylated oxazoles (**3b**–**3i**) in yields ranging from 73% to 83%. Moreover, arylamide substrates with electron-withdrawing groups in the para position, e.g., cyano, methyl ester group (methoxyformyl), and trifluoromethyl, were also suitable for this conversion, resulting in **3j**–**3l** in 68–73% yields.

This conversion was also applicable to substituted arylamide substrates in the ortho or meta positions, producing the corresponding products in moderate yields (72% for **3m**, and 70% for **3n**). Even the use of disubstituted benzamides, such as 3,5-dimethylbenzamide (**1o**) and 3,5-dichlorobenzamide (**1p**), could result in the desired products **3o** and **3p**, with yields of 78% and 75%, respectively.

As expected, both 1-naphthamide (**1q**) and 2-naphthamide (**1r**) substrates exhibited good activity in this conversion, smoothly furnishing the corresponding CF_3_-substituted tertiary carbinols **3q** and **3r** in 70% and 73% yields, respectively. Some typical heteroarylamide substrates **1**, e.g., 2-chloronicotinamide (**1s**), furan-2-carboxamide (**1t**), thiophene-2-carboxamide (**1u**), and 5-chlorothiophene-2-carboxamide (**1v**), could also be smoothly converted, affording **3s**–**3v** in 56–68% yields.

2-Phenyl-*N*-(prop-2-yn-1-yl)acetamide (**1w**), a type of aliphatic amide, was also suitable for use in the protocol, producing a satisfactory result (**3w**). In addition, the use of cinnamamide (**1x**), a specially functionalized amide, resulted in a normal reaction, producing the corresponding trifluoroalkylated oxazole **3x** with a yield of 51%. Captivatingly, when using *N*^1^,*N*^4^-di(prop-2-yn-1-yl)terephthalamide (**1y**) as the starting material, **3y** was obtained under standard conditions with a yield of 48%.

More importantly, considering the potential applications of these trifluoroalkylated oxazole compounds in organic synthesis and biomedicine [4,42], gram-scale synthesis was carried out to demonstrate the practicability of this method. By reacting 4.5 mmol (0.716 g) of **1a** with **2a** (0.918 g) under standard conditions, **3a** could be produced with a yield of 80% (1.183 g).

Next, the substrate scope of different trifluoroacetyl compounds **2** was further evaluated. As shown in Table 3, *N*-(prop-2-yn-1-yl)benzamide **1a** could also react smoothly with methyl trifluoropyruvate **2b** instead of ethyl trifluoropyruvate **2a** under the same conditions to obtain the desired product, **3z**, in good yield (81%).

Interestingly, excellent substrate applicability was also observed when 1,1,1,3,3,3-hexafluoropropan-2-one (**2c**) was used to replace the substrate trifluoropyruvate **2a** or **2b**. For example, *N*-(prop-2-yn-1-yl)benzamide (**1a**) reacted well with **2c**, producing **3aa** with an 86% yield. Various arylamide substrates **1** with either electron-withdrawing (e.g., F, CF_3_, and CN) or electron-donating (e.g., Me) groups all worked well with **2c** to afford the desired oxazoles **3ab**–**3ae** in moderate to good yields (75–80%).

As expected, 5-chlorothiophene-2-carboxamide (**1v**), as a type of heteroarylamide substrate **1**, reacted smoothly with **2c** to produce **3af** with a yield of 56%. Unfortunately, when using 2,2,2-trifluoro-1-phenylethan-1-one (**2d**) and 1,1,1-trifluoropropan-2-one (**2e**) under standard conditions, it was difficult to obtain the corresponding products **3ag** and **3ah**, respectively. This may be due to greater spatial hindrance (for **2d**) or less reactivity (for **2e**) [43,44].

Moreover, the NMR (^1^H, ^13^C, and ^19^F) and HRMS data of products **3a**–**3af** are in good agreement with the anticipated structures (for more details, see the Supplementary Materials). Thus, we successfully characterized the structures of serial compounds **3a**–**3af**.

### 2.3. The Control Experiments and Plausible Reaction Mechanism

**Scheme 2:**
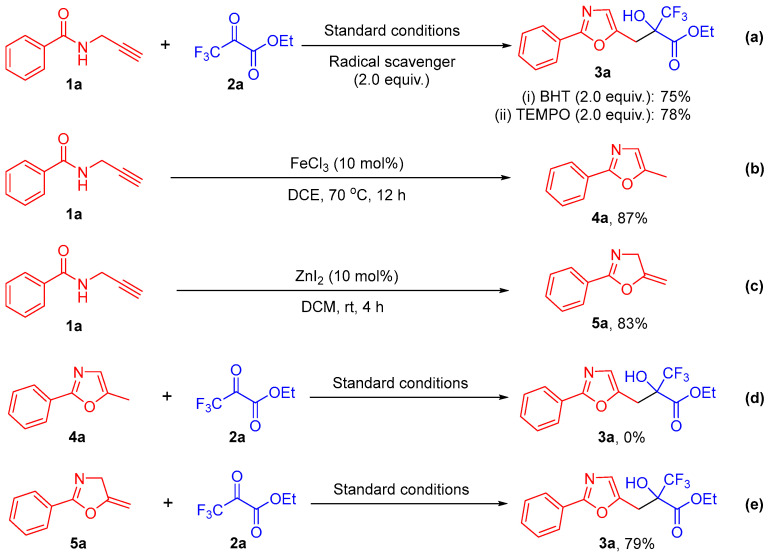
Control experiments: (**a**) Explore whether there is a free radical pathway. (**b**) Synthesis of **4a** catalyzed by FeCl_3_. (**c**) Synthesis of **5a** catalyzed by ZnI_2_. (**d**) Explore whether **4a** is an intermediate in the reaction. (**e**) Explore whether **5a** is an intermediate in the reaction.

In order to further explore the possible reaction mechanism, we conducted several relevant control experiments (Scheme 2).

First, adding two equivalents of the radical scavenger butylated hydroxytoluene (BHT) or 2,2,6,6-tetrameth yl-1-piperidinoxy (TEMPO) [45,46] into the reaction system, the corresponding compound **3a** was obtained at 75% and 78% yields, respectively (Scheme 2a). This result indicates that this reaction may not involve a radical pathway.

Second, small amounts of oxazolines and oxazoles were detected in the reaction. We therefore referred to the relevant literature [47,48] and synthesized oxazole **4a** (Scheme 2b) and oxazoline **5a** (Scheme 2c), using FeCl_3_ and ZnI_2_ as catalysts, respectively. When oxazole **4a** was reacted with **2a** under standard conditions, **3a** was not produced (Scheme 2d), indicating that the reaction process might not involve the formation of **4a**. Notably, **4a** was simply a byproduct of the reaction, while oxazoline **5a** could further react with **2a** to afford the final product **3a** in an isolated yield of 79% (Scheme 2e). This implies that oxazoline **5a** is a key intermediate in this process.

**Scheme 3:**
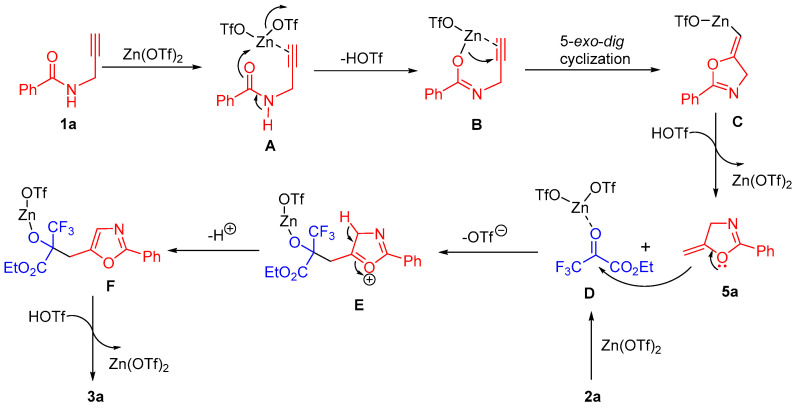
Proposed mechanisms.

Therefore, based on previously reported studies [35,47,48,49,50,51,52,53,54,55,56,57] and the aforementioned control experiments, a possible reaction pathway is proposed using the reaction of **1a** and **2a** as an example, as shown in Scheme 3.

First, as a Lewis acid, Zn(OTf)_2_ can activate the triple bond of **1a** to enhance the electrophilicity of alkyne **A**, and the intermediate **B** is formed through the loss of HOTf from **A** [49,50,51,52]. Then, the regioselective intramolecular 5-exo-dig cyclization of **B** affords the intermediate **C** [53,54]. Hydrolysis with HOTf (which is generated in situ, as mentioned above) occurs, resulting in the oxazoline intermediate **5a** [49,51].

On the other hand, Zn(OTf)_2_ can separately coordinate with the carbonyl group of **2a** to form an electrophilic intermediate **D** [55,57]. Subsequently, the carbonyl-ene reaction between **5a** and **D** gives **F** via the intermediate **E** with the loss of a proton [56,57].

In the end, with the hydrolysis via HOTf generated in situ, the final product **3a** is produced from the intermediate **F** with the regeneration of Zn(OTf)_2_ [52], completing the catalyst cycle.

## 3. Materials and Methods

### 3.1. General Information

The melting point (m.p.) was measured using a Büchi Melting Point B-545 instrument (Büchi, Flawil, Switzerland) without correction. ^1^H, ^13^C, and ^19^F NMR spectra were recorded on a BRUKER DRX-400 spectrometer (Bruker, Ettlingen, Germany) (^1^H NMR at 400 MHz, ^13^C NMR at 100 MHz, and ^19^F NMR at 376 MHz), using tetramethylsilane (TMS) as an internal standard. High-resolution mass spectra (HRMS) were recorded on an LCMS-IT-TOF mass spectrometer (Waters Corporation, Milford, MA, USA). Thin-layer chromatography (TLC) was performed on commercially prepared silica gel plates (GF254). Visualization of TLC was performed with a 254 nm UV lamp. The crude products were purified via column chromatography using 100–200-mesh silica gel.

All chemicals and organic solvents were used as received from commercial sources without further purification, and the substrates *N*-propargylamides **1** were synthesized from prop-2-yn-1-amine and various acyl chlorides as reported in [49,54].

### 3.2. Experimental Procedure for Compounds ***1a***–***1y***

As shown in Table 4, compounds **1a**–**1y** were synthesized according to the reported procedure [49,54].

Propargylamine (10 mmol) was dissolved in 20 mL of dry dichloromethane (DCM), and 12 mmol Et_3_N was added. The reaction mixture was cooled to 0 °C, and 10 mmol of acid chloride was added. The mixture was stirred for 15 min at 0 °C and then for 2–4 h at room temperature. After the reaction was completed, water was added, and the mixture was then extracted with DCM. The combined organic layer was dried over MgSO_4_, and the solvent was removed in vacuo. The crude *N*-propargylamides **1a**–**1y**, as substrates, were further purified via column chromatography or by recrystallization with 80–96% yields.

### 3.3. Experimental Procedure for Compounds ***3a***–***3af***

*N*-propargylamides **1** (0.3 mmol, 1.0 equiv.), trifluoroacetyl compound **2** (0.36 mmol, 1.2 equiv.), and Zn(OTf)_2_ (0.045 mmol, 15 mol%) were dissolved in 1.5 mL of 1,2-dichloroethane (DCE). The reaction mixture was stirred for 12 h at 70 °C. After the reaction was completed, water was added, and the mixture was extracted with ethyl acetate (3 × 15 mL). The combined organic layer was dried over MgSO_4_, and the solvent was removed under reduced pressure. The crude products **3a**–**3af** were further purified via column chromatography.

### 3.4. Experimental Procedure for Compounds ***4a***

A mixture of *N*-(prop-2-yn-1-yl)benzamide **1a** (0.3 mmol, 1.0 equiv.) and FeCl_3_ (0.03 mmol, 10 mol%) was dissolved in 1.5 mL of DCE. The mixture was stirred for 12 h at 70 °C. After the reaction was completed, water was added, and the mixture was extracted with ethyl acetate (3 × 15 mL). The combined organic layer was dried over MgSO_4_, and the solvent was removed under reduced pressure. The crude product **4a** was further purified via column chromatography.

### 3.5. Experimental Procedure for Compounds ***5a***

A mixture of *N*-(prop-2-yn-1-yl)benzamide **1a** (0.3 mmol, 1.0 equiv.) and ZnI_2_ (0.03 mmol, 10 mol%) was dissolved in 1.5 mL of dichloromethane (DCM). The mixture was stirred for 4 h at room temperature. After the reaction was completed, water was added, and the mixture was extracted with ethyl acetate (3 × 15 mL). The combined organic layer was dried over MgSO_4_, and the solvent was removed under reduced pressure. The crude product **5a** was further purified via column chromatography.

### 3.6. Characterization Data for All Products ***3a***–***3af***, ***4a***, and ***5a***

The structures of the target products have been well characterized by ^1^H NMR, ^13^C NMR, ^19^F NMR, and HRMS. The corresponding data are summarized in the following.

(1) Ethyl 3,3,3-trifluoro-2-hydroxy-2-(2-phenyloxazol-5-yl)methylpropanoate (**3a**): colorless oil, 84% yield; ^1^H NMR (400 MHz, CDCl_3_), *δ*: 7.97–7.95 (m, 2H, ArH), 7.45–7.43 (m, 3H, ArH), 7.01 (s, 1H, ArH), 4.43–4.35 (m, 2H, OCH_2_), 4.24 (s, 1H, OH), 3.54 (d, *J* = 15.2 Hz, 1H, CH_2_-a), 3.35 (d, *J* = 15.2 Hz, 1H, CH_2_-b), 1.34 (t, *J* = 7.2 Hz, 3H, CH_3_) ppm; ^19^F NMR (376 MHz, CDCl_3_), *δ*: −78.52 ppm; ^13^C NMR (100 MHz, CDCl_3_), *δ*: 168.5, 161.8, 144.9, 130.4, 128.8, 127.4, 127.2, 126.2, 123.1 (q, *J* = 285.0 Hz), 76.6 (q, *J* = 29.1 Hz), 64,2, 29.1, 14.0 ppm; ESI-HRMS, *m*/*z*: Calcd for C_15_H_15_F_3_NO_4_^+^ [M + H]^+^: 330.0948, found: 330.0943.
(2) Ethyl 3,3,3-trifluoro-2-hydroxy-2-(2-(*p*-tolyl)oxazol-5-yl)methylpropanoate (**3b**): colorless oil, 83% yield; ^1^H NMR (400 MHz, CDCl_3_), *δ*: 7.84 (d, *J* = 8.4 Hz, 2H, ArH), 7.24 (d, *J* = 8.4 Hz, 2H, ArH), 6.99 (s, 1H, ArH), 4.42–4.34 (m, 2H, OCH_2_), 4.22 (br, 1H, OH), 3.53 (d, *J* = 14.8 Hz, 1H, CH_2_-a), 3.34 (d, *J* = 14.8 Hz, 1H, CH_2_-b), 1.33 (t, *J* = 7.6 Hz, 3H, CH_3_) ppm; ^19^F NMR (376 MHz, CDCl_3_), *δ*: −78.53 ppm; ^13^C NMR (100 MHz, CDCl_3_), *δ*: 168.5, 162.0, 144.5, 140.7, 129.5, 127.3, 126.1, 125.9 (q, *J* = 285.2 Hz), 124.5, 76.6 (q, *J* = 29.1 Hz), 64,2, 29.1, 21.5, 13.9 ppm; ESI-HRMS, *m*/*z*: Calcd for C_16_H_17_F_3_NO_4_^+^ [M + H]^+^: 344.1104, found: 344.1120.
(3) Ethyl 2-(2-(4-ethylphenyl)oxazol-5-yl)methyl-3,3,3-trifluoro-2-hydroxypropanoate (**3c**): colorless oil, 76% yield; ^1^H NMR (400 MHz, CDCl_3_), *δ*: 7.87 (d, *J* = 7.6 Hz, 2H, ArH), 7.26 (d, *J* = 7.6 Hz, 2H, ArH), 6.98 (s, 1H, ArH), 4.41 (br, 1H, OH), 4.38–4.33 (m, 2H, OCH_2_), 3.52 (d, *J* = 14.8 Hz, 1H, CH_2_-a), 3.33 (d, *J* = 14.8 Hz, 1H, CH_2_-b), 2.68 (q, *J* = 7.6 Hz, 3H, CH_2_), 1.33 (t, *J* = 7.6 Hz, 3H, CH_3_), 1.25 (t, *J* = 7.6 Hz, 3H, CH_3_) ppm; ^19^F NMR (376 MHz, CDCl_3_), *δ*: −78.51 ppm; ^13^C NMR (100 MHz, CDCl_3_), *δ*: 167.1, 160.9, 146.0, 143.5, 127.3, 126.2, 125.2, 123.7, 122.1 (q, *J* = 285.0 Hz), 75.6 (q, *J* = 29.2 Hz), 63,1, 28.1, 27.8, 14.3, 12.9 ppm; ESI-HRMS, *m*/*z*: Calcd for C_17_H_19_F_3_NO_4_^+^ [M + H]^+^: 358.1261, found: 358.1269.
(4) Ethyl 3,3,3-trifluoro-2-hydroxy-2-(2-(4-methoxyphenyl)oxazol-5-yl)methylpropano- ate (**3d**): white solid, 81% yield, m.p. 73–75 °C; ^1^H NMR (400 MHz, CDCl_3_), *δ*: 7.89 (d, *J* = 8.4 Hz, 2H, ArH), 6.97 (s, 1H, ArH), 6.95 (d, *J* = 8.4 Hz, 2H, ArH), 4.39–4.23 (m, 2H, OCH_2_), 3.85 (br, 1H, OH), 3.51 (d, *J* = 15.2 Hz, 1H, CH_2_-a), 3.32 (d, *J* = 15.2 Hz, 1H, CH_2_-b), 1.33 (t, *J* = 7.6 Hz, 3H, CH_3_) ppm; ^19^F NMR (376 MHz, CDCl_3_), *δ*: −78.52 ppm; ^13^C NMR (100 MHz, CDCl_3_), *δ*: 168.5, 167.4, 161.5, 144.3, 128.0, 126.7, 123.1 (q, *J* = 285.2 Hz), 119.6, 114.3, 76.6 (q, *J* = 29.1 Hz), 64,3, 55.4, 29.0, 13.9 ppm; ESI-HRMS, *m*/*z*: Calcd for C_16_H_17_F_3_NO_5_^+^ [M + H]^+^: 360.1053, found: 360.1075.
(5) Ethyl 2-(2-(4-(chloromethyl)phenyl)oxazol-5-yl)methyl-3,3,3-trifluoro-2-hydroxy- propanoate (**3e**): colorless oil, 75% yield; ^1^H NMR (400 MHz, CDCl_3_), *δ*: 7.95 (d, *J* = 8.4 Hz, 2H, ArH), 7.47 (d, *J* = 8.4 Hz, 2H, ArH), 7.02 (s, 1H, ArH), 4.61 (s, 1H, ClCH_2_), 4.43–4.32 (m, 2H, OCH_2_), 4.20 (br, 1H, OH), 3.53 (d, *J* = 15.2 Hz, 1H, CH_2_-a), 3.34 (d, *J* = 15.2 Hz, 1H, CH_2_-b), 1.34 (t, *J* = 8.0 Hz, 3H, CH_3_) ppm; ^19^F NMR (376 MHz, CDCl_3_), *δ*: −78.52 ppm; ^13^C NMR (100 MHz, CDCl_3_), *δ*: 168.5, 161.2, 145.2, 139.7, 129.1, 127.4, 127.1, 126.5, 123.0 (q, *J* = 285.0 Hz), 76.7 (q, *J* = 29.2 Hz), 64.3, 45.6, 29.1, 14.0 ppm; ESI-HRMS, *m*/*z*: Calcd for C_16_H_16_ClF_3_NO_4_^+^ [M + H]^+^: 378.0714, found: 378.0714.
(6) Ethyl 2-(2-([1,1′-biphenyl]-4-yl)oxazol-5-yl)methyl-3,3,3-trifluoro-2-hydroxypro- panoate (**3f**): white solid, 74% yield, m.p. 107–109 °C; ^1^H NMR (400 MHz, CDCl_3_), *δ*: 8.01 (d, *J* = 8.4 Hz, 2H, ArH), 7.63 (d, *J* = 8.4 Hz, 2H, ArH), 7.58 (d, *J* = 8.0 Hz, 2H, ArH), 7.44–7.40 (m, 2H, ArH), 7.35 (t, *J* = 8.0 Hz, 1H, ArH), 7.03 (s, 1H, ArH), 4.72 (br, 1H, OH), 4.41–4.33 (m, 2H, OCH_2_), 3.54 (d, *J* = 15.6 Hz, 1H, CH_2_-a), 3.35 (d, *J* = 15.6 Hz, 1H, CH_2_-b), 1.32 (t, *J* = 7.6 Hz, 3H, CH_3_) ppm; ^19^F NMR (376 MHz, CDCl_3_), *δ*: −78.41 ppm; ^13^C NMR (100 MHz, CDCl_3_), *δ*: 168.5, 161.6, 145.1, 143.1, 140.1, 128.9, 127.9, 127.5, 127.4, 127.1, 126.7, 126.0, 123.2 (q, *J* = 285.1 Hz), 76.8 (q, *J* = 31.8 Hz), 64.1, 29.2, 14.0 ppm; ESI-HRMS, *m*/*z*: Calcd for C_21_H_19_F_3_NO_4_^+^ [M + H]^+^: 406.1261, found: 406.1254.
(7) Ethyl 3,3,3-trifluoro-2-(2-(4-fluorophenyl)oxazol-5-yl)methyl-2-hydroxypropanoate (**3g**): colorless oil, 80% yield; ^1^H NMR (400 MHz, CDCl_3_), *δ*: 7.96–7.93 (m, 2H, ArH), 7.15–7.10 (m, 2H, ArH), 6.99 (s, 1H, ArH), 4.41–4.33 (m, 2H, OCH_2_), 4.31 (br, 1H, OH), 3.52 (d, *J* = 14.4 Hz, 1H, CH_2_-a), 3.34 (d, *J* = 14.4 Hz, 1H, CH_2_-b), 1.33 (t, *J* = 7.2 Hz, 3H, CH_3_) ppm; ^19^F NMR (376 MHz, CDCl_3_), *δ*: −78.53, −109.32 ppm; ^13^C NMR (100 MHz, CDCl_3_), *δ*: 168.5, 164.1 (d, *J* = 250.2 Hz), 160.9, 145.0, 128.3 (d, *J* = 8.8 Hz), 127.4, 123.6 (d, *J* = 3.2 Hz), 123.1 (q, *J* = 285.2 Hz), 116.0 (d, *J* = 22.8 Hz), 76.6 (q, *J* = 29.1 Hz), 64,2, 29.0, 13.9 ppm; ESI-HRMS, *m*/*z*: Calcd for C_15_H_14_F_4_NO_4_^+^ [M + H]^+^: 348.0853, found: 348.0844.
(8) Ethyl 2-(2-(4-chlorophenyl)oxazol-5-yl)methyl-3,3,3-trifluoro-2-hydroxypropanoate (**3h**): colorless oil, 77% yield; ^1^H NMR (400 MHz, CDCl_3_), *δ*: 7.89 (d, *J* = 8.0 Hz, 2H, ArH), 7.42 (d, *J* = 8.4 Hz, 2H, ArH), 7.01 (s, 1H, ArH), 4.43–4.33 (m, 2H, OCH_2_), 4.19 (br, 1H, OH), 3.53 (d, *J* = 15.2 Hz, 1H, CH_2_-a), 3.35 (d, *J* = 15.2 Hz, 1H, CH_2_-b), 1.34 (t, *J* = 7.6 Hz, 3H, CH_3_) ppm; ^19^F NMR (376 MHz, CDCl_3_), *δ*: −78.53 ppm; ^13^C NMR (100 MHz, CDCl_3_), *δ*: 168.5, 160.9, 145.2, 136.6, 129.2, 127.6, 127.4, 125.7, 123.0 (q, *J* = 285.2 Hz), 76.6 (q, *J* = 31.8 Hz), 64,3, 29.0, 14.0 ppm; ESI-HRMS, *m*/*z*: Calcd for C_15_H_14_ClF_3_NO_4_^+^ [M + H]^+^: 364.0558, found: 364.0578.
(9) Ethyl 2-(2-(4-bromophenyl)oxazol-5-yl)methyl-3,3,3-trifluoro-2-hydroxypropanoate (**3i**): white solid, 75% yield, m.p. 100–102 °C; ^1^H NMR (400 MHz, CDCl_3_), *δ*: 7.81 (d, *J* = 8.4 Hz, 2H, ArH), 7.57 (d, *J* = 8.4 Hz, 2H, ArH), 7.01 (s, 1H, ArH), 4.42–4.33 (m, 2H, OCH_2_), 4.29 (br, 1H, OH), 3.52 (d, *J* = 15.6 Hz, 1H, CH_2_-a), 3.35 (d, *J* = 15.6 Hz, 1H, CH_2_-b), 1.33 (t, *J* = 7.6 Hz, 3H, CH_3_) ppm; ^19^F NMR (376 MHz, CDCl_3_), *δ*: −78.52 ppm; ^13^C NMR (100 MHz, CDCl_3_), *δ*: 167.4, 159.9, 144.3, 131.1, 126.6, 126.5, 125.1, 123.9, 122.0 (q, *J* = 285.0 Hz), 75.6 (q, *J* = 31.8 Hz), 63,2, 28.0, 12.9 ppm; ESI-HRMS, *m*/*z*: Calcd for C_15_H_14_BrF_3_NO_4_^+^ [M + H]^+^: 408.0053, found: 408.0071.
(10) Ethyl 2-(2-(4-cyanophenyl)oxazol-5-yl)methyl-3,3,3-trifluoro-2-hydroxypropanoate (**3j**): colorless oil, 71% yield; ^1^H NMR (400 MHz, CDCl_3_), *δ*: 8.06 (d, *J* = 8.8 Hz, 2H, ArH), 7.74 (d, *J* = 8.8 Hz, 2H, ArH), 7.09 (s, 1H, ArH), 4.44–4.34 (m, 2H, OCH_2_), 4.26 (br, 1H, OH), 3.54 (d, *J* = 15.2 Hz, 1H, CH_2_-a), 3.38 (d, *J* = 15.2 Hz, 1H, CH_2_-b), 1.34 (t, *J* = 7.6 Hz, 3H, CH_3_) ppm; ^19^F NMR (376 MHz, CDCl_3_), *δ*: −78.52 ppm; ^13^C NMR (100 MHz, CDCl_3_), *δ*: 168.4, 159.9, 146.5, 132.7, 130.8, 128.0, 126.6, 123.0 (q, *J* = 285.0 Hz), 118.2, 113.7, 76.7 (q, *J* = 29.2 Hz), 64,4, 29.0, 14.0 ppm; ESI-HRMS, *m*/*z*: Calcd for C_16_H_14_F_3_N_2_O_4_^+^ [M + H]^+^: 355.0900, found: 355.0911.
(11) Methyl 4-(5-(2-(ethoxycarbonyl)-3,3,3-trifluoro-2-hydroxypropyl)oxazol-2-yl)-benzoate (**3k**): colorless oil, 68% yield; ^1^H NMR (400 MHz, CDCl_3_), *δ*: 8.11 (d, *J* = 9.2 Hz, 2H, ArH), 8.03 (d, *J* = 9.2 Hz, 2H, ArH), 7.07 (s, 1H, ArH), 4.44–4.35 (m, 2H, OCH_2_), 4.17 (br, 1H, OH), 3.94 (s, 3H, OCH_3_), 3.55 (d, *J* = 15.2 Hz, 1H, CH_2_-a), 3.38 (d, *J* = 15.2 Hz, 1H, CH_2_-b), 1.35 (t, *J* = 7.2 Hz, 3H, CH_3_) ppm; ^19^F NMR (376 MHz, CDCl_3_), *δ*: −78.54 ppm; ^13^C NMR (100 MHz, CDCl_3_), *δ*: 167.4, 165.4, 159.7, 144.8, 130.5, 129.2, 129.1, 126.8, 125.0, 122.0 (q, *J* = 285.2 Hz), 75.6 (q, *J* = 29.1 Hz), 63.3, 51.3, 28.0, 12.9 ppm; ESI-HRMS, *m*/*z*: Calcd for C_17_H_17_F_3_NO_6_^+^ [M + H]^+^: 388.1002, found: 388.0991.
(12) Ethyl 3,3,3-trifluoro-2-hydroxy-2-(2-(4-(trifluoromethyl)phenyl)oxazol-5-yl)methyl- propanoate (**3l**): white solid, 73% yield, m.p. 95–97 °C; ^1^H NMR (400 MHz, CDCl_3_), *δ*: 8.07 (d, *J* = 8.4 Hz, 2H, ArH), 7.70 (d, *J* = 8.4 Hz, 2H, ArH), 7.06 (s, 1H, ArH), 4.42–4.35 (m, 2H, OCH_2_), 4.21 (br, 1H, OH), 3.55 (d, *J* = 15.2 Hz, 1H, CH_2_-a), 3.38 (d, *J* = 15.2 Hz, 1H, CH_2_-b), 1.35 (t, *J* = 7.6 Hz, 3H, CH_3_) ppm; ^19^F NMR (376 MHz, CDCl_3_), *δ*: −62.97, −78.55 ppm; ^13^C NMR (100 MHz, CDCl_3_), *δ*: 168.5, 160.4, 145.9, 132.0 (q, *J* = 32.3 Hz), 130.3, 127.8, 126.4, 125.9 (q, *J* = 3.8 Hz), 123.8 (q, *J* = 271.4 Hz), 123.0 (q, *J* = 285.6 Hz), 76.6 (q, *J* = 29.2 Hz), 64.3, 29.0, 14.0 ppm; ESI-HRMS, *m*/*z*: Calcd for C_16_H_14_F_6_NO_4_^+^ [M + H]^+^: 398.0822, found: 398.0827.
(13) Ethyl 3,3,3-trifluoro-2-hydroxy-2-(2-(*o*-tolyl)oxazol-5-yl)methylpropanoate (**3m**): colorless oil, 72% yield; ^1^H NMR (400 MHz, CDCl_3_), *δ*: 7.87 (d, *J* = 8.0 Hz, 2H, ArH), 7.35–7.25 (m, 3H, ArH), 7.05 (s, 1H, ArH), 4.44–4.30 (m, 2H, OCH_2_), 4.05 (br, 1H, OH), 3.53 (d, *J* = 14.8 Hz, 1H, CH_2_-a), 3.36 (d, *J* = 14.8 Hz, 1H, CH_2_-b), 2.65 (s, 1H, CH_3_), 1.33 (t, *J* = 7.2 Hz, 3H, CH_3_) ppm; ^19^F NMR (376 MHz, CDCl_3_), *δ*: −78.52 ppm; ^13^C NMR (100 MHz, CDCl_3_), *δ*: 168.6, 162.1, 144.3, 137.4, 131.6, 130.0, 128.6, 127.2, 126.2, 126.0, 123.1 (q, *J* = 285.2 Hz), 76.7 (q, *J* = 29.1 Hz), 64,3, 29.0, 21.9, 13.9 ppm; ESI-HRMS, *m*/*z*: Calcd for C_16_H_17_F_3_NO_4_^+^ [M + H]^+^: 344.1104, found: 344.1120.
(14) Ethyl 2-(2-(3-bromophenyl)oxazol-5-yl)methyl-3,3,3-trifluoro-2-hydroxypropanoate (**3n**): white solid, 70% yield, m.p. 63–65 °C; ^1^H NMR (400 MHz, CDCl_3_), *δ*: 8.09 (s, 1H, ArH), 7.89 (d, *J* = 7.6 Hz, 1H, ArH), 7.56 (d, *J* = 7.6 Hz, 1H, ArH), 7.33–7.28 (m, 1H, ArH), 7.02 (s, 1H, ArH), 4.41–4.36 (m, 2H, OCH_2_), 4.26 (br, 1H, OH), 3.53 (d, *J* = 14.8 Hz, 1H, CH_2_-a), 3.35 (d, *J* = 14.8 Hz, 1H, CH_2_-b), 1.35 (t, *J* = 7.6 Hz, 3H, CH_3_) ppm; ^19^F NMR (376 MHz, CDCl_3_), *δ*: −78.51 ppm; ^13^C NMR (100 MHz, CDCl_3_), *δ*: 168.5, 160.3, 145.5, 133.3, 130.4, 129.1, 129.0, 127.7, 124.7, 122.9, 123.0 (q, *J* = 285.2 Hz), 76.7 (q, *J* = 31.8 Hz), 64.4, 29.0, 14.0 ppm; ESI-HRMS, *m*/*z*: Calcd for C_15_H_14_BrF_3_NO_4_^+^ [M + H]^+^: 408.0053, found: 408.0071.
(15) Ethyl 2-(2-(3,5-dimethylphenyl)oxazol-5-yl)methyl-3,3,3-trifluoro-2-hydroxypro- panoate (**3o**): colorless oil, 78% yield; ^1^H NMR (400 MHz, CDCl_3_), *δ*: 7.59 (s, 1H, ArH), 7.07 (s, 1H, ArH), 6.99 (s, 1H, ArH), 4.40–4.35 (m, 2H, OCH_2_), 4.30 (br, 1H, OH), 3.52 (d, *J* = 14.4 Hz, 1H, CH_2_-a), 3.34 (d, *J* = 14.4 Hz, 1H, CH_2_-b), 2.35 (s, 6H, 2CH_3_), 1.34 (t, *J* = 7.6 Hz, 3H, CH_3_) ppm; ^19^F NMR (376 MHz, CDCl_3_), *δ*: −78.52 ppm; ^13^C NMR (100 MHz, CDCl_3_), *δ*: 168.4, 162.1, 144.7, 138.5, 132.2, 127.1, 126.8, 124.0, 123.0 (q, *J* = 285.2 Hz), 76.7 (q, *J* = 29.1 Hz), 64,2, 29.1, 21.2, 13.9 ppm; ESI-HRMS, *m*/*z*: Calcd for C_17_H_19_F_3_NO_4_^+^ [M + H]^+^: 358.1261, found: 358.1269.
(16) Ethyl 2-(2-(3,5-dichlorophenyl)oxazol-5-yl)methyl-3,3,3-trifluoro-2-hydroxypropan- oate (**3p**): colorless oil, 75% yield; ^1^H NMR (400 MHz, CDCl_3_), *δ*: 7.83 (s, 2H, ArH), 7.41 (s, 1H, ArH), 7.04 (s, 1H, ArH), 4.44–4.36 (m, 2H, OCH_2_), 4.32 (br, 1H, OH), 3.54 (d, *J* = 14.4 Hz, 1H, CH_2_-a), 3.36 (d, *J* = 14.4 Hz, 1H, CH_2_-b), 1.36 (t, *J* = 7.6 Hz, 3H, CH_3_) ppm; ^19^F NMR (376 MHz, CDCl_3_), *δ*: −78.53 ppm; ^13^C NMR (100 MHz, CDCl_3_), *δ*: 168.4, 159.2, 146.1, 135.7, 130.2, 129.7, 127.9, 124.5, 122.5 (q, *J* = 285.0 Hz), 76.7 (q, *J* = 29.2 Hz), 64,4, 29.0, 14.0 ppm; ESI-HRMS, *m*/*z*: Calcd for C_15_H_13_Cl_2_F_3_NO_4_^+^ [M + H]^+^: 398.0168, found: 398.0174.
(17) Ethyl 3,3,3-trifluoro-2-hydroxy-2-(2-(naphthalen-1-yl)oxazol-5-yl)methylpropanoate (**3q**): white solid, 70% yield, m.p. 105–107 °C; ^1^H NMR (400 MHz, CDCl_3_), *δ*: 9.15 (d, *J* = 7.6 Hz, 1H, ArH), 8.10 (d, *J* = 7.6 Hz, 1H, ArH), 7.95 (d, *J* = 8.0 Hz, 1H, ArH), 7.90 (d, *J* = 8.0 Hz, 1H, ArH), 7.65–7.61 (m, 1H, ArH), 7.57–7.6152 (m, 2H, ArH), 7.17 (s, 1H, ArH), 4.45–4.32 (m, 2H, OCH_2_), 4.10 (br, 1H, OH), 3.60 (d, *J* = 14.8 Hz, 1H, CH_2_-a), 3.42 (d, *J* = 14.8 Hz, 1H, CH_2_-b), 1.32 (t, *J* = 7.6 Hz, 3H, CH_3_) ppm; ^19^F NMR (376 MHz, CDCl_3_), *δ*: −78.49 ppm; ^13^C NMR (100 MHz, CDCl_3_), *δ*: 168.6, 161.7, 144.6, 133.9, 131.3, 130.1, 128.6, 127.7, 127.6, 127.5, 126.4, 126.0, 124.9, 123.8, 123.1 (q, *J* = 285.0 Hz), 76.8 (q, *J* = 29.2 Hz), 64.4, 29.0, 14.0 ppm; ESI-HRMS, *m*/*z*: Calcd for C_19_H_17_F_3_NO_4_^+^ [M + H]^+^: 380.1104, found: 380.1103.
(18) Ethyl 3,3,3-trifluoro-2-hydroxy-2-(2-(naphthalen-2-yl)oxazol-5-yl)methylpropanoate (**3r**): white solid, 73% yield, m.p. 111–113 °C, 73% yield; ^1^H NMR (400 MHz, CDCl_3_), *δ*: 8.36 (s, 1H, ArH), 7.96 (d, *J* = 8.4 Hz, 1H, ArH), 7.80 (d, *J* = 8.4 Hz, 2H, ArH), 7.76–7.74 (m, 1H, ArH), 7.45–7.43 (m, 2H, ArH), 6.98 (s, 1H, ArH), 4.35–4.29 (m, 3H, OCH_2_, OH), 3.50 (d, *J* = 15.2 Hz, 1H, CH_2_-a), 3.31 (d, *J* = 15.2 Hz, 1H, CH_2_-b), 1.27 (t, *J* = 7.6 Hz, 3H, CH_3_) ppm; ^19^F NMR (376 MHz, CDCl_3_), *δ*: −78.46 ppm; ^13^C NMR (100 MHz, CDCl_3_), *δ*: 168.5, 161.9, 145.1, 134.1, 133.0, 128.7, 128.7, 127.9, 127.6, 127.4, 126.8, 126.2, 124.5, 123.1, 123.1 (q, *J* = 285.3 Hz), 76.8 (q, *J* = 29.2 Hz), 64.3, 29.2, 14.0 ppm; ESI-HRMS, *m*/*z*: Calcd for C_19_H_17_F_3_NO_4_^+^ [M + H]^+^: 380.1104, found: 380.1103.
(19) Ethyl 2-(2-(2-chloropyridin-3-yl)oxazol-5-yl)methyl-3,3,3-trifluoro-2-hydroxy-pro- panoate (**3s**): white solid, 62% yield, m.p. 92–94 °C; ^1^H NMR (400 MHz, CDCl_3_), *δ*: 8.43–4.41 (m, 1H, ArH), 8.25–8.20 (m, 1H, ArH), 7.34–7.28 (m, 1H, ArH), 7.07 (s, 1H, ArH), 4.37–4.31 (m, 2H, OCH_2_), 4.13 (br, 1H, OH), 3.55–3.48 (m, 1H, CH_2_), 3.37–3.31 (m, 1H, CH_2_), 1.26 (t, *J* = 7.6 Hz, 3H, CH_3_) ppm; ^19^F NMR (376 MHz, CDCl_3_), *δ*: -76.51 ppm; ^13^C NMR (100 MHz, CDCl_3_), *δ*: 168.4, 157.9, 150.6, 148.5, 146.4, 139.4, 127.9, 123.3, 123.1 (q, *J* = 285.2 Hz), 122.4, 76.8 (q, *J* = 29.1 Hz), 64.5, 29.0, 13.9 ppm; ESI-HRMS, *m*/*z*: Calcd for C_14_H_13_ClF_3_N_2_O_4_^+^ [M + H]^+^: 365.0510, found: 365.0536.
(20) Ethyl 3,3,3-trifluoro-2-(2-(furan-2-yl)oxazol-5-yl)methyl-2-hydroxypropanoate (**3t**): colorless oil, 68% yield; ^1^H NMR (400 MHz, CDCl_3_), *δ*: 7.54 (s, 1H, ArH), 7.01 (s, 1H, ArH), 6.96 (d, *J* = 4.8 Hz, 1H, ArH), 6.52 (s, 1H, ArH), 4.40 (q, 2H, *J* = 7.6 Hz, OCH_2_), 4.19 (br, 1H, OH), 3.52 (d, *J* = 15.2 Hz, 1H, CH_2_-a), 3.33 (d, *J* = 15.2 Hz, 1H, CH_2_-b), 1.37 (t, *J* = 7.6 Hz, 3H, CH_3_) ppm; ^19^F NMR (376 MHz, CDCl_3_), *δ*: −78.54 ppm; ^13^C NMR (100 MHz, CDCl_3_), *δ*: 168.4, 154.5, 144.4, 142.7, 127.3, 123.0 (q, *J* = 285.3 Hz), 76.5 (q, *J* = 29.2 Hz), 111.9, 111.4, 64,4, 28.9, 13.9 ppm; ESI-HRMS, *m*/*z*: Calcd for C_13_H_13_F_3_NO_5_^+^ [M + H]^+^: 320.0740, found: 320.0751.
(21) Ethyl 3,3,3-trifluoro-2-hydroxy-2-(2-(thiophen-2-yl)oxazol-5-yl)methylpropanoate (**3u**): white solid, 62% yield; m.p. 100–102 °C; ^1^H NMR (400 MHz, CDCl_3_), *δ*: 7.60 (d, *J* = 5.6 Hz, 1H, ArH), 7.42 (d, *J* = 5.6 Hz, 1H, ArH), 7.11–7.09 (m, 1H, ArH), 6.98 (s, 1H, ArH), 4.45–4.37 (m, 2H, OCH_2_), 4.07 (br, 1H, OH), 3.52 (d, *J* = 14.8 Hz, 1H, CH_2_-a), 3.32 (d, *J* = 14.8 Hz, 1H, CH_2_-b), 1.37 (t, *J* = 7.6 Hz, 3H, CH_3_) ppm; ^19^F NMR (376 MHz, CDCl_3_), *δ*: −78.53 ppm; ^13^C NMR (100 MHz, CDCl_3_), *δ*: 168.5, 157.9, 144.3, 129.7, 128.3, 128.0, 127.6, 127.5, 123.0 (q, *J* = 285.3 Hz), 76.8 (q, *J* = 29.1 Hz), 64.4, 29.0, 14.0 ppm; ESI-HRMS, *m*/*z*: Calcd for C_13_H_13_F_3_NO_4_S^+^ [M + H]^+^: 336.0512, found: 336.0518.
(22) Ethyl 2-(2-(5-chlorothiophen-2-yl)oxazol-5-yl)methyl-3,3,3-trifluoro-2-hydrox-pro- panoate (**3v**): colorless oil, 56% yield; ^1^H NMR (400 MHz, CDCl_3_), *δ*: 7.29 (d, *J* = 4.0 Hz, 1H, ArH), 6.88 (s, 1H, ArH), 6.84 (d, *J* = 4.0 Hz, 1H, ArH), 4.38–4.26 (m, 2H, OCH_2_), 4.06 (br, 1H, OH), 3.42 (d, *J* = 15.2 Hz, 1H, CH_2_-a), 3.24 (d, *J* = 15.2 Hz, 1H, CH_2_-b), 1.28 (t, *J* = 7.2 Hz, 3H, CH_3_) ppm; ^19^F NMR (376 MHz, CDCl_3_), *δ*: −78.53 ppm; ^13^C NMR (100 MHz, CDCl_3_), *δ*: 168.4, 156.8, 144.7, 133.4, 128.1, 127.5, 127.2, 126.8, 123.0 (q, *J* = 285.8 Hz), 76.5 (q, *J* = 29.1 Hz), 64.4, 28.9, 14.0 ppm; ESI-HRMS, *m*/*z*: Calcd for C_13_H_12_ClF_3_NO_4_S^+^ [M + H]^+^: 370.0122, found: 370.0138.
(23) Ethyl 2-(2-benzyloxazol-5-yl)methyl-3,3,3-trifluoro-2-hydroxypropanoate (**3w**): colorless oil, 59% yield; ^1^H NMR (400 MHz, CDCl_3_), *δ*: 7.30–7.27 (m, 2H, ArH), 7.23–7.19 (m, 1H, ArH), 7.16 (d, *J* = 7.6 Hz, 2H, ArH), 7.15 (s, 1H, ArH), 4.39–4.32 (m, 2H, OCH_2_), 4.13 (br, 1H, OH), 3.40 (d, *J* = 15.2 Hz, 1H, CH_2_-a), 3.23 (d, *J* = 15.2 Hz, 1H, CH_2_-b), 3.02 (s, *2*H, PhCH_2_), 1.33 (t, *J* = 7.6 Hz, 3H, CH_3_) ppm; ^19^F NMR (376 MHz, CDCl_3_), *δ*: −78.52 ppm; ^13^C NMR (100 MHz, CDCl_3_), *δ*: 168.5, 164.3, 144.6, 140.0, 128.6, 128.3, 126.4, 125.6, 123.0 (q, *J* = 285.6 Hz), 76.6 (q, *J* = 29.3 Hz), 64.2, 33.0, 28.9, 13.9 ppm. ESI-HRMS, *m*/*z*: Calcd for C_16_H_17_F_3_NO_4_^+^ [M + H]^+^: 344.1104, found: 344.1118.
(24) Ethyl (*E*)-3,3,3-trifluoro-2-hydroxy-2-(2-styryloxazol-5-yl)methylpropanoate (**3x**): colorless oil, 51% yield; ^1^H NMR (400 MHz, CDCl_3_), *δ*: 7.51 (d, *J* = 7.6 Hz, 2H, ArH), 7.44–7.32 (m, 4H, ArH), 6.97 (s, 1H, ArH), 6.88 (d, *J* = 15.2 Hz, 1H, =CH), 4.40 (q, *J* = 7.6 Hz, 2H, OCH_2_), 4.13 (br, 1H, OH), 3.50 (d, *J* = 14.8 Hz, 1H, CH_2_-a), 3.32 (d, *J* = 14.8 Hz, 1H, CH_2_-b), 1.37 (t, *J* = 7.6 Hz, 3H, CH_3_) ppm; ^19^F NMR (376 MHz, CDCl_3_), *δ*: −78.51 ppm; ^13^C NMR (100 MHz, CDCl_3_), *δ*: 168.5, 161.6, 144.5, 136.1, 135.3, 129.3, 128.9, 127.6, 127.2, 123.0 (q, *J* = 285.4 Hz), 113.7, 76.7 (q, *J* = 29.3 Hz), 64,3, 29.0, 14.0 ppm; ESI-HRMS, *m*/*z*: Calcd for C_17_H_17_F_3_NO_4_^+^ [M + H]^+^: 356.1104, found: 356.1132.
(25) Ethyl 3,3,3-trifluoro-2-hydroxy-2-(2-(4-(5-methyloxazol-2-yl)phenyl)oxazol-5-yl)- methylpropanoate (**3y**): colorless oil, 48% yield; ^1^H NMR (400 MHz, CDCl_3_), *δ*: 8.00 (d, *J* = 9.2 Hz, 2H, ArH), 7.95 (d, *J* = 9.2 Hz, 2H, ArH), 6.98 (s, 1H, ArH), 6.81 (s, 1H, ArH), 4.38–4.26 (m, 3H, OCH_2_, OH), 3.48 (d, *J* = 15.2 Hz, 1H, CH_2_-a), 3.30 (d, *J* = 15.2 Hz, 1H, CH_2_-b), 2.33 (s, 3H, CH_3_), 1.37 (t, *J* = 7.6 Hz, 3H, CH_3_) ppm; ^19^F NMR (376 MHz, CDCl_3_), *δ*: −78.51 ppm; ^13^C NMR (100 MHz, CDCl_3_), *δ*: 167.4, 160.1, 148.6, 144.4, 128.1, 127.1, 126.7, 125.4, 125.3, 123.6, 123.0, 122.0 (q, *J* = 285.6 Hz), 75.6 (q, *J* = 29.2 Hz), 63.3, 28.1, 13.0, 10.1 ppm; ESI-HRMS, *m*/*z*: Calcd for C_19_H_18_F_3_N_2_O_5_^+^ [M + H]^+^: 411.1162, found: 411.1157.
(26) Methyl 3,3,3-trifluoro-2-hydroxy-2-(2-phenyloxazol-5-yl)methylpropanoate (**3z**): colorless oil, 81% yield; ^1^H NMR (400 MHz, CDCl_3_), *δ*: 7.96–7.94 (m, 2H, ArH), 7.44–7.43 (m, 3H, ArH), 7.00 (s, 1H, ArH), 4.37 (br, 1H, OH), 3.94 (s, 1H, OCH_3_), 3.53 (d, *J* = 15.2 Hz, 1H, CH_2_-a), 3.34 (d, *J* = 15.2 Hz, 1H, CH_2_-b) ppm; ^19^F NMR (376 MHz, CDCl_3_), *δ*: −78.49 ppm; ^13^C NMR (100 MHz, CDCl_3_), *δ*: 169.0, 161.8, 144.8, 130.4, 128.8, 127.5, 127.2, 126.2, 123.0 (q, *J* = 285.0 Hz), 76.8 (q, *J* = 28.7 Hz), 54.5, 29.2 ppm; ESI-HRMS, *m*/*z*: Calcd for C_14_H_13_F_3_NO_4_^+^ [M + H]^+^: 316.0791, found: 316.0808.
(27) 1,1,1,3,3,3-Hexafluoro-2-(2-phenyloxazol-5-yl)methylpropan-2-ol (**3aa**): colorless oil, 86% yield; ^1^H NMR (400 MHz, CDCl_3_), *δ*: 7.83 (d, *J* = 7.6 Hz, 2H, ArH), 7.37–7.29 (m, 3H, ArH), 6.90 (s, 1H, ArH), 6.49 (br, 1H, OH), 3.31 (s, 2H, CH_2_) ppm; ^19^F NMR (376 MHz, CDCl_3_), *δ*: −76.51 ppm; ^13^C NMR (100 MHz, CDCl_3_), *δ*: 162.3, 143.6, 130.8, 128.8, 127.9, 126.4, 126.4, 122.8 (q, *J* = 286.3 Hz), 76.5–74.7 (m), 27.3 ppm; ESI-HRMS, *m*/*z*: Calcd for C_13_H_10_F_6_NO_2_^+^ [M + H]^+^: 326.0610, found: 326.0637.
(28) 1,1,1,3,3,3-Hexafluoro-2-(2-(4-fluorophenyl)oxazol-5-yl)methylpropan-2-ol (**3ab**): colorless oil, 80% yield; ^1^H NMR (400 MHz, CDCl_3_), *δ*: 7.93–7.90 (m, 2H, ArH), 7.13–7.09 (m, 2H, ArH), 7.01 (s, 1H, ArH), 5.38 (br, 1H, OH), 3.41 (s, 2H, CH_2_) ppm; ^19^F NMR (376 MHz, CDCl_3_), *δ*: −76.67, −108.45 ppm; ^13^C NMR (100 MHz, CDCl_3_), *δ*: 164.3 (d, *J* = 251.4 Hz), 161.6, 143.3, 128.5 (d, *J* = 9.3 Hz), 128.3, 122.9 (d, *J* = 3.3 Hz), 122.7 (q, *J* = 285.8 Hz), 116.1 (d, *J* = 22.5 Hz), 76.2–75.0 (m), 27.2 ppm; ESI-HRMS, *m*/*z*: Calcd for C_13_H_9_F_7_NO_2_^+^ [M + H]^+^: 344.0516, found: 344.0513.
(29) 1,1,1,3,3,3-Hexafluoro-2-(2-(4-(trifluoromethyl)phenyl)oxazol-5-yl)methylpropan-2-ol (**3ac**): white solid, 76% yield, m.p. 121–123 °C; ^1^H NMR (400 MHz, CDCl_3_), *δ*: 8.05 (d, *J* = 7.6 Hz, 2H, ArH), 7.68 (d, *J* = 7.6 Hz, 2H, ArH), 7.11 (s, 1H, ArH), 4.91 (br, 1H, OH), 3.45 (s, 2H, CH_2_) ppm; ^19^F NMR (376 MHz, CDCl_3_), *δ*: -63.02, -76.69 ppm; ^13^C NMR (100 MHz, CDCl_3_), *δ*: 160.9, 144.1, 132.3 (q, *J* = 31.8 Hz), 129.7, 128.8, 126.5, 125.9 (q, *J* = 3.6 Hz), 123.7 (q, *J* = 270.6 Hz), 122.6 (q, *J* = 287.7 Hz), 76.2–75.0 (m), 27.1 ppm; ESI-HRMS, *m*/*z*: Calcd for C_14_H_9_F_9_NO_2_^+^ [M + H]^+^: 394.0484, found: 394.0498.
(30) 4-(5-(3,3,3-Trifluoro-2-hydroxy-2-trifluoromethylpropyl)oxazol-2-yl)benzonitrile (**3ad**): white solid, 78% yield, m.p. 122–124 °C; ^1^H NMR (400 MHz, CDCl_3_), *δ*: 8.06 (d, *J* = 8.0 Hz, 2H, ArH), 7.71 (d, *J* = 8.0 Hz, 2H, ArH), 7.14 (s, 1H, ArH), 5.63 (br, 1H, OH), 3.45 (s, 2H, CH_2_) ppm; ^19^F NMR (376 MHz, CDCl_3_), *δ*: −76.51 ppm; ^13^C NMR (100 MHz, CDCl_3_), *δ*: 160.3, 144.9, 132.7, 130.4, 128.9, 126.7, 122.7 (q, *J* = 285.4 Hz), 118.0, 113.9, 76.2–75.0 (m), 27.2 ppm; ESI-HRMS, *m*/*z*: Calcd for C_14_H_9_F_6_N_2_O_2_^+^ [M + H]^+^: 351.0563, found: 351.0596.
(31) 2-(2-(3,5-Dimethylphenyl)oxazol-5-yl)methyl-1,1,1,3,3,3-hexafluoropropan-2-ol (**3ae**): colorless oil, 75% yield; ^1^H NMR (400 MHz, CDCl_3_), *δ*: 7.44 (s, 2H, ArH), 6.99 (s, 1H, ArH), 6.87 (s, 1H, ArH), 5.95 (br, 1H, OH), 3.32 (s, 2H, CH_2_), 2.25 (s, 6H, 2CH_3_)ppm; ^19^F NMR (376 MHz, CDCl_3_), *δ*: −76.69 ppm; ^13^C NMR (100 MHz, CDCl_3_), *δ*: 162.7, 143.0, 138.5, 132.6, 128.0, 126.1, 124.1, 122.8 (q, *J* = 285.6 Hz), 76.3–75.1 (m), 27.2, 21.1 ppm; ESI-HRMS, *m*/*z*: Calcd for C_15_H_14_F_6_NO_2_^+^ [M + H]^+^: 354.0923, found: 354.0935.
(32) 2-(2-(5-Chlorothiophen-2-yl)oxazol-5-yl)methyl-1,1,1,3,3,3-hexafluoropropan-2-ol (**3af**): white solid, 56% yield, m.p. 101–103 °C; ^1^H NMR (400 MHz, CDCl_3_), *δ*: 7.40 (d, *J* = 6.8 Hz, 1H, ArH), 6.94 (s, 1H, ArH), 6.91 (d, *J* = 6.8 Hz, 1H, ArH), 5.80 (br, 1H, OH), 3.38 (s, 2H, CH_2_) ppm; ^19^F NMR (376 MHz, CDCl_3_), *δ*: −76.61 ppm; ^13^C NMR (100 MHz, CDCl_3_), *δ*: 157.3, 143.2, 134.2, 128.0, 127.7, 127.3, 127.1, 122.7 (q, *J* = 285.2 Hz), 76.2–75.0 (m), 27.1 ppm; ESI-HRMS, *m*/*z*: Calcd for C_11_H_7_ClF_6_NO_2_S^+^ [M + H]^+^: 365.9785, found: 365.9803.
(33) 5-Methyl-2-phenyloxazole (**4a**) [45]: colorless oil, 87% yield; ^1^H NMR (400 MHz, CDCl_3_), *δ*: 8.0 (d, *J* = 7.2 Hz, 2H, ArH), 7.46–7.40 (m, 3H, ArH), 6.84 (s, 1H, ArH), 2.37 (s, 3H, CH_3_) ppm.
(34) 5-Methylene-2-phenyl-4,5-dihydrooxazole (**5a**) [45]: colorless oil, 83% yield; ^1^H NMR (400 MHz, CDCl_3_), *δ*: 7.96 (d, *J* = 7.2 Hz, 1H, ArH), 7.46 (t, *J* = 7.6 Hz, 1H, ArH), 7.41–7.38 (m, 2H, ArH), 4.79 (dd, *J* = 6.0, 2.8 Hz, 1H, =CH_2_-a), 4.61 (t, *J* = 2.8 Hz, 2H, CH_2_), 4.33 (dd, *J* = 6.0, 2.8 Hz, 1H, =CH_2_-b) ppm.

The detailed spectra of ^1^H, ^13^C NMR, and ^19^F NMR for all compounds **3a**–**3af**, **4a**, and **5a** are provided in the Supplementary Materials.

## 4. Conclusions

In summary, Zn(OTf)_2_-catalyzed tandem cycloisomerization/hydroxyalkylation has been developed for the concise synthesis of potentially bioactive oxazoles containing a unit of CF_3_-substituted alcohol from *N*-propargylamides with trifluoropyruvates. This newly developed protocol is operationally simple and results in a wide range of oxazoles obtained in moderate to good yields. The method’s broad substrate scope, high functional group tolerance, and high atom economy are positive features. Additionally, the method enables gram-scale reaction with excellent yield, indicating its strong potential for practical application. This synthesis strategy will be helpful for the further design and rapid synthesis of structurally diverse CF_3_-containing oxazole derivatives.

## Figures and Tables

**Table 1:** Optimization of reaction conditions ^[a]^.

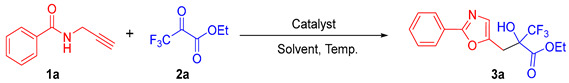				
Entry	Catalyst	Solvent	Temp. (°C)	Yield of 3a (%) ^[b]^
1	-	DCE	70	Trace
2	FeCl_3_ (20 mol%)	DCE	70	56
3	InCl_3_ (20 mol%)	DCE	70	80
4	Y(OTf)_3_ (20 mol%)	DCE	70	Trace
5	Sc(OTf)_3_ (20 mol%)	DCE	70	Trace
6	In(OTf)_3_ (20 mol%)	DCE	70	78
7	Cu(OTf)_2_ (20 mol%)	DCE	70	32
8	Zn(OTf)_2_ (20 mol%)	DCE	70	83
9	TfOH (20 mol%)	DCE	70	Trace
10	Zn(OTf)_2_ (10 mol%)	DCE	70	66
11	Zn(OTf)_2_ (15 mol%)	DCE	70	84
12	Zn(OTf)_2_ (25 mol%)	DCE	70	80
13	Zn(OTf)_2_ (15 mol%)	Toluene	70	76
14	Zn(OTf)_2_ (15 mol%)	1,4-Dioxane	70	12
15	Zn(OTf)_2_ (15 mol%)	Acetonitrile	70	35
16	Zn(OTf)_2_ (15 mol%)	Chloroform	70	62
17	Zn(OTf)_2_ (15 mol%)	THF	70	Trace
18	Zn(OTf)_2_ (15 mol%)	DMF	70	Trace
19	Zn(OTf)_2_ (15 mol%)	DCE	50	56
20	Zn(OTf)_2_ (15 mol%)	DCE	90	73
21 ^[c]^	Zn(OTf)_2_ (15 mol%)	DCE	70	72
22 ^[d]^	Zn(OTf)_2_ (15 mol%)	DCE	70	83

^[a]^ Reaction conditions: **1a** (0.3 mmol); **2a** (0.36 mmol, 1.2 equiv.); catalyst; temperature; solvent (1.5 mL); 12 h. ^[b]^ Isolated yield based on **1a**. DCE = 1,2-dichloroethane; THF = tetrahydrofuran; DMF = *N*,*N*-dimethylformamide. ^[c]^ 6 h. ^[d]^ 18 h.

**Table 2:** Substrate scope of various *N*-propargylamides **1** ^[a,b]^.

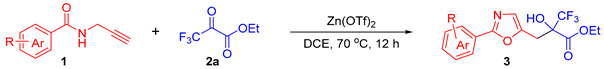
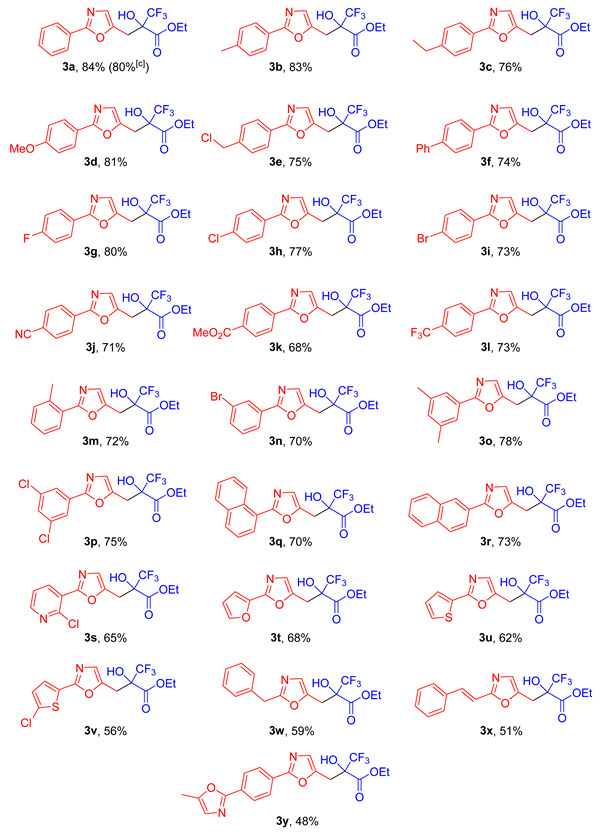

^[a]^ Reaction conditions: **1** (0.3 mmol), **2a** (0.36 mmol, 1.2 equiv.), Zn(OTf)_2_ (0.045 mmol, 15 mol%), DCE (1.5 mL), 70 °C, and 12 h. ^[b]^ Isolated yields based on **1**. ^[c]^ Reaction performed on a 4.5 mmol scale: **1a** (4.5 mmol), **2a** (5.4 mmol), Zn(OTf)_2_ (0.675 mmol, 15 mol%), DCE (25 mL), 70 °C, and 24 h.

**Table 3:** Substrate scope of various trifluoroacetyl compounds **2** ^[a,b]^.

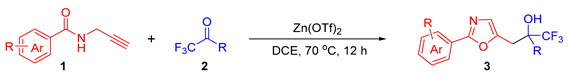
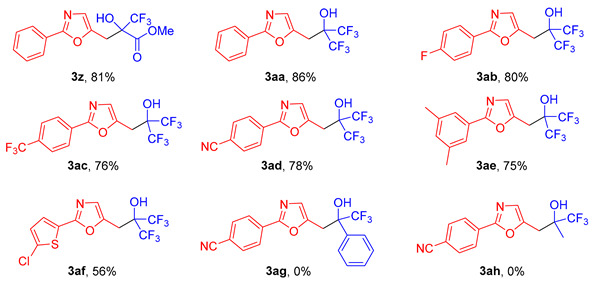

^[a]^ Reaction conditions: **1** (0.3 mmol), **2** (0.36 mmol, 1.2 equiv.), Zn(OTf)_2_ (0.045 mmol, 15 mol%), DCE (1.5 mL), 70 °C, and 12 h. ^[b]^ Isolated yields based on **1**.

**Table 4:** Synthesis of **1a**–**1y** ^[a,b]^.

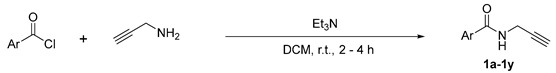
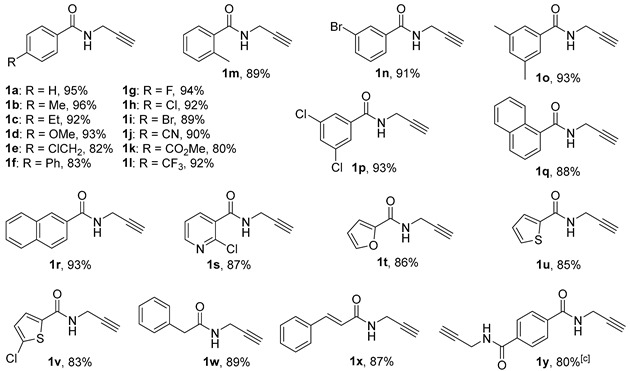

^[a]^ Reaction conditions: acid chloride (10 mmol), propargylamine (10 mmol), Et_3_N (12 mmol), DCM (20 mL), 0 °C, 15 min, r.t., 2–4 h. ^[b]^ Isolated yields. ^[c]^ Reaction conditions: terephthaloyl dichloride (10 mmol), propargylamine (20 mmol), Et_3_N (22 mmol), DCM (30 mL), 0 °C, 15 min, rt, 4 h.

## Data Availability

All data supporting the findings of this study are available within the paper and its Supplementary Materials, which are published online.

## Supplementary Materials

The following supporting information can be downloaded at https://www.mdpi.com/article/10.3390/molecules29245848/s1, which contains details on the ^1^H, ^13^C, and ^19^F NMR spectra for all compounds.
