# Supplementary material for: Synthesis of Oxazoles Containing CF3-Substituted Alcohol Unit via Tandem Cycloisomerization/Hydroxyalkylation from N-Propargylamides with Trifluoropyruvates
Source: Molecules. 2024 Dec 11;29(24):5848. doi: 10.3390/molecules29245848 (PMC11728596; doi:10.3390/molecules29245848)
Supplement: Supplementary file 1 [file molecules-29-05848-s001.zip › molecules-3354526-supplementary.pdf]

Supplementary Materials

Synthesis of Oxazoles Containing CF_3-substituted Alcohol Unit via Tandem Cycloisomerization/Hydroxyalkylation from *N*-Propargylamides with Trifluoropyruvates

Juan-Juan Gao[1,2], Long-Hui Wu[1], Shu-Qin Yu[1], Xue Zhu[1], Yu Zeng[2], Kai Yang[1,2,*], Zhao-Yang Wang[2,*]

[1] Jiangxi Province Key Laboratory of Pharmacology of Traditional Chinese Medicine, College of Pharmacy, Gannan Medical University, Ganzhou 341000, China; gaoya0758@163.com (J.-J.G.); 13970997583@163.com (L.-H.W.); yu20241211@163.com (S.-Q.Y.); 14760556396@139.com (X.Z.)

[2] School of Chemistry, South China Normal University, Guangzhou Key Laboratory of Analytical Chemistry for Biomedicine, GDMPA Key Laboratory for Process Control and Quality Evaluation of Chiral Pharmaceuticals, Key Laboratory of Theoretical Chemistry of Environment, Ministry of Education, Guangzhou 510006, China; 2023022534@m.scnu.edu.cn

* Correspondence: kai_yang@gmu.edu.cn (K.Y.); wangzy@scnu.edu.cn (Z.-Y.W.); Tel.: +86-0797-8169782 (K.Y.); +86-020-3931-0258 (Z.-Y.W.); Fax: +86-020-3931-0187 (Z.-Y.W.)

Table of Contents

^1H, ^{13}C and ^{19}F NMR Spectra for All Products **3a-3af**, **4a** and **5a**..................................[2-50]

NMR Spectra for All Compounds 3a-3af, 4a and 5a

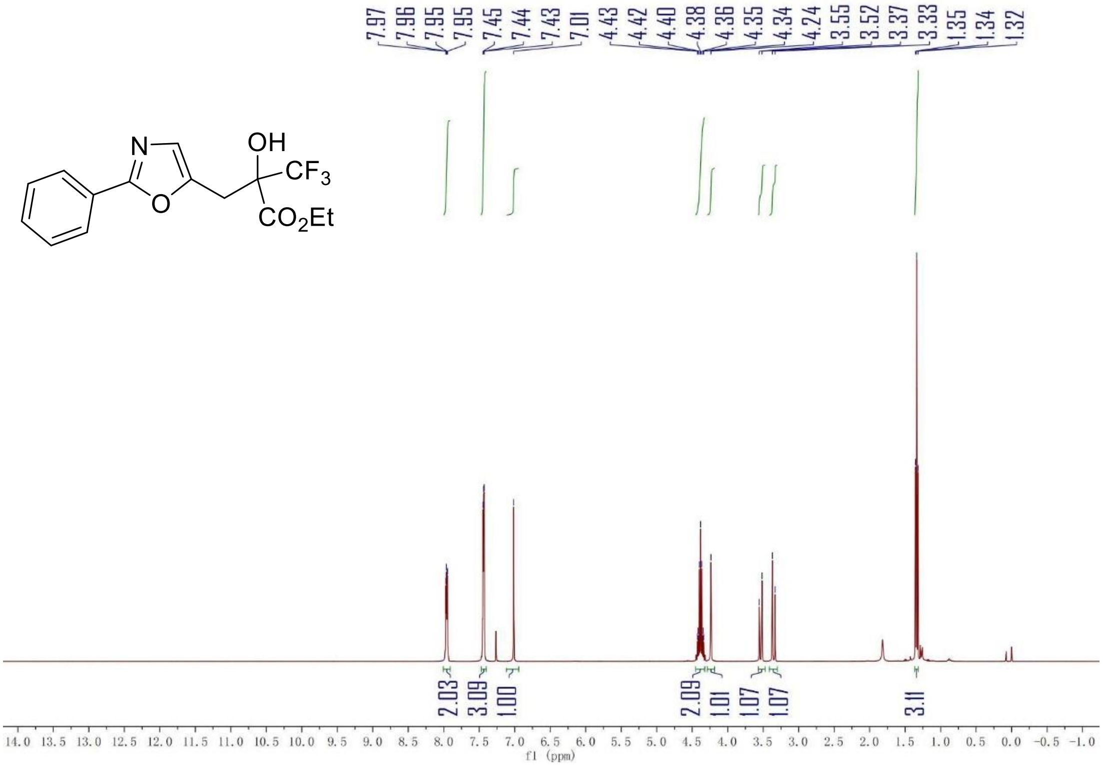

^{1}H NMR spectrum of compound **3a**

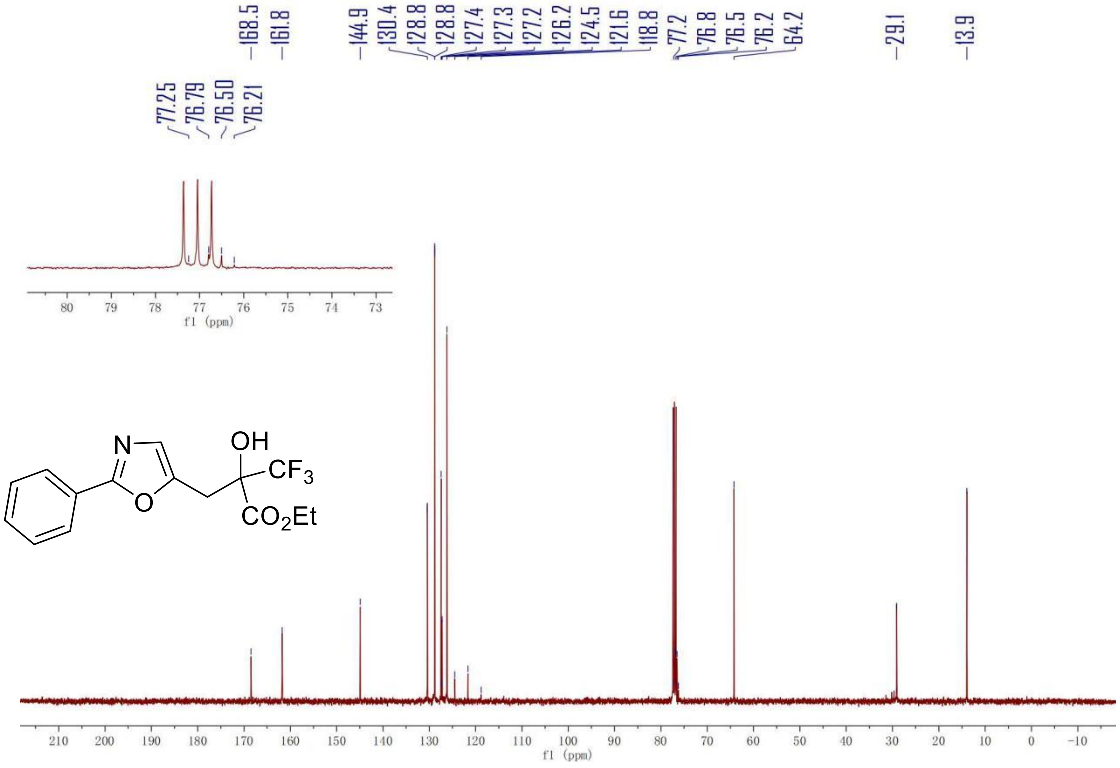

^{13}C NMR spectrum of compound **3a**

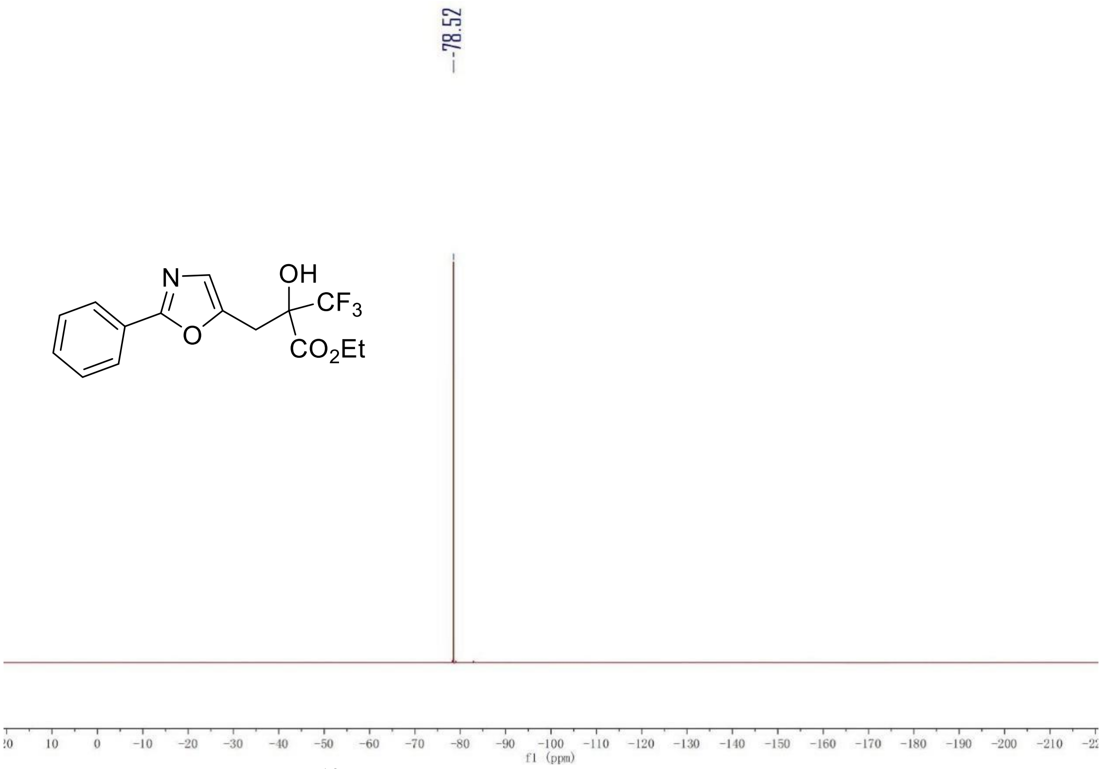

^{19}F NMR spectrum of compound **3a**

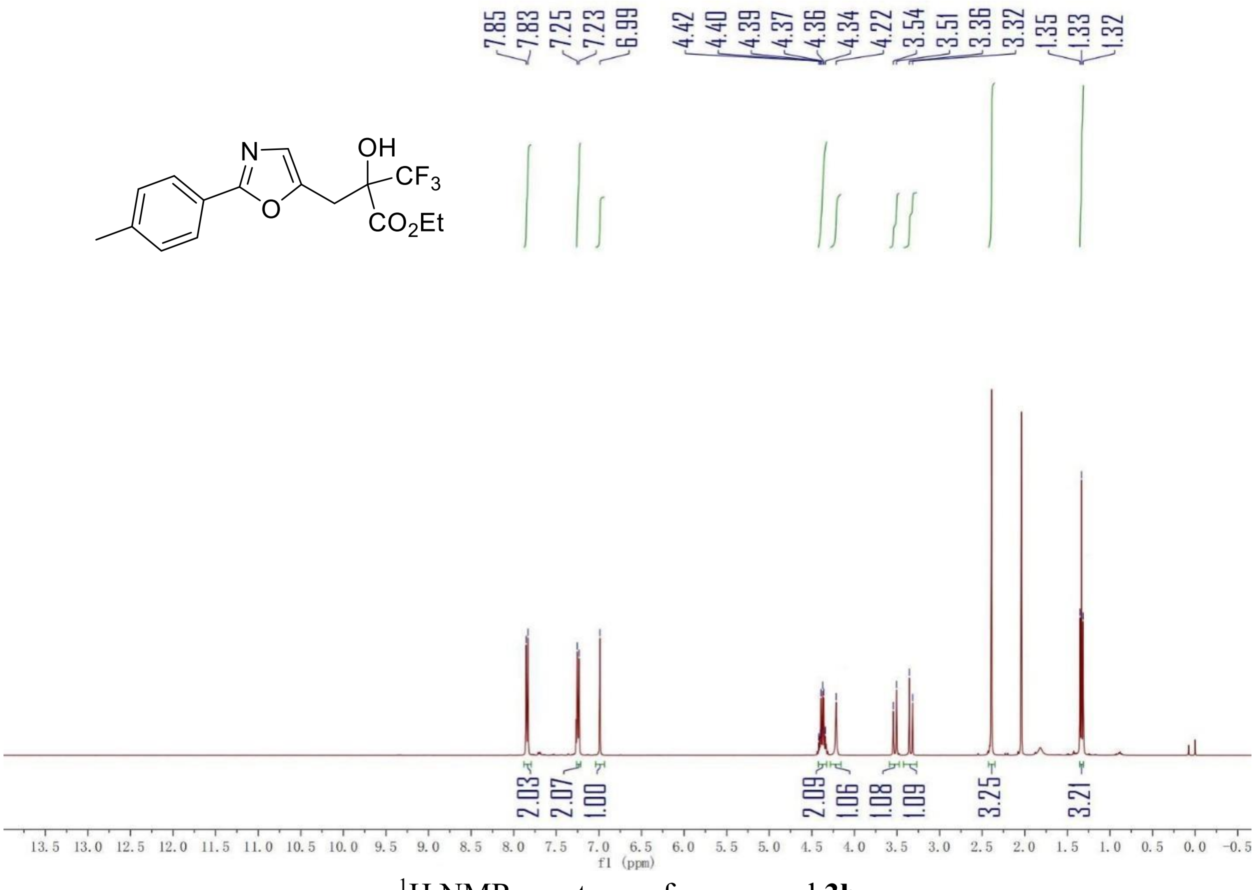

^{1}H NMR spectrum of compound **3b**

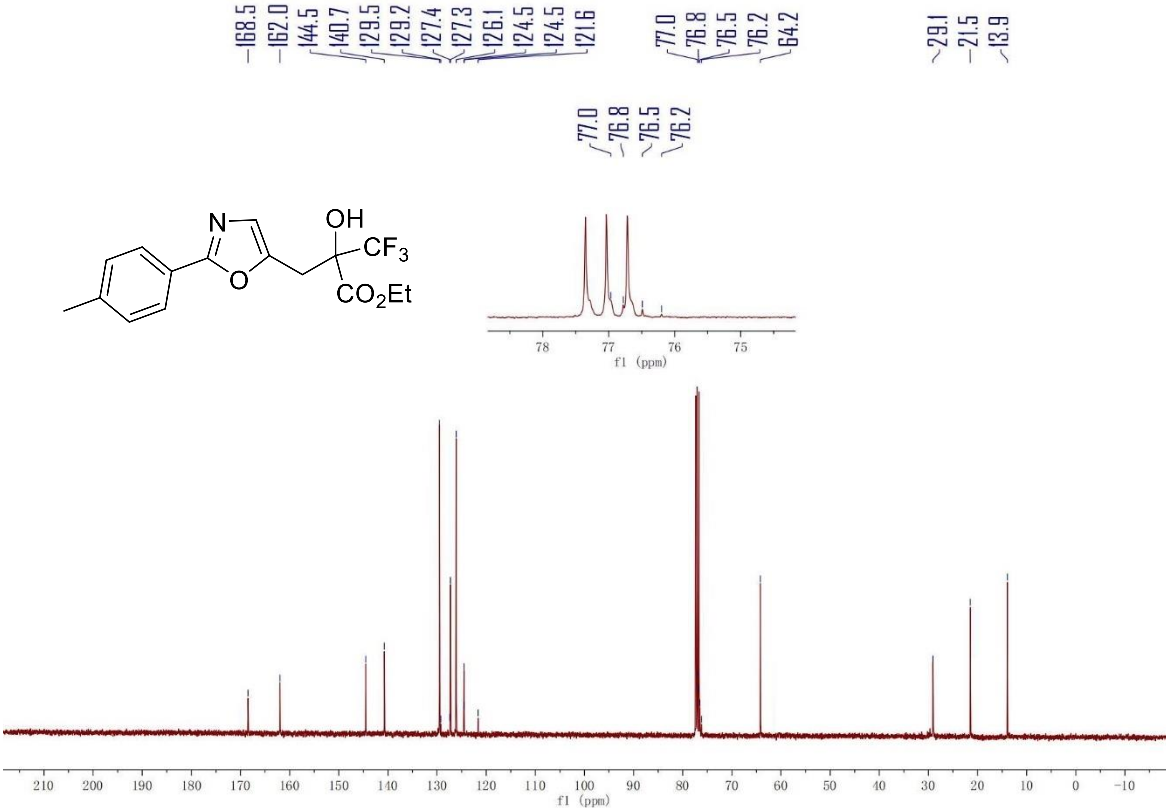

^{13}C NMR spectrum of compound **3b**

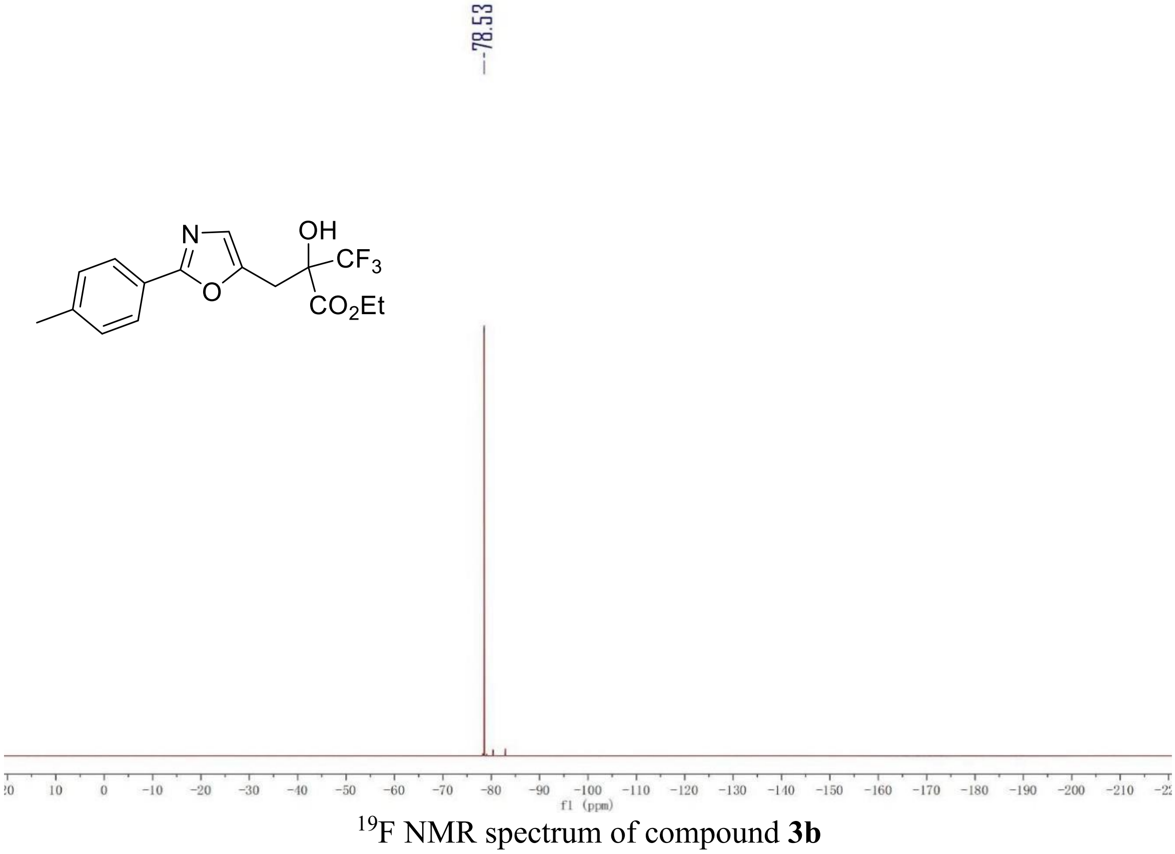

^{19}F NMR spectrum of compound **3b**

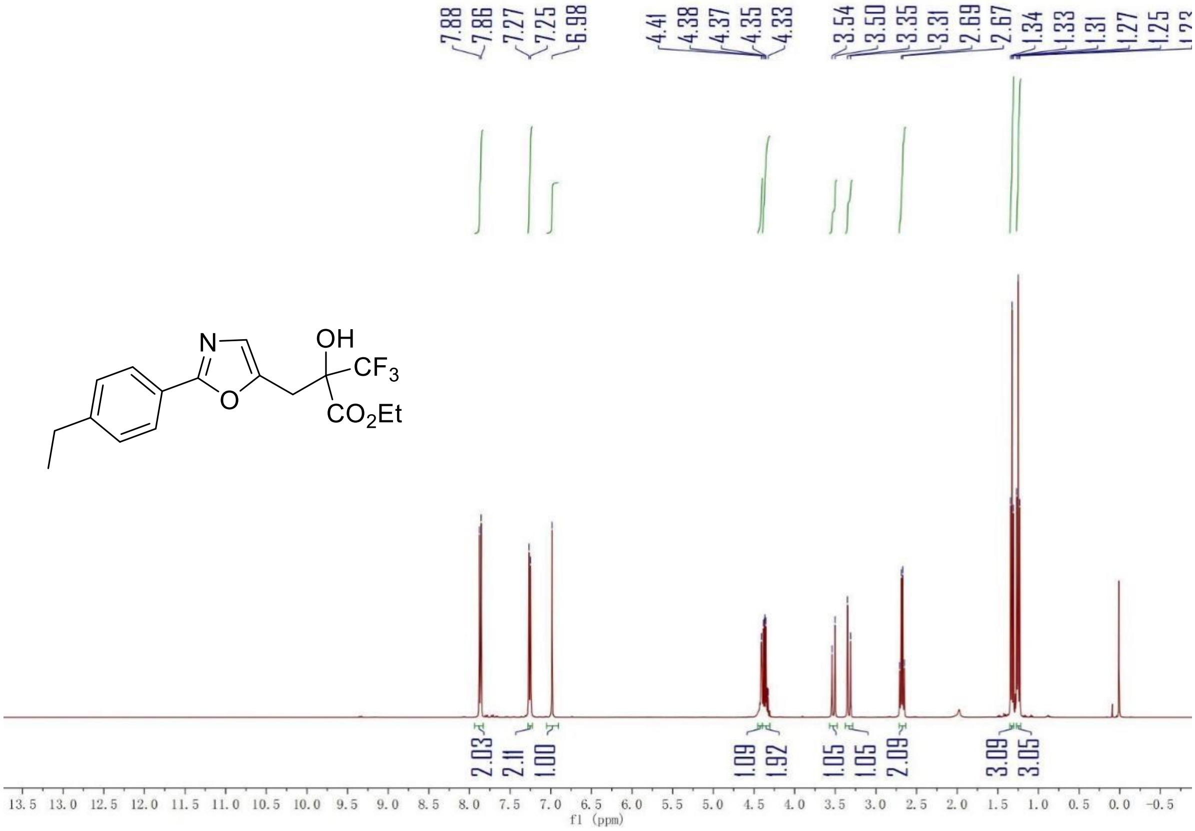

^{1}H NMR spectrum of compound **3c**

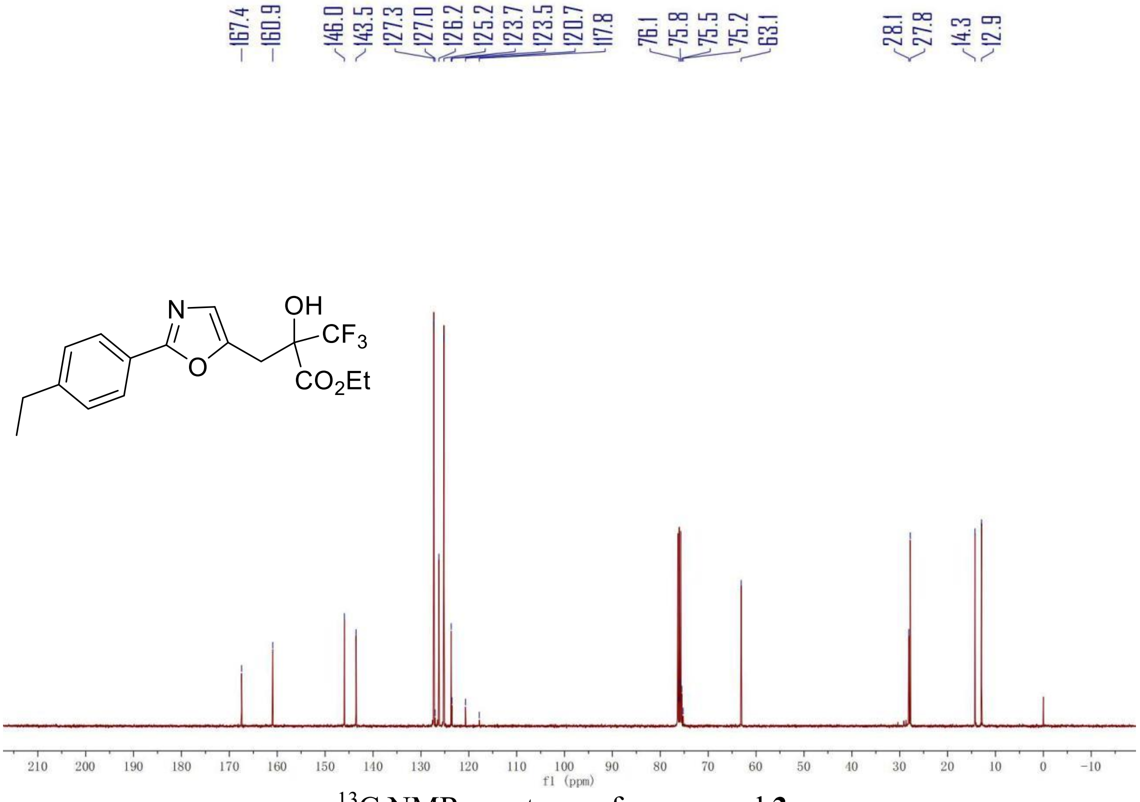

^{13}C NMR spectrum of compound **3c**

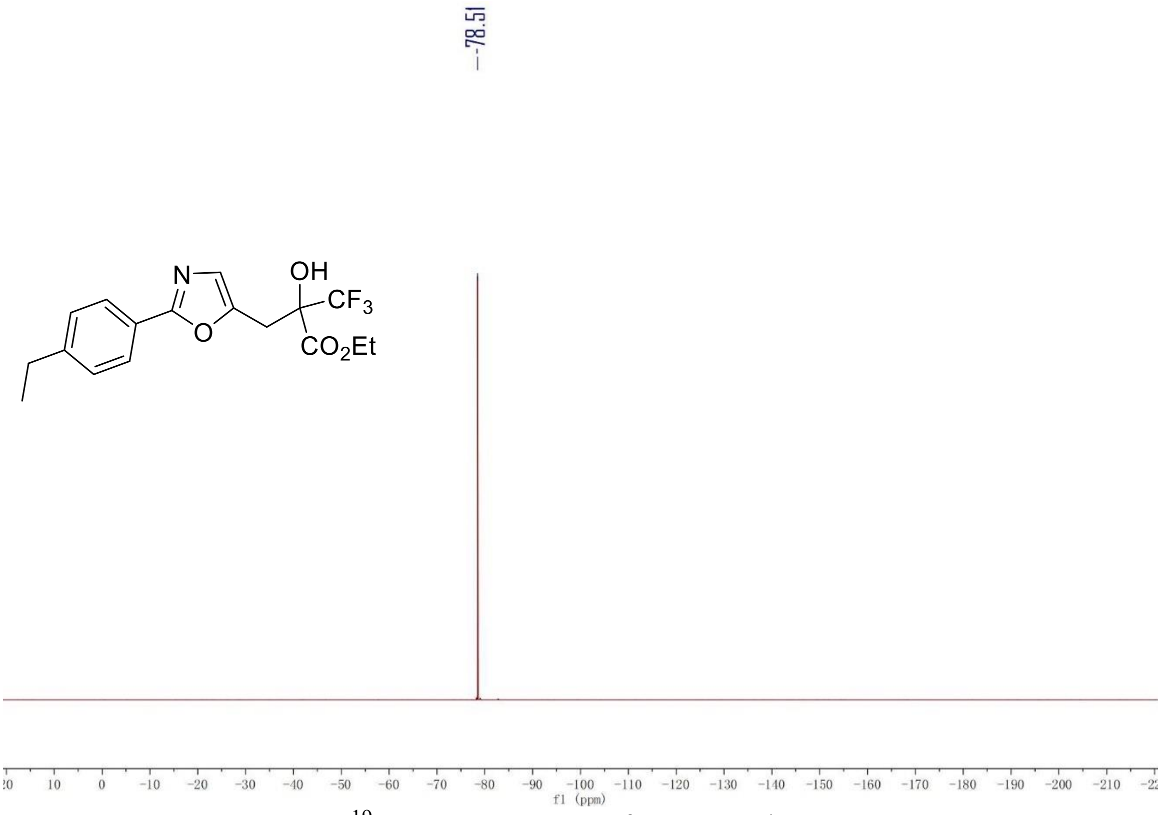

^{19}F NMR spectrum of compound **3c**

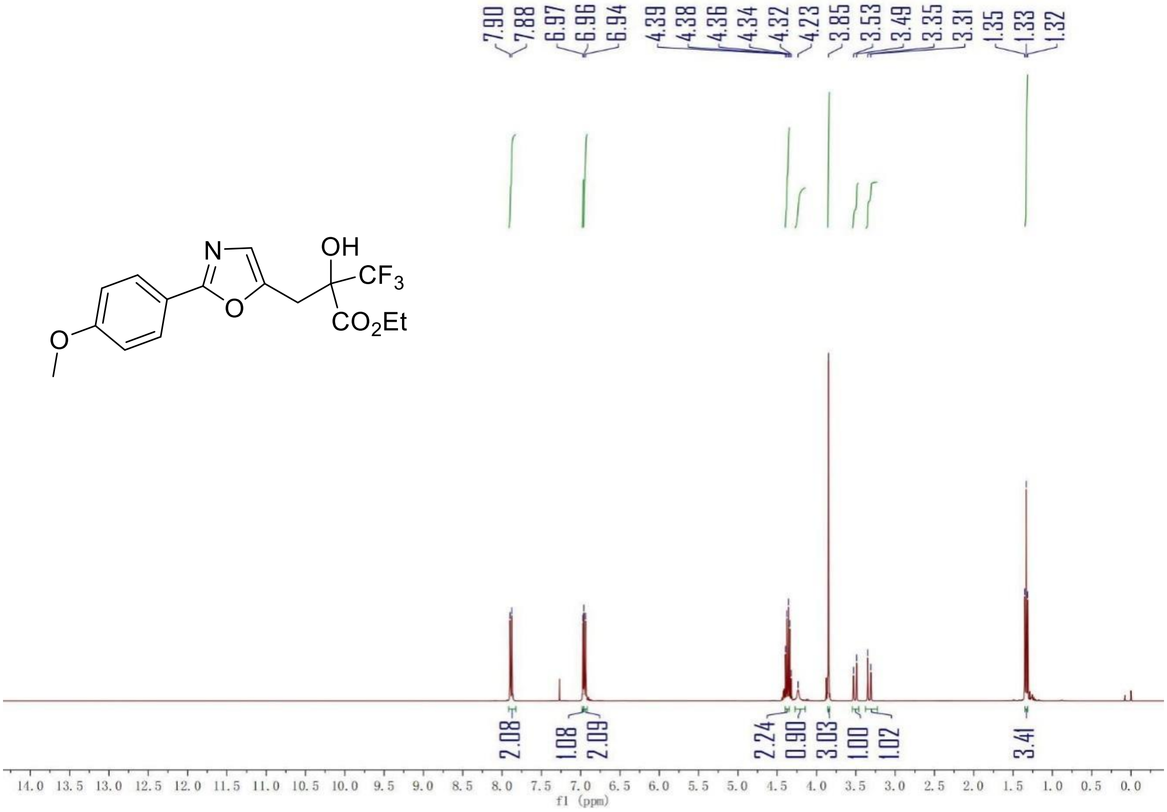

^{1}H NMR spectrum of compound **3d**

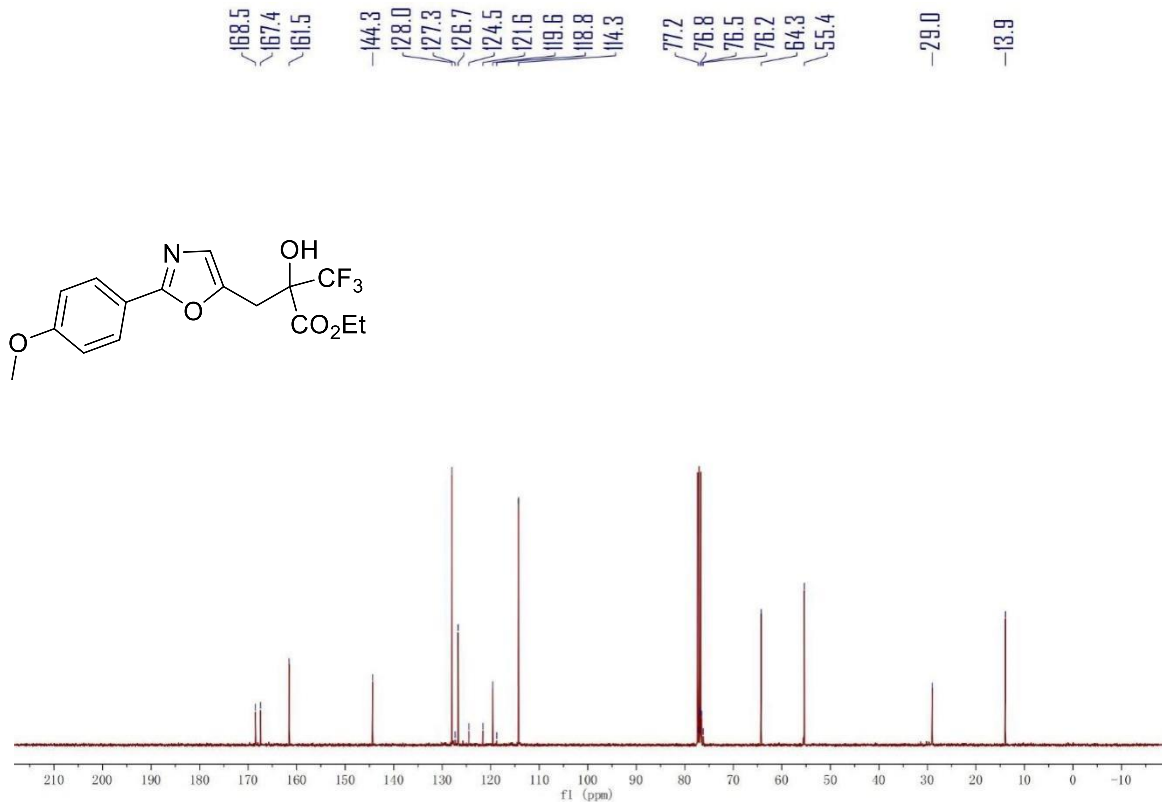

^{13}C NMR spectrum of compound **3d**

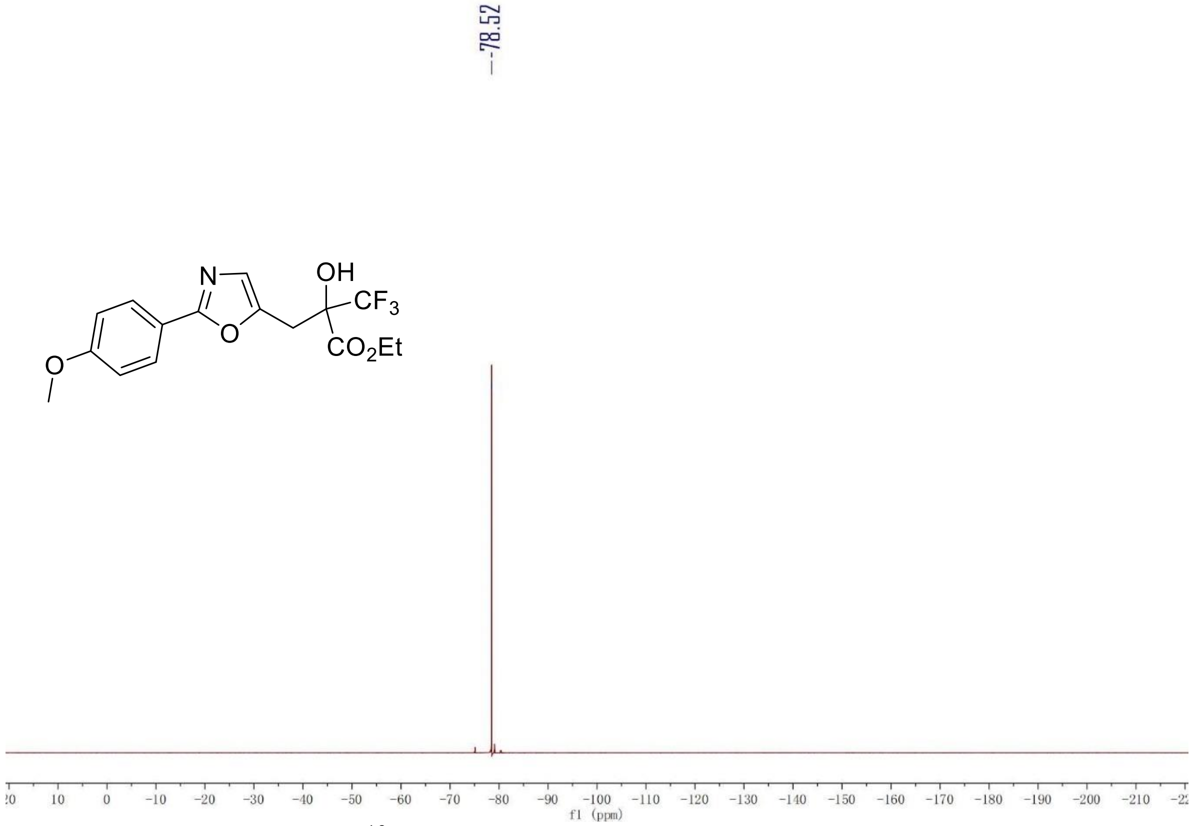

^{19}F NMR spectrum of compound **3d**

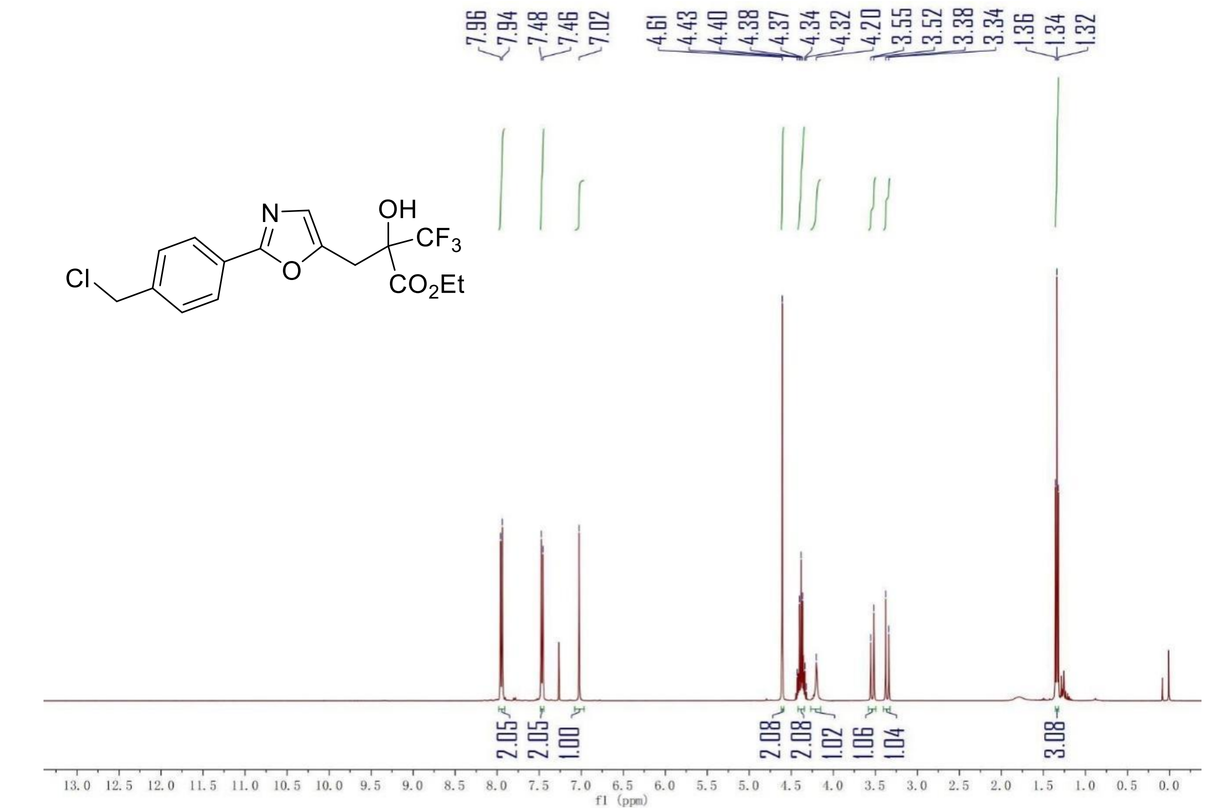

^{1}H NMR spectrum of compound **3e**

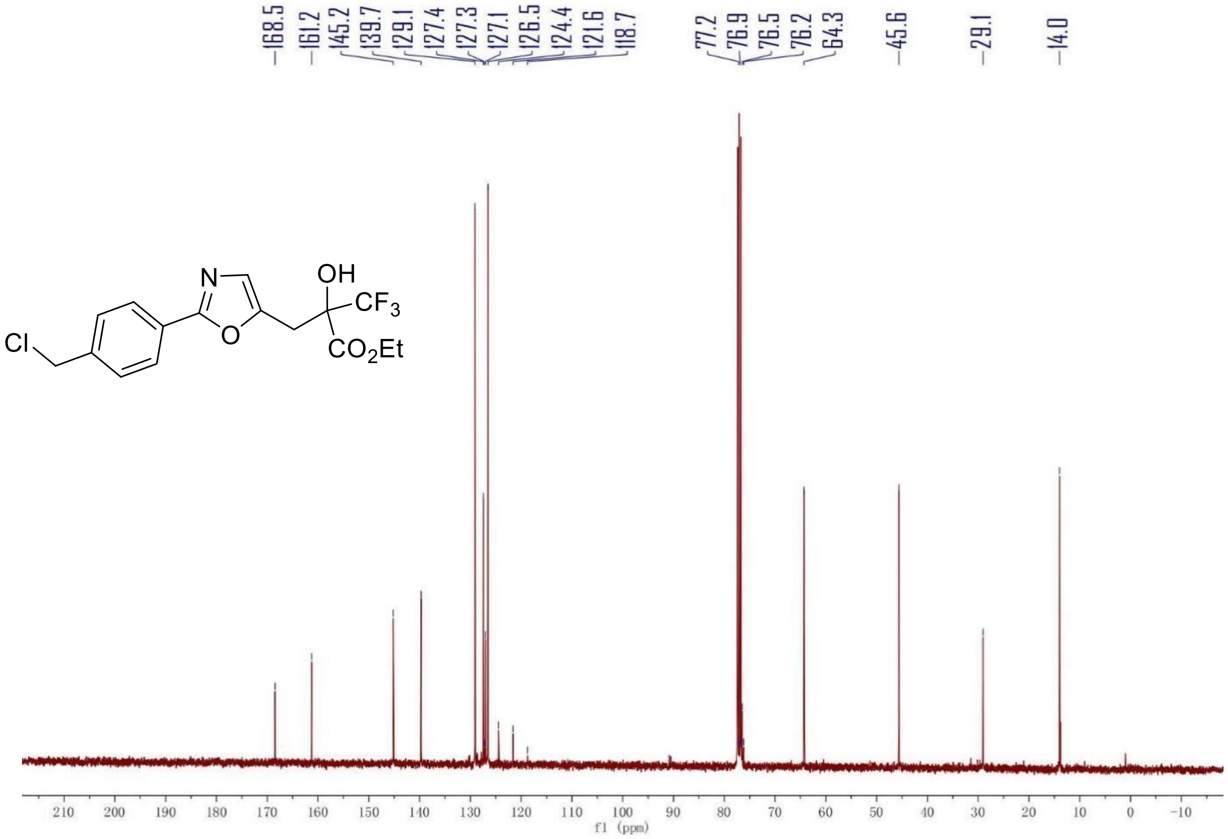

^{13}C NMR spectrum of compound **3e**

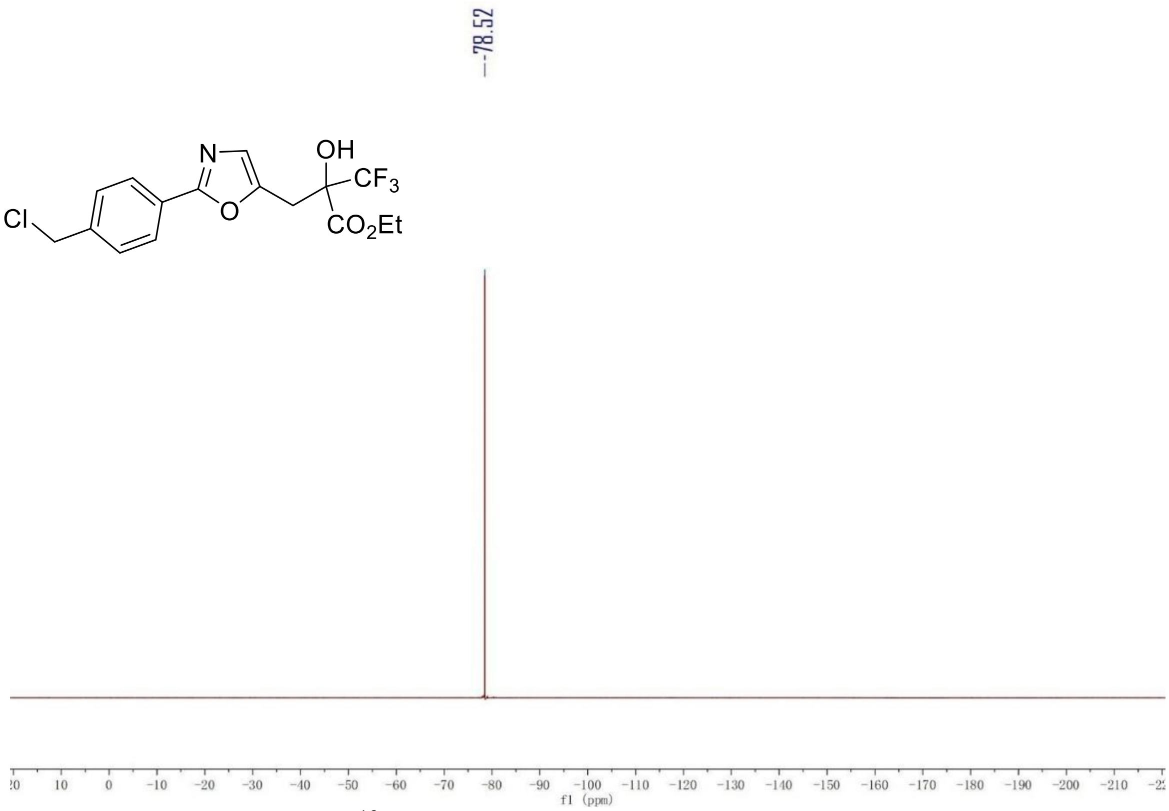

^{19}F NMR spectrum of compound **3e**

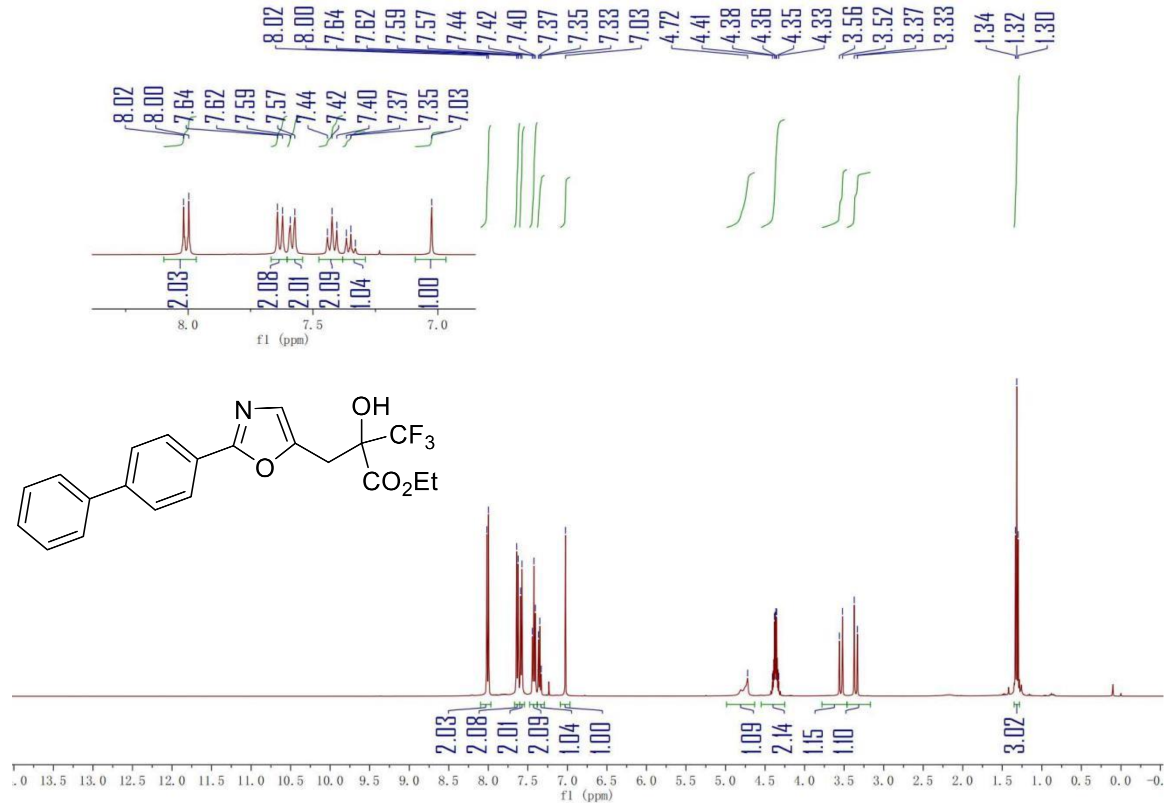

^{1}H NMR spectrum of compound **3f**

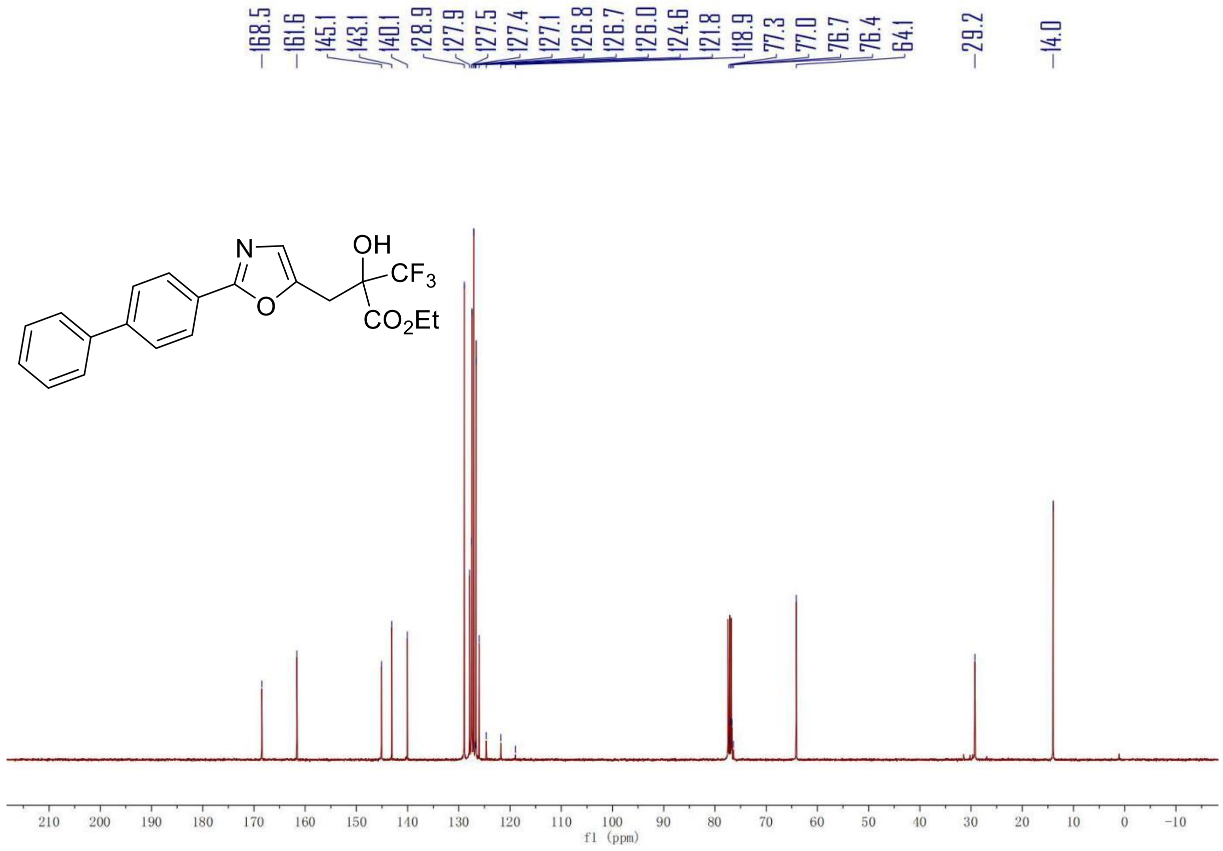

^{13}C NMR spectrum of compound **3f**

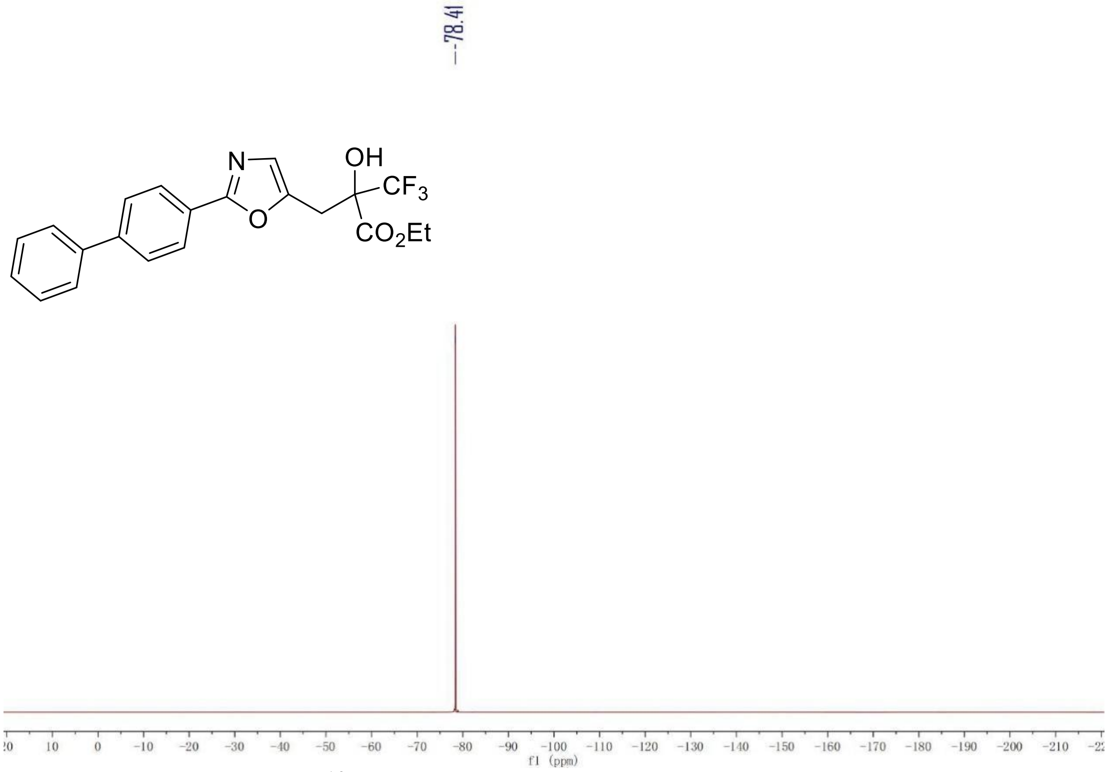

^{19}F NMR spectrum of compound **3f**

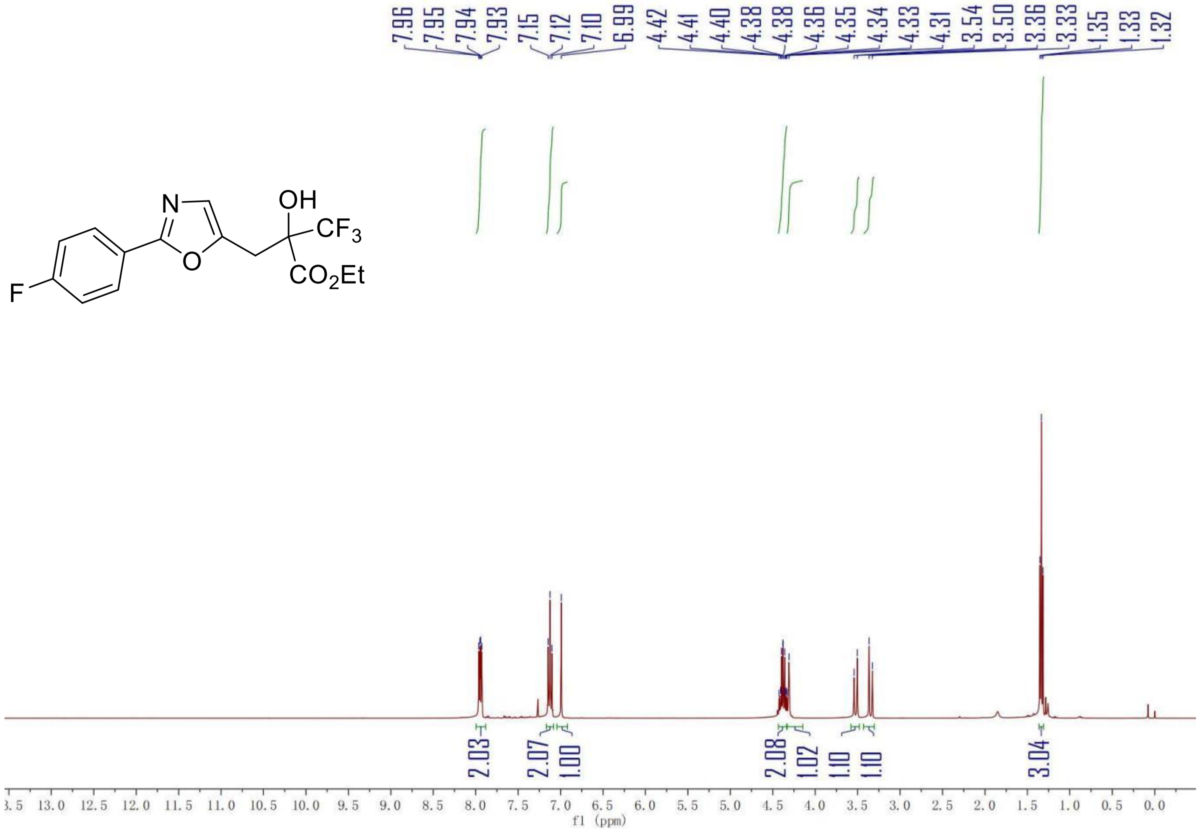

^{1}H NMR spectrum of compound **3g**

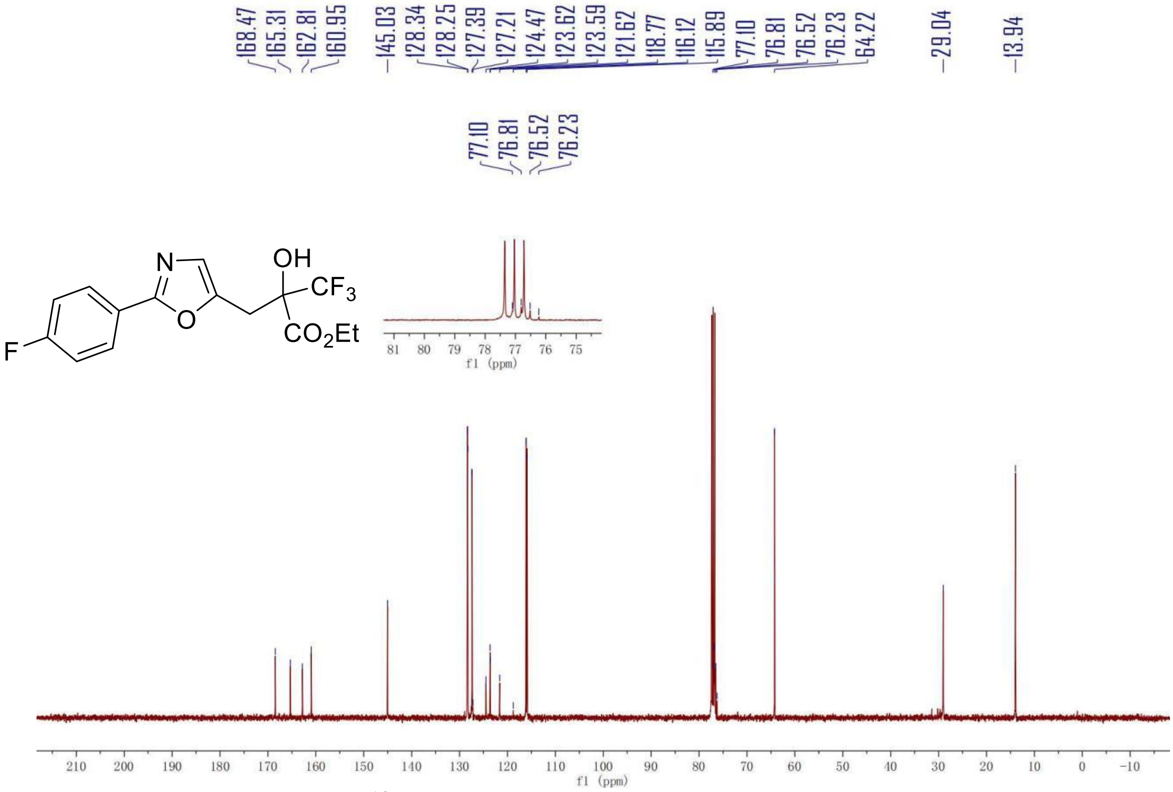

^{13}C NMR spectrum of compound **3g**

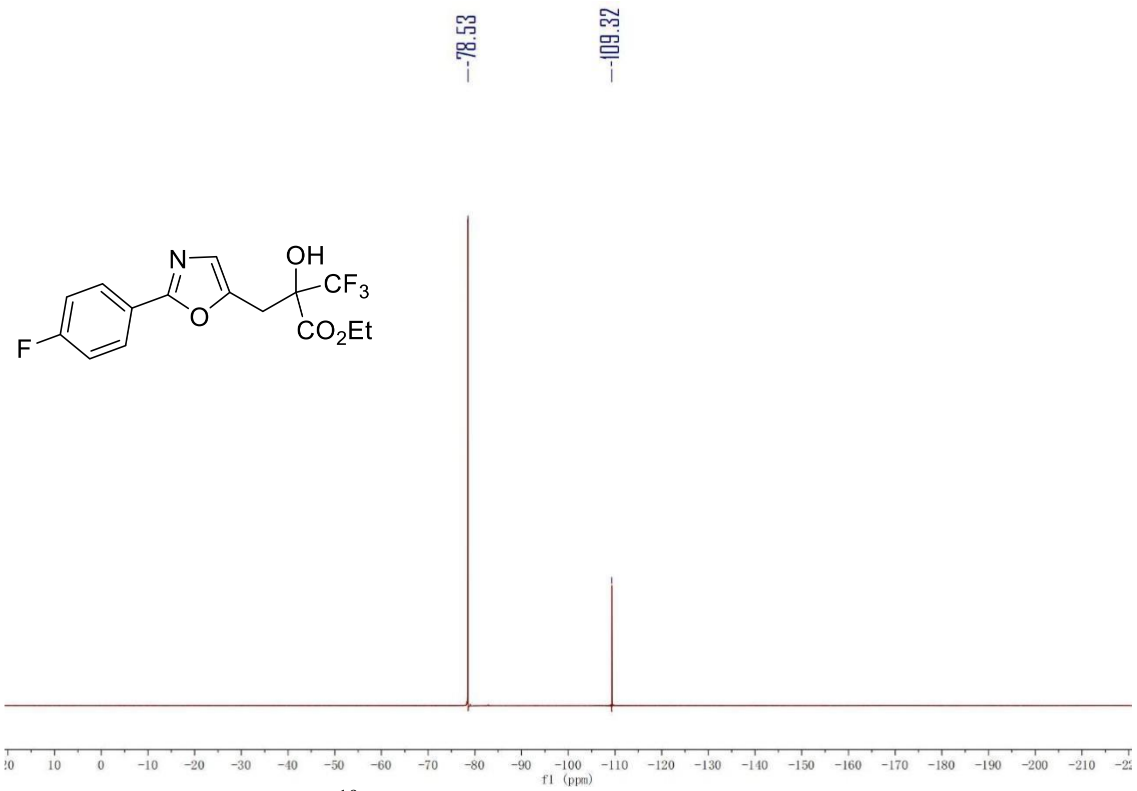

^{19}F NMR spectrum of compound **3g**

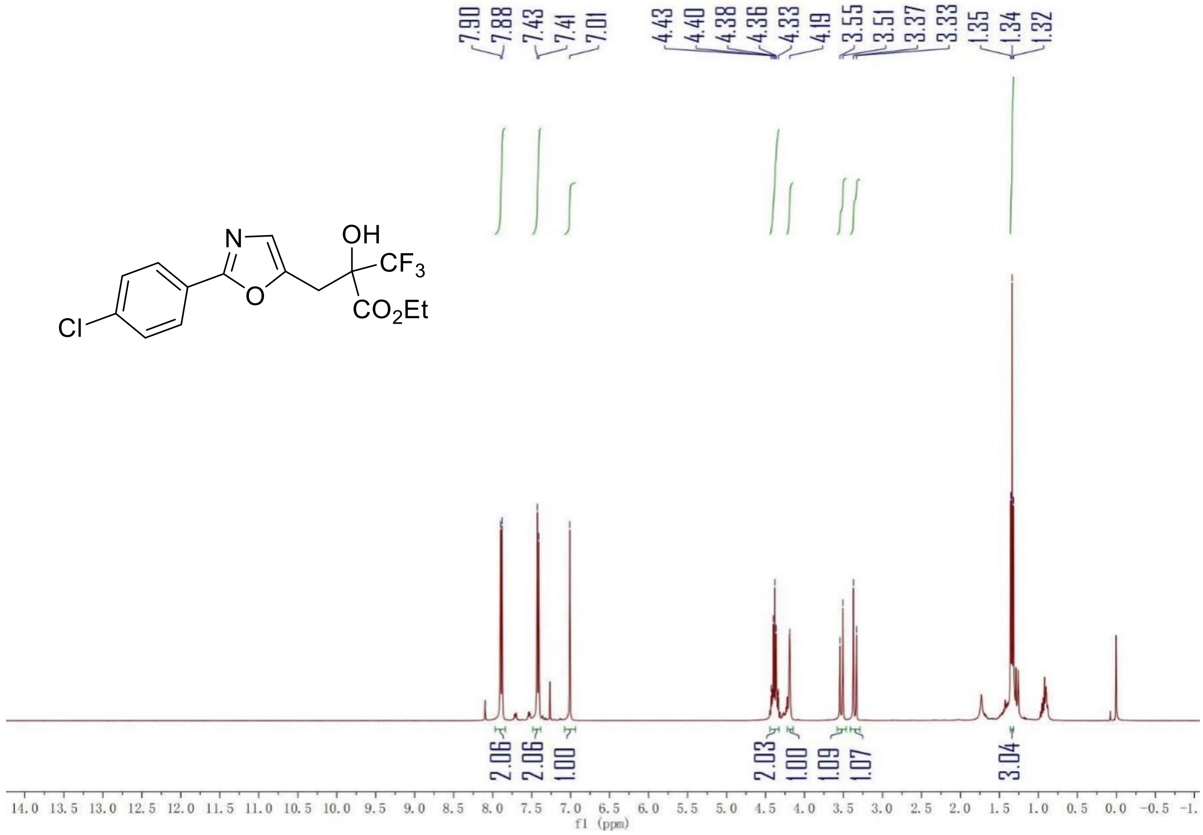

^{1}H NMR spectrum of compound **3h**

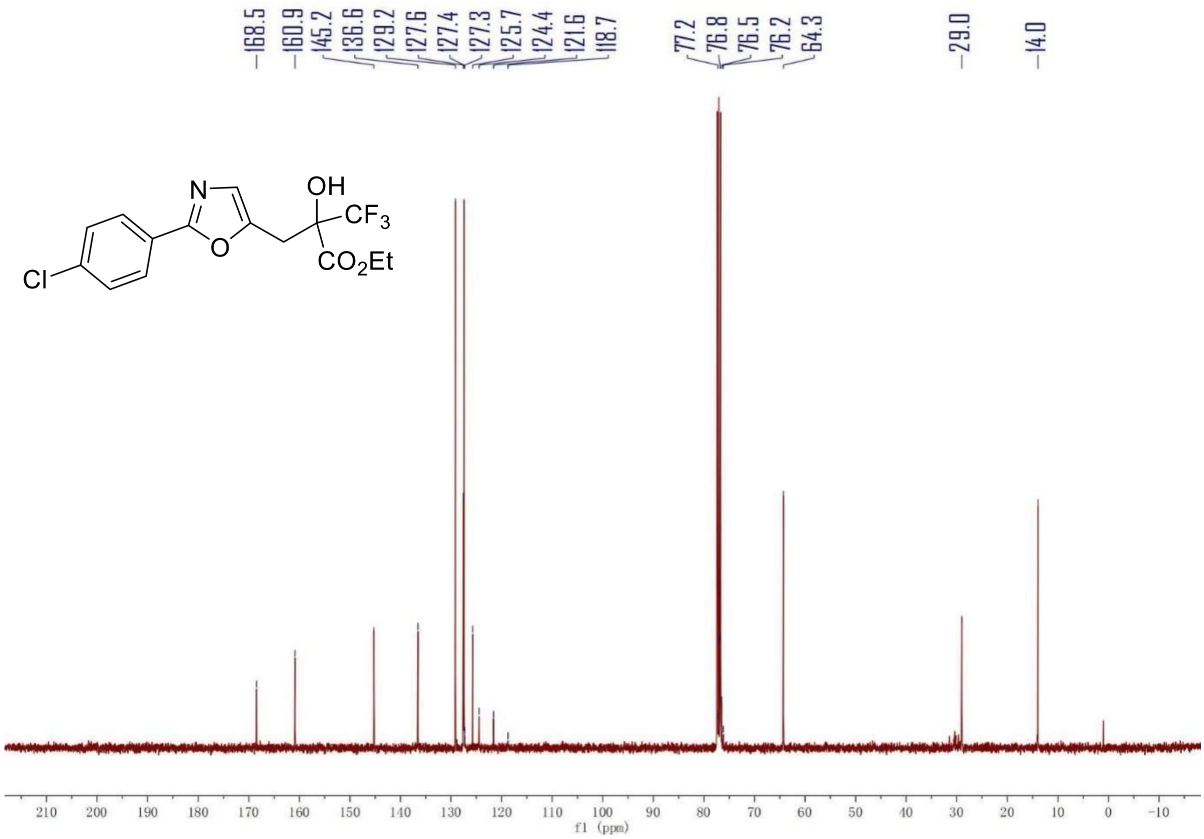

^{13}C NMR spectrum of compound **3h**

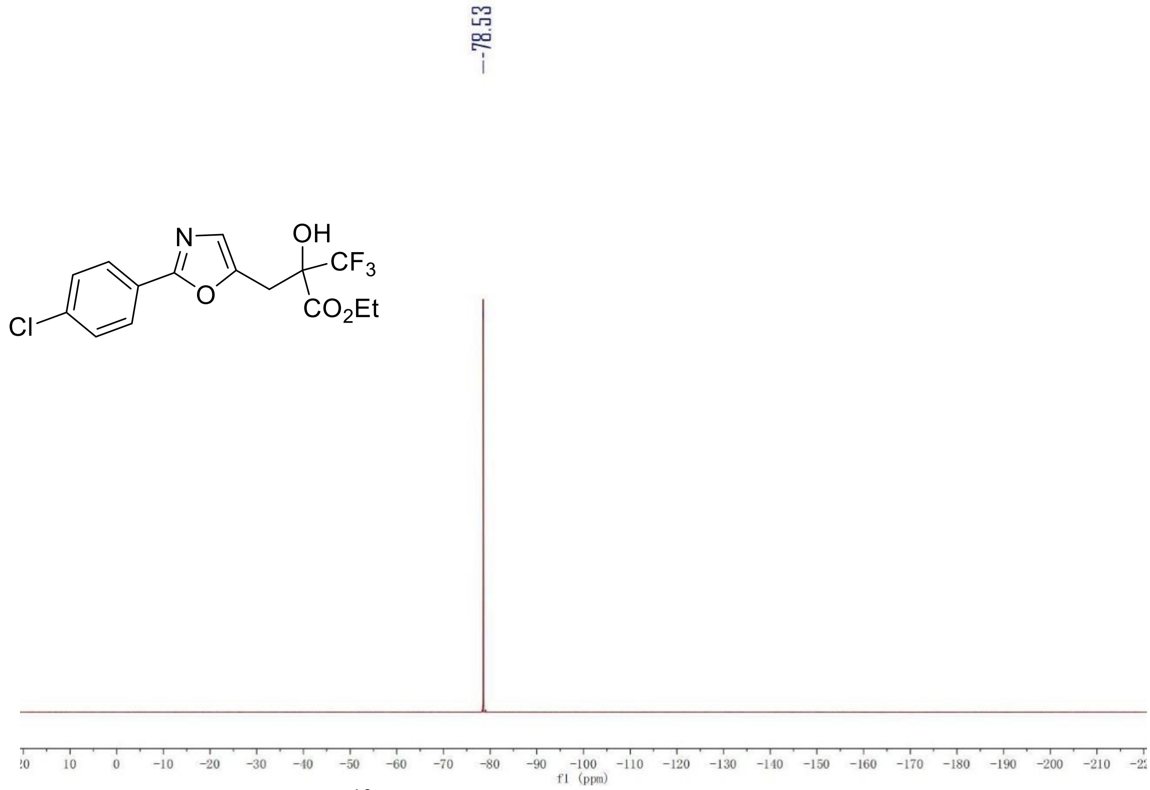

^{19}F NMR spectrum of compound **3h**

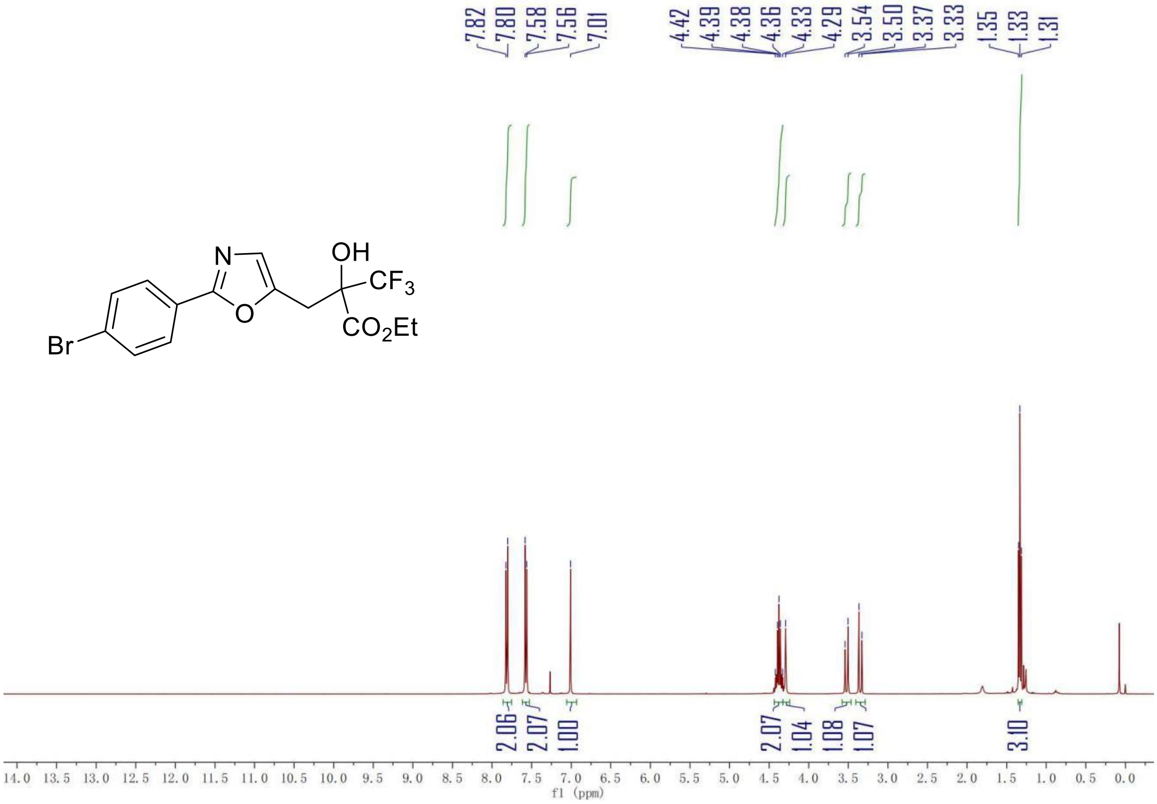

^{1}H NMR spectrum of compound **3i**

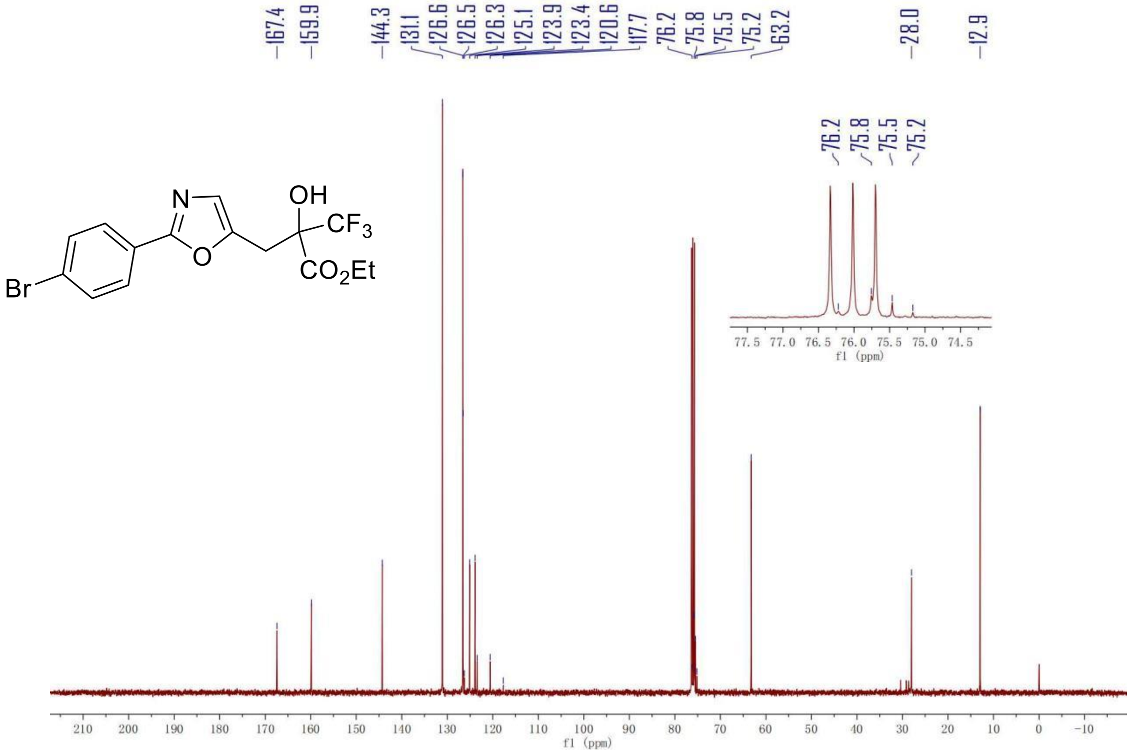

^{13}C NMR spectrum of compound **3i**

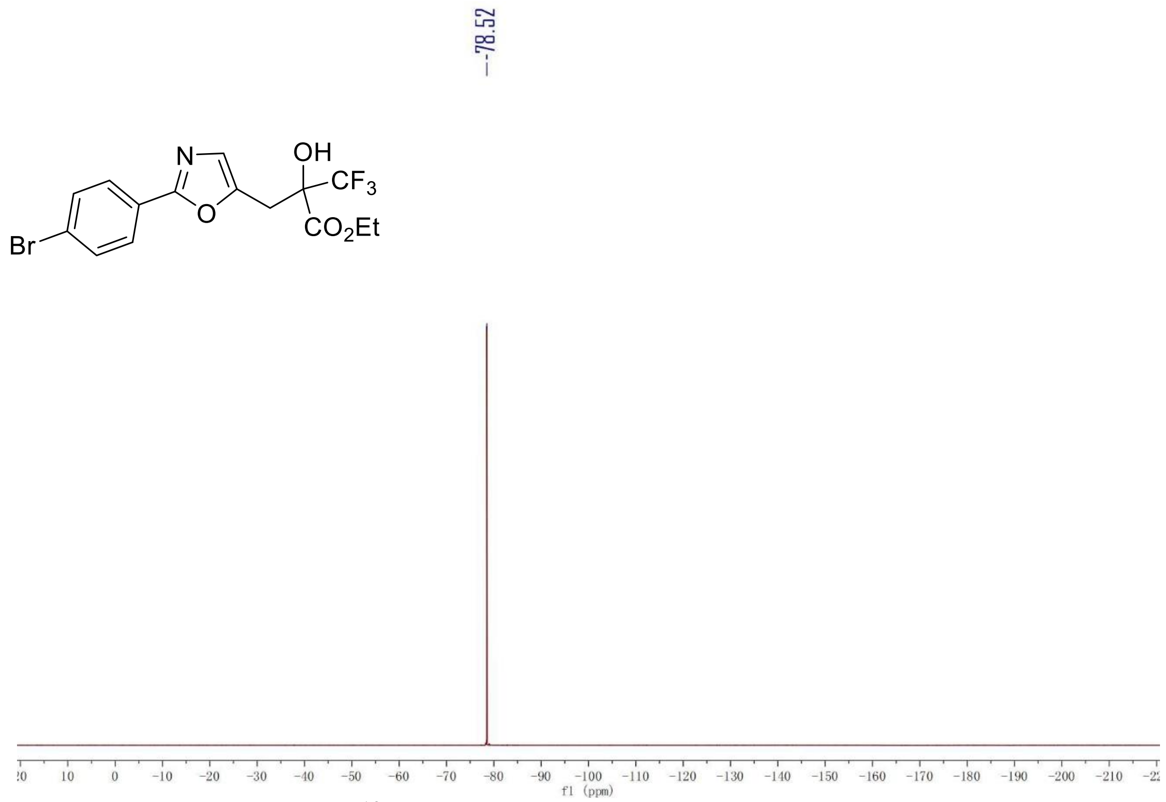

^{19}F NMR spectrum of compound **3i**

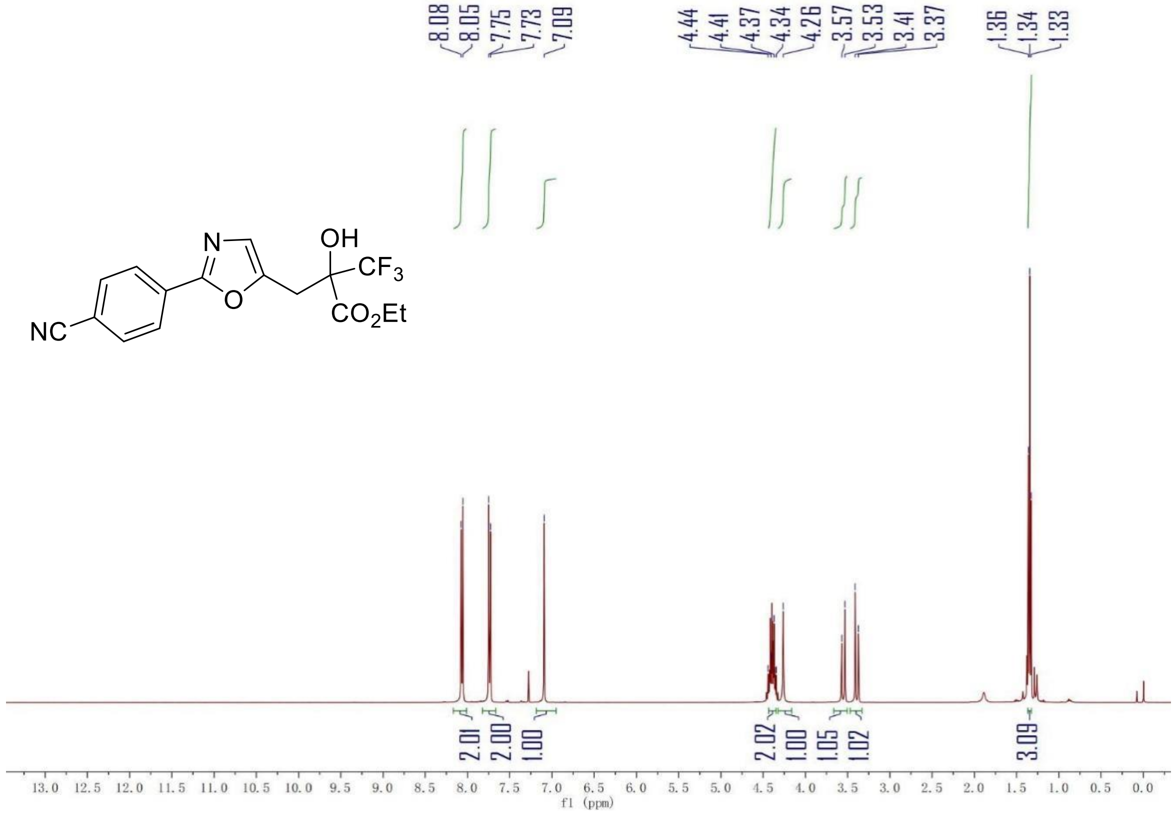

^{1}H NMR spectrum of compound **3j**

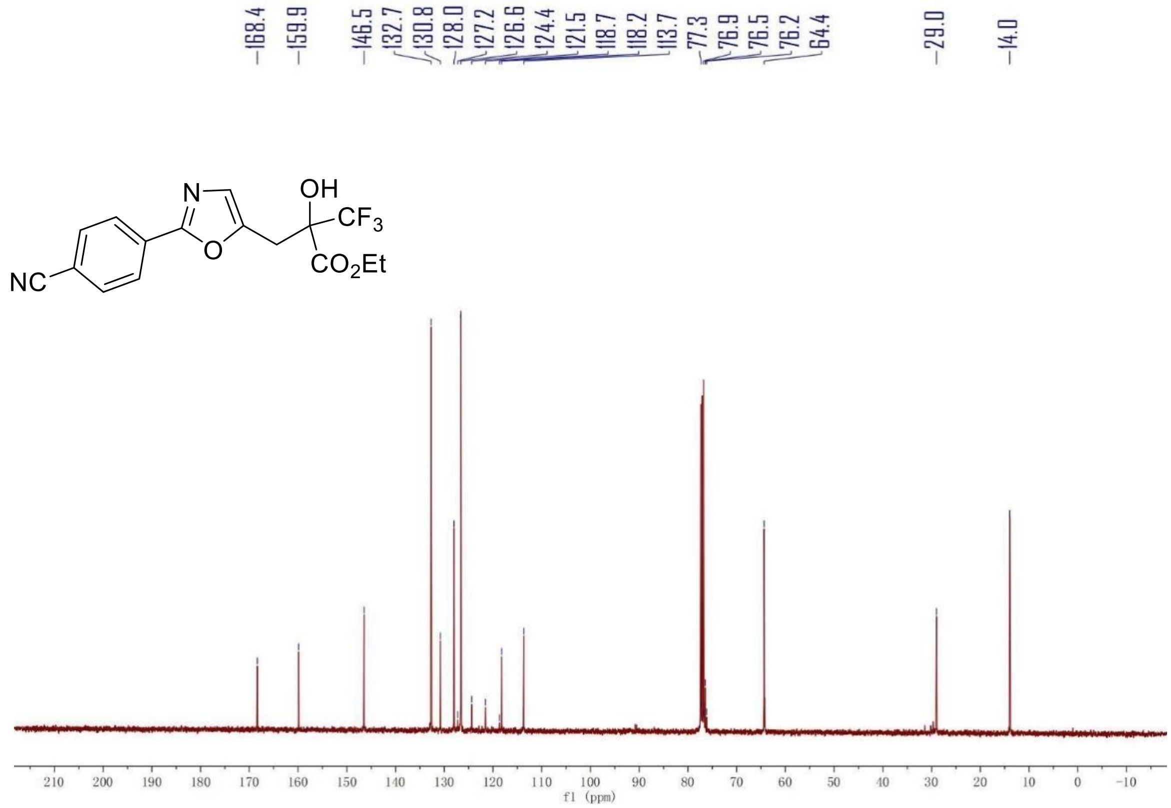

^{13}C NMR spectrum of compound **3j**

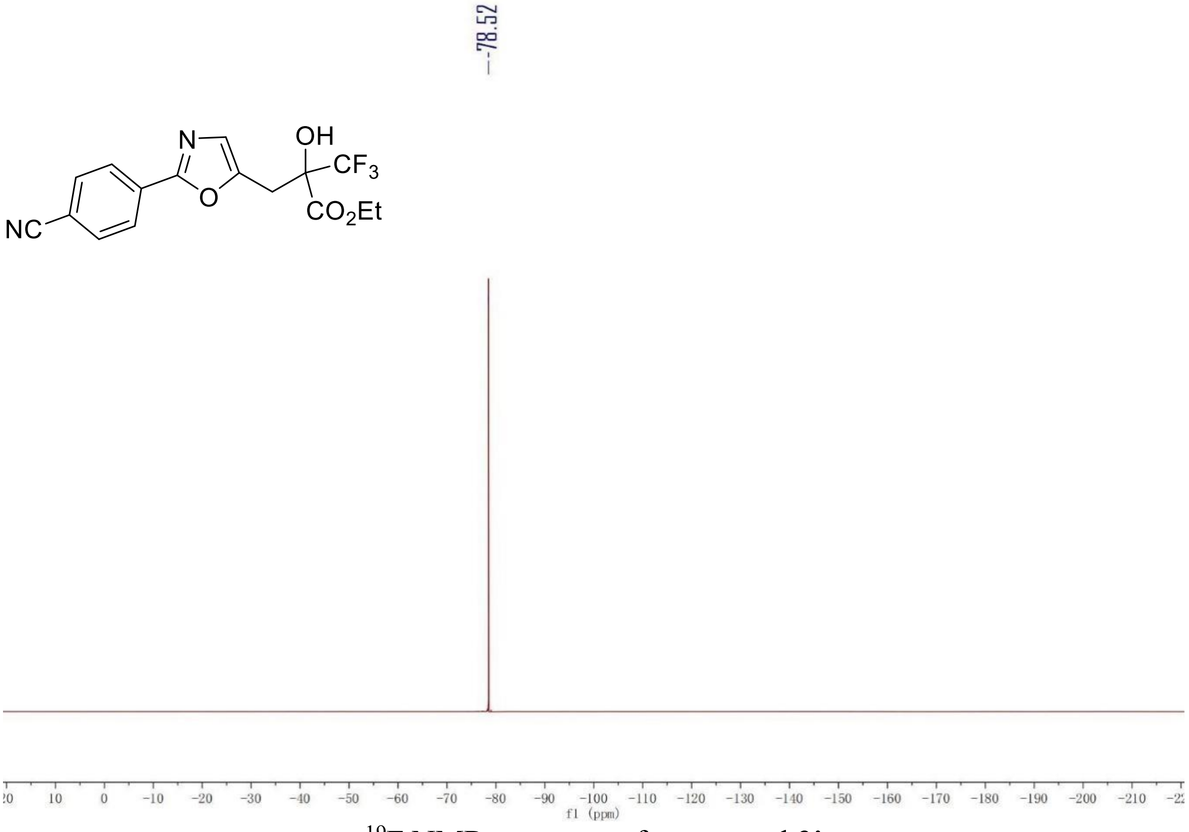

^{19}F NMR spectrum of compound **3j**

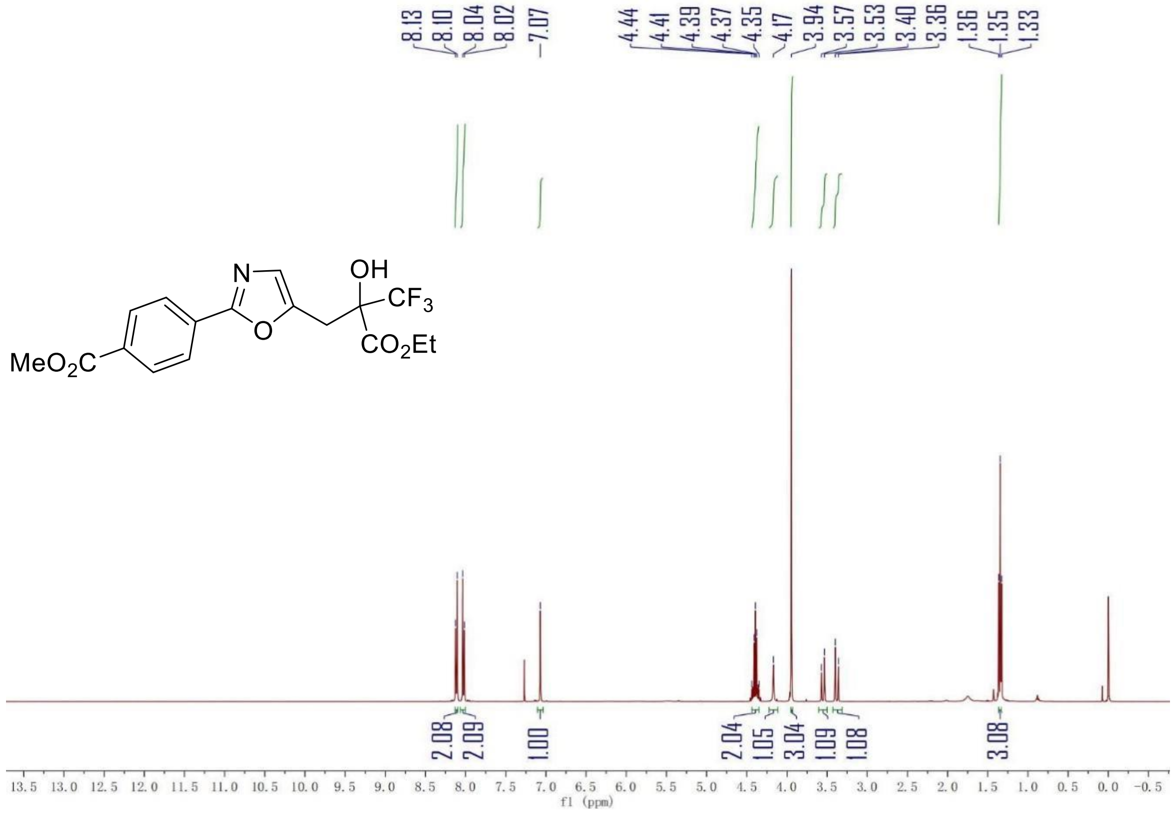

^{1}H NMR spectrum of compound **3k**

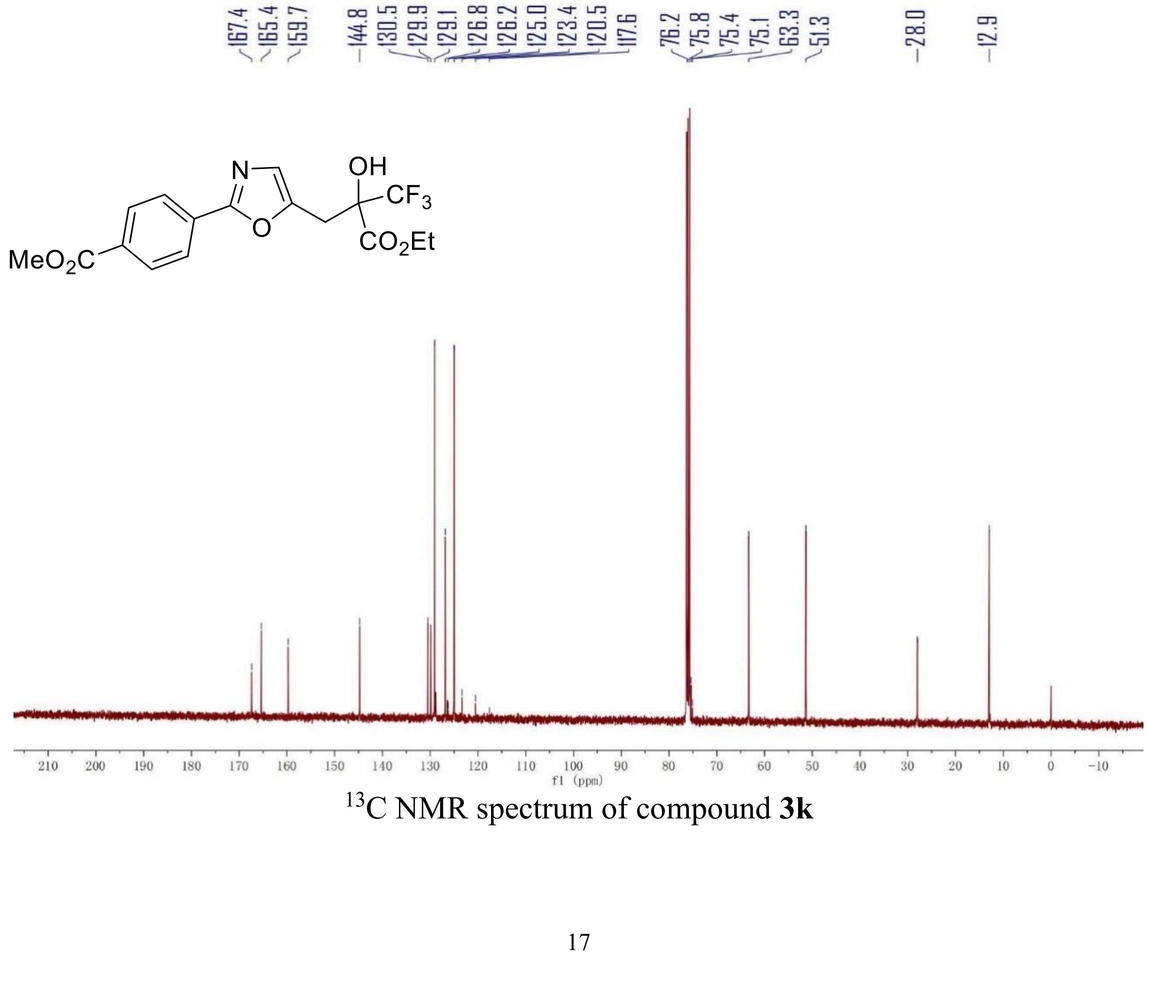

^{13}C NMR spectrum of compound **3k**

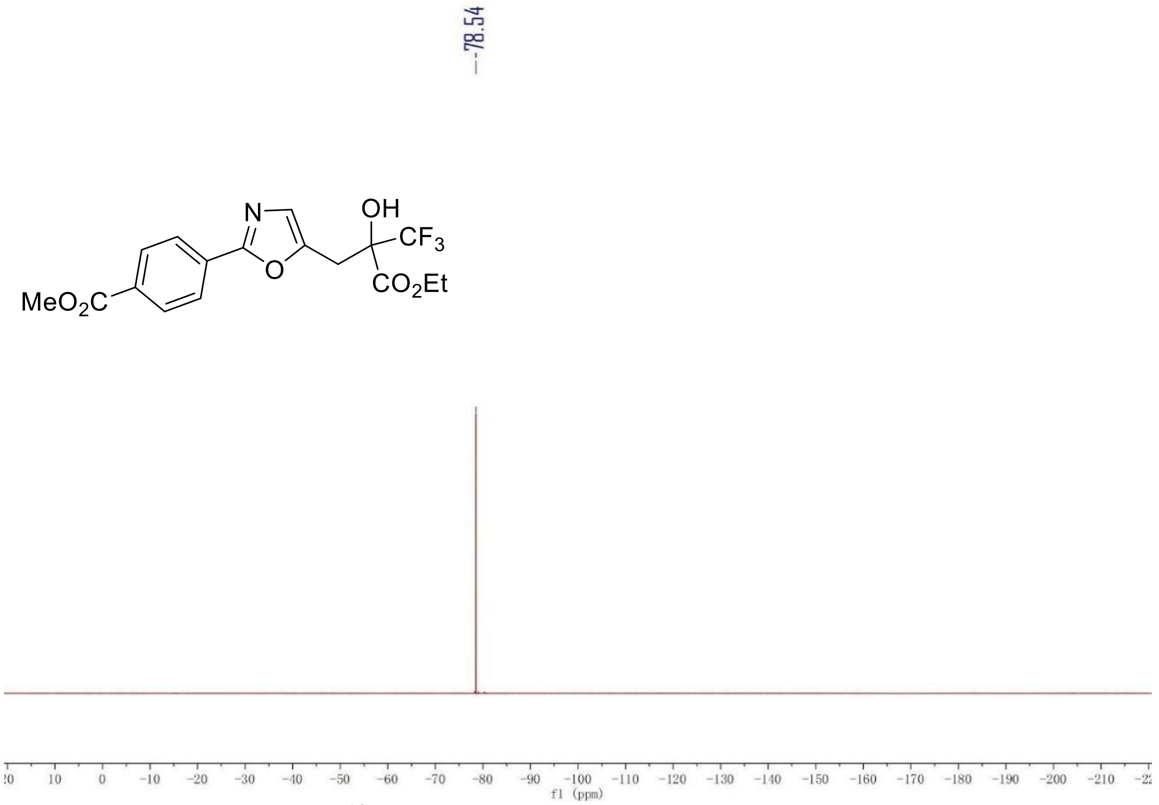

^{19}F NMR spectrum of compound **3k**

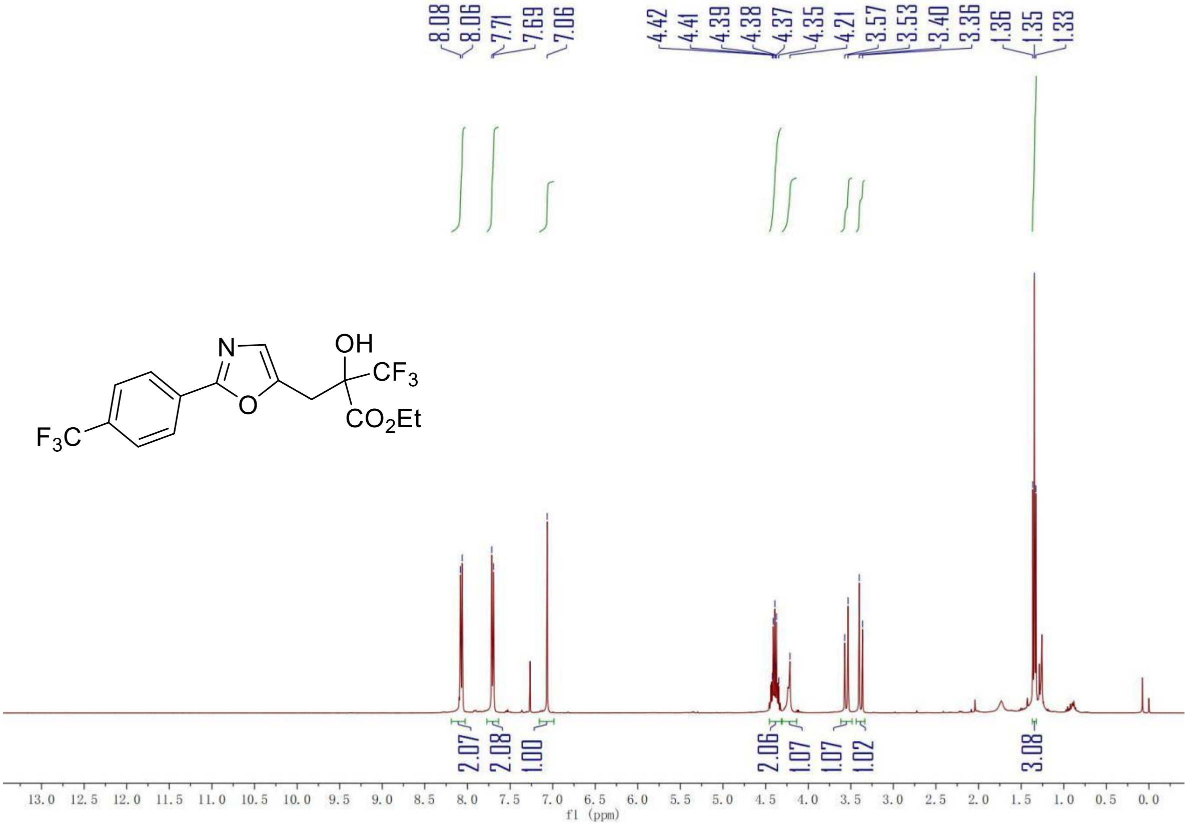

^{1}H NMR spectrum of compound **3l**

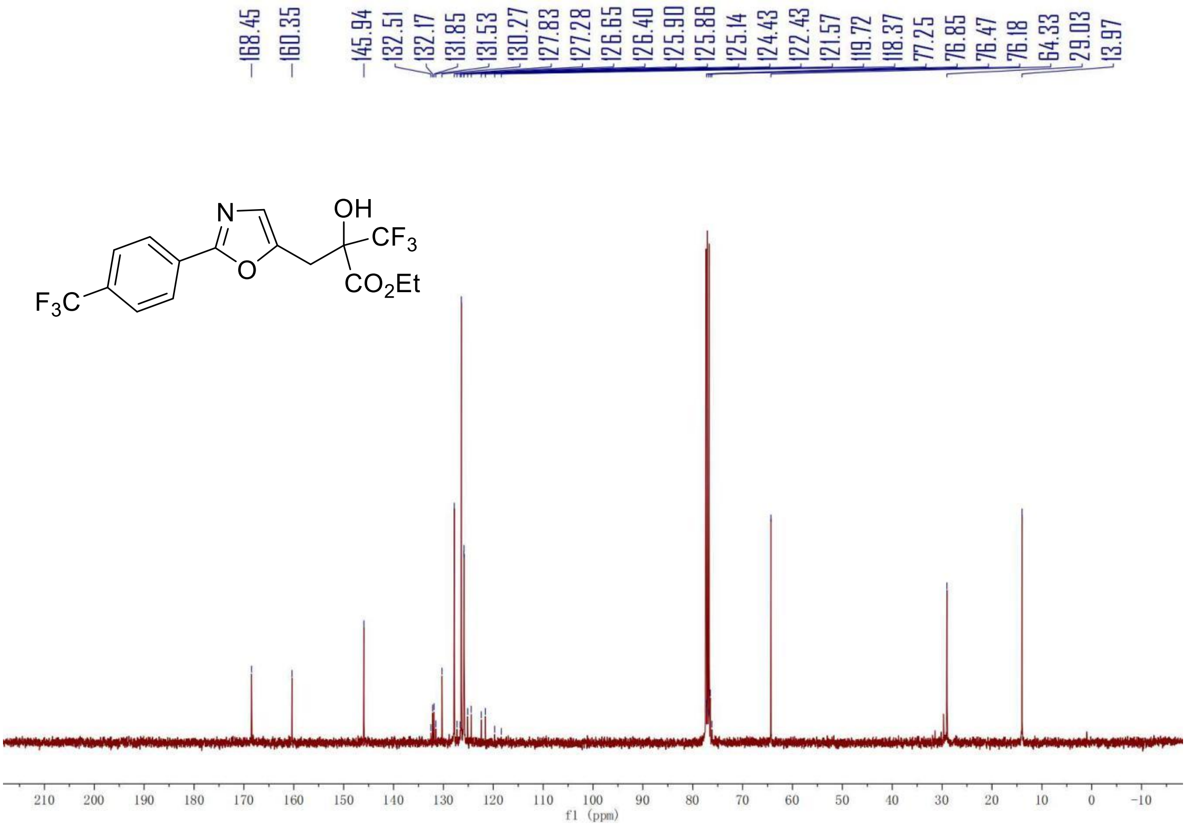

^{13}C NMR spectrum of compound **3l**

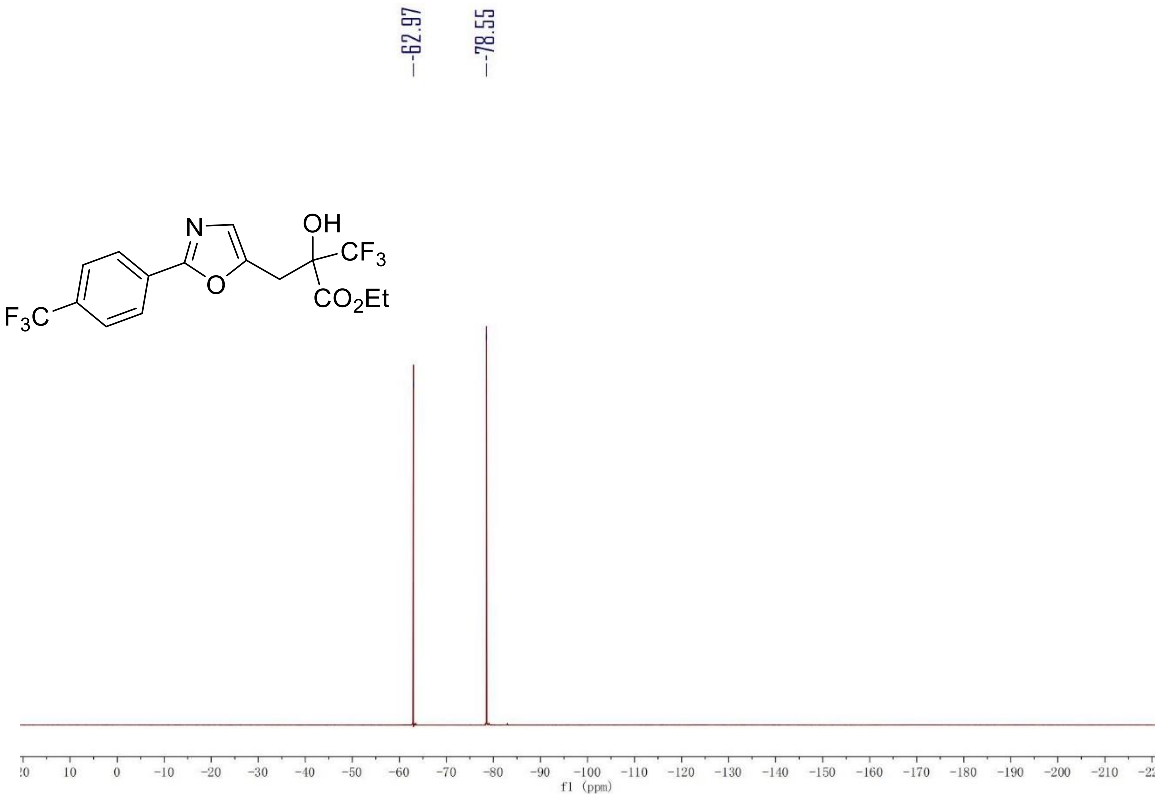

^{19}F NMR spectrum of compound **3l**

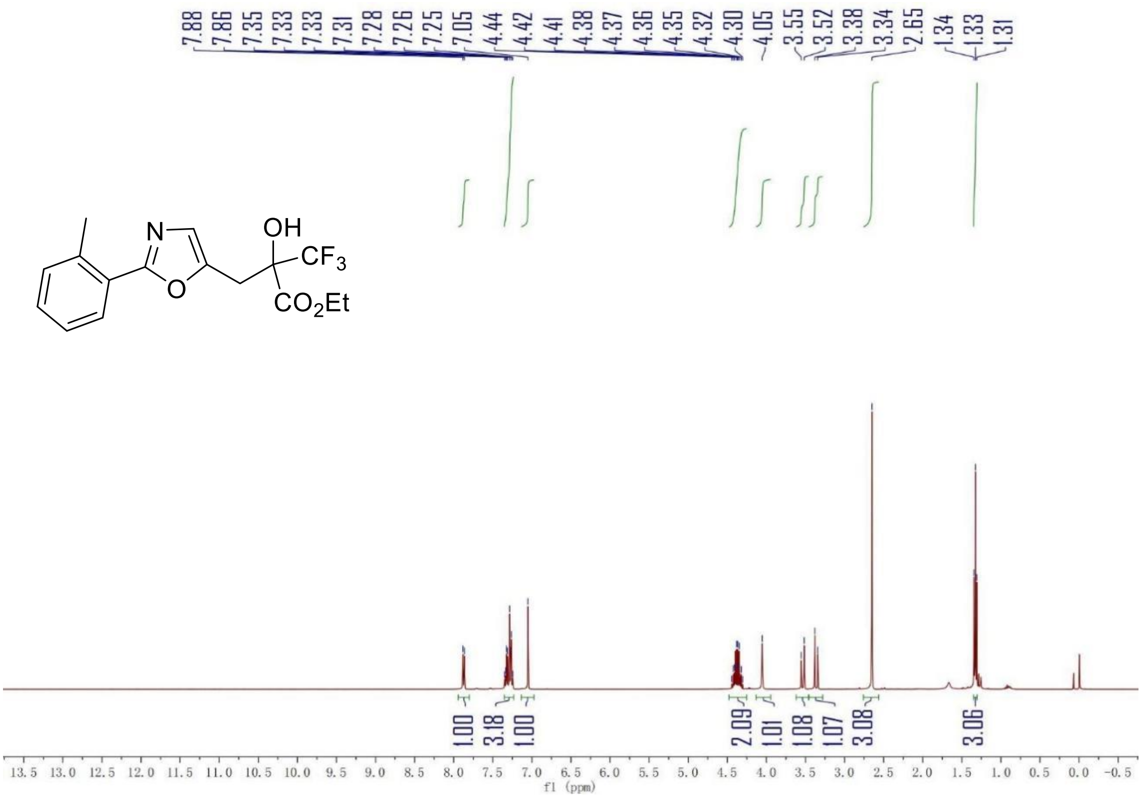

^{1}H NMR spectrum of compound **3m**

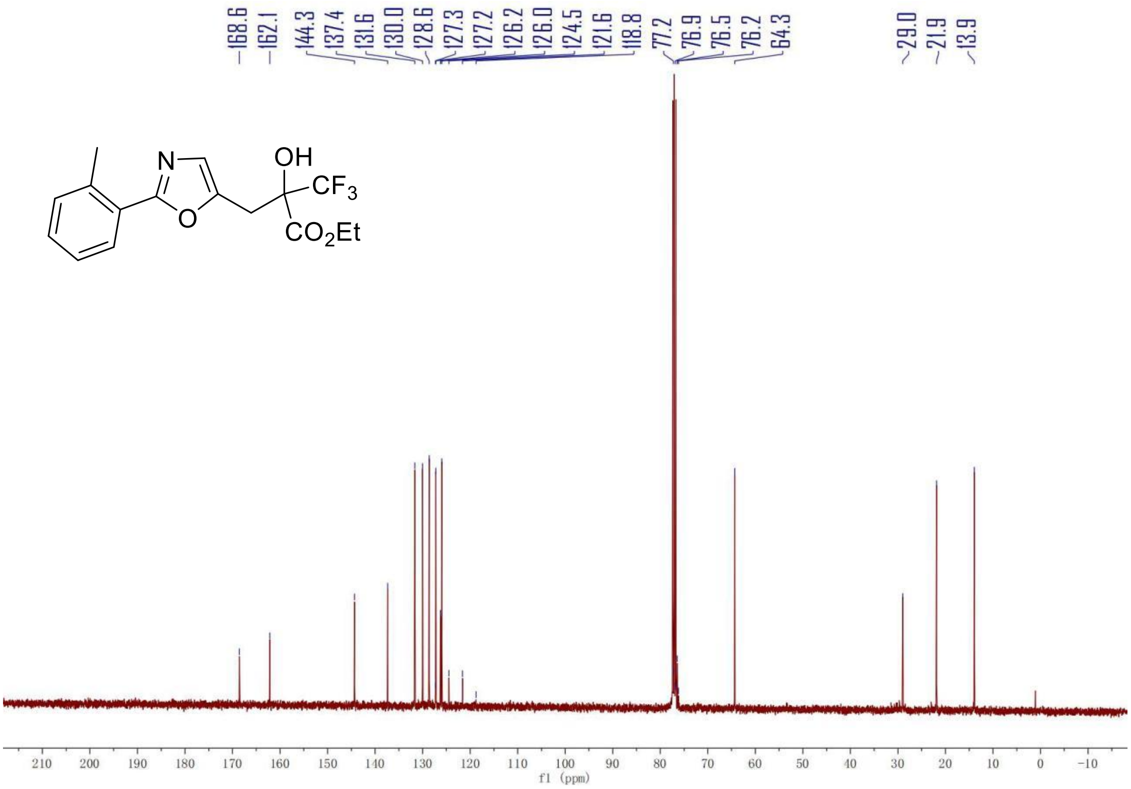

^{13}C NMR spectrum of compound **3m**

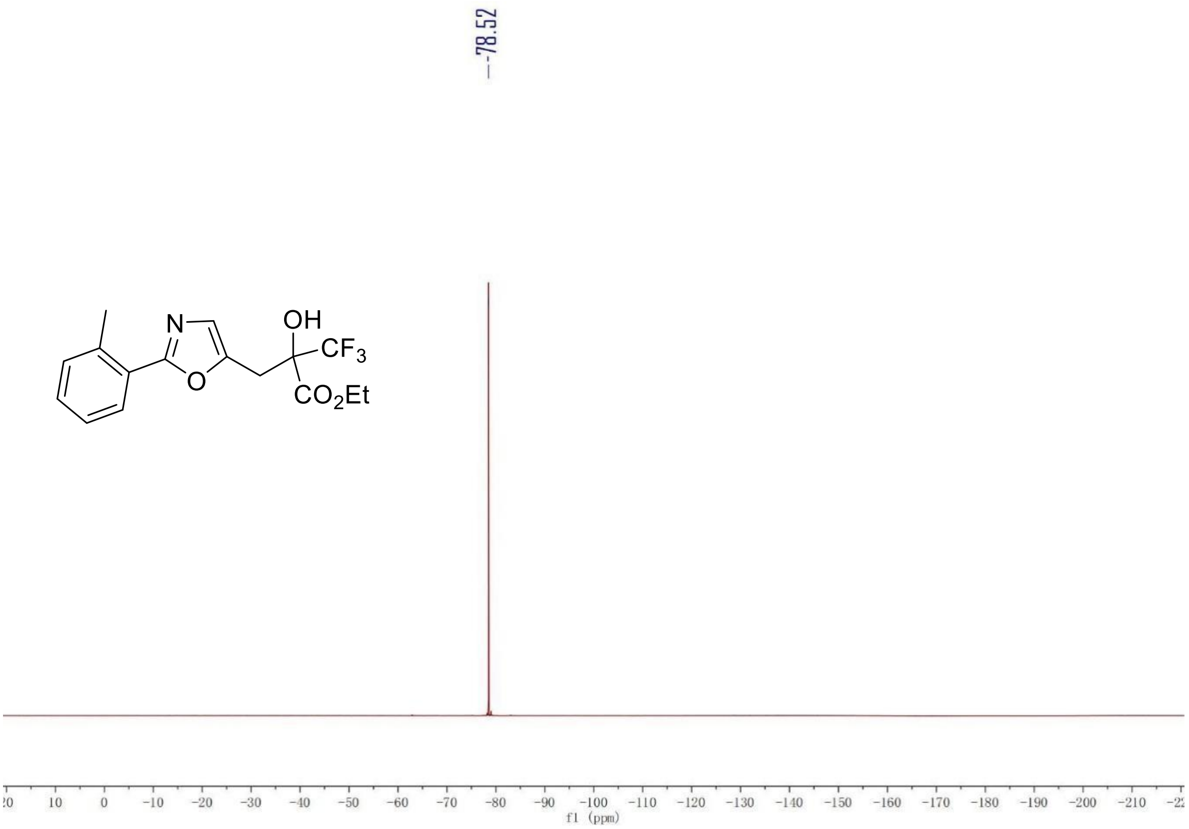

^{19}F NMR spectrum of compound **3m**

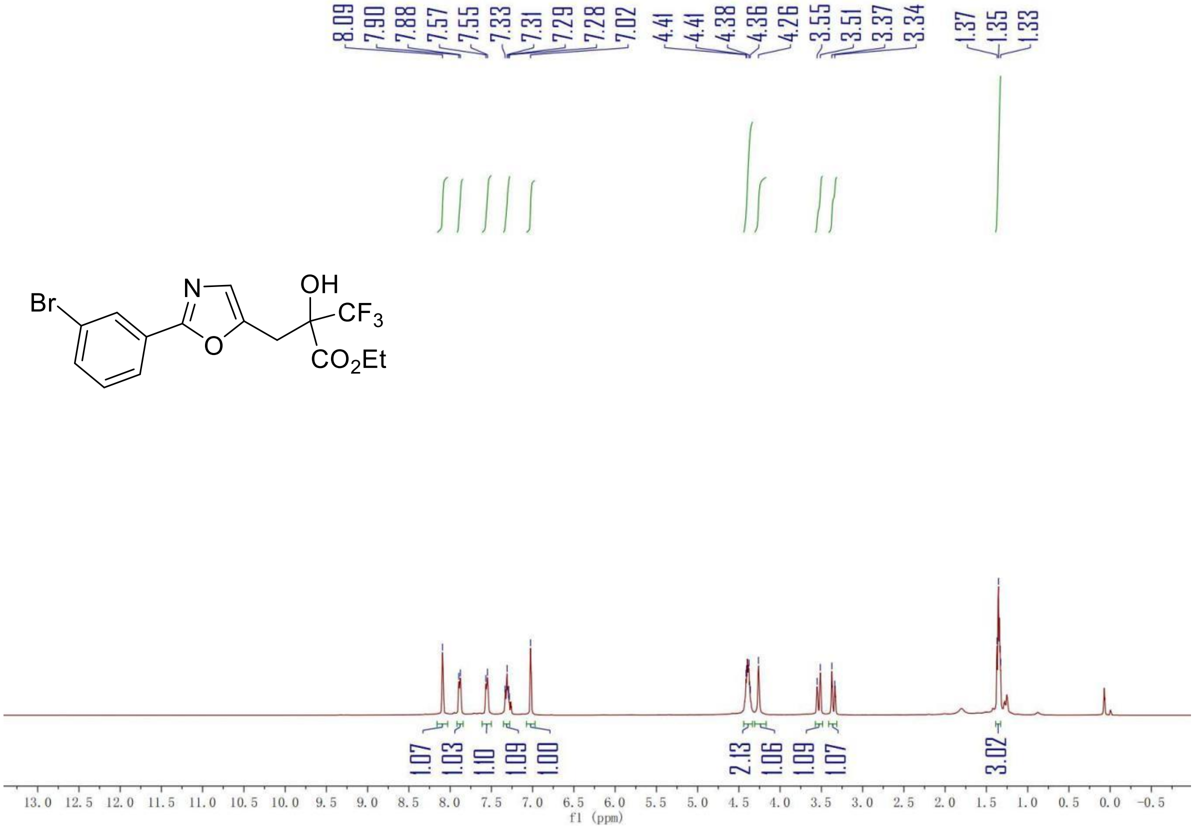

^{1}H NMR spectrum of compound **3n**

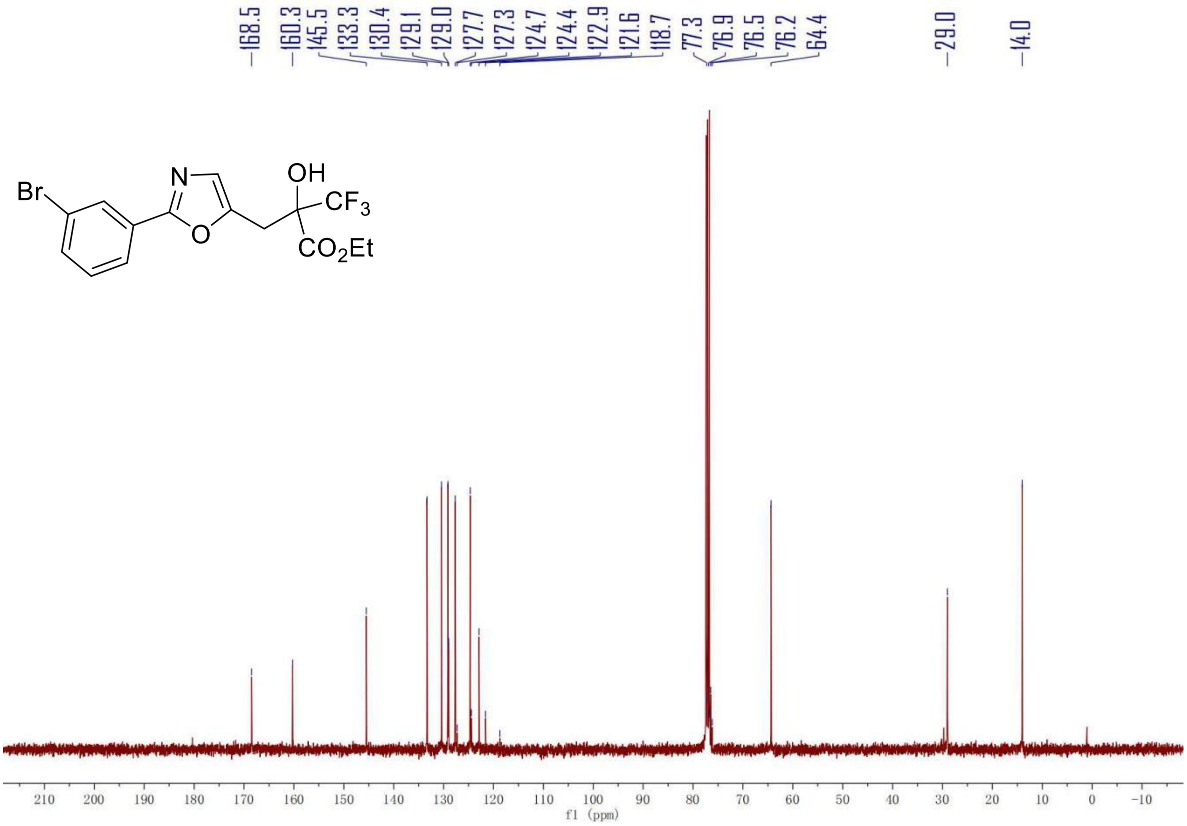

^{13}C NMR spectrum of compound **3n**

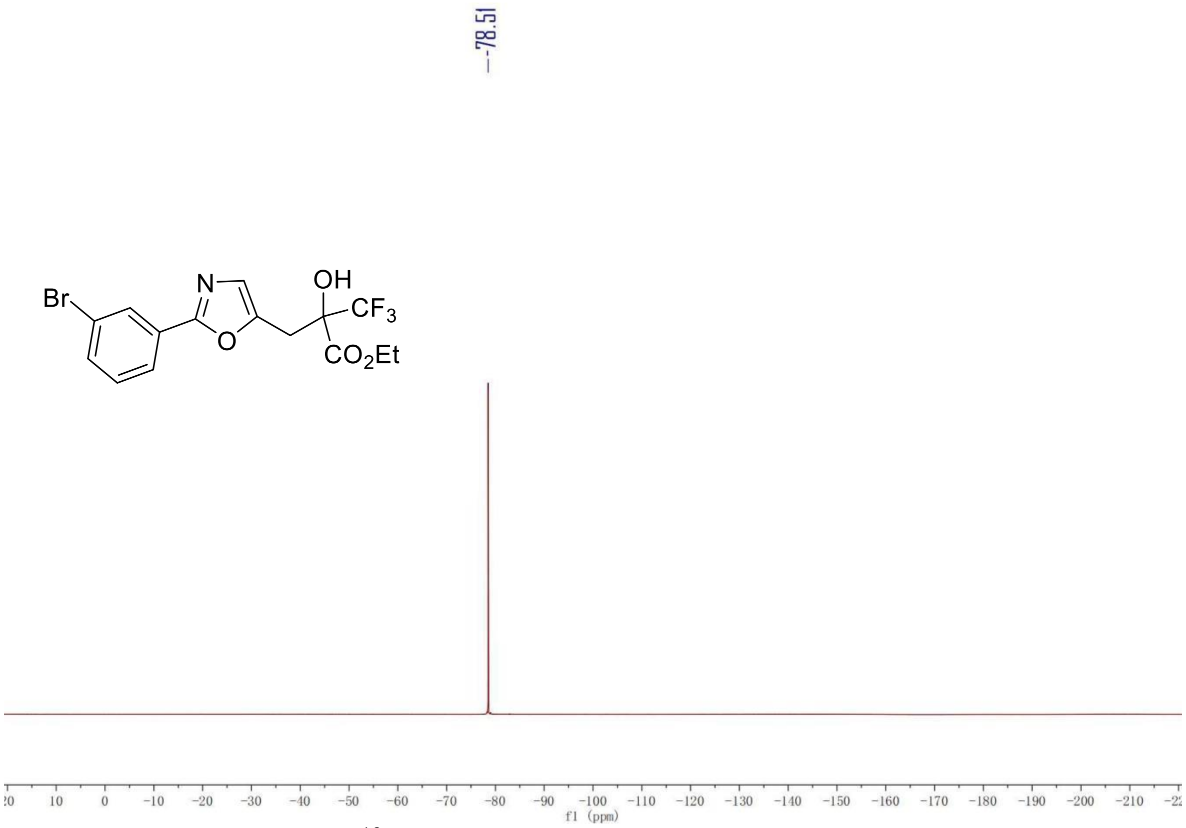

^{19}F NMR spectrum of compound **3n**

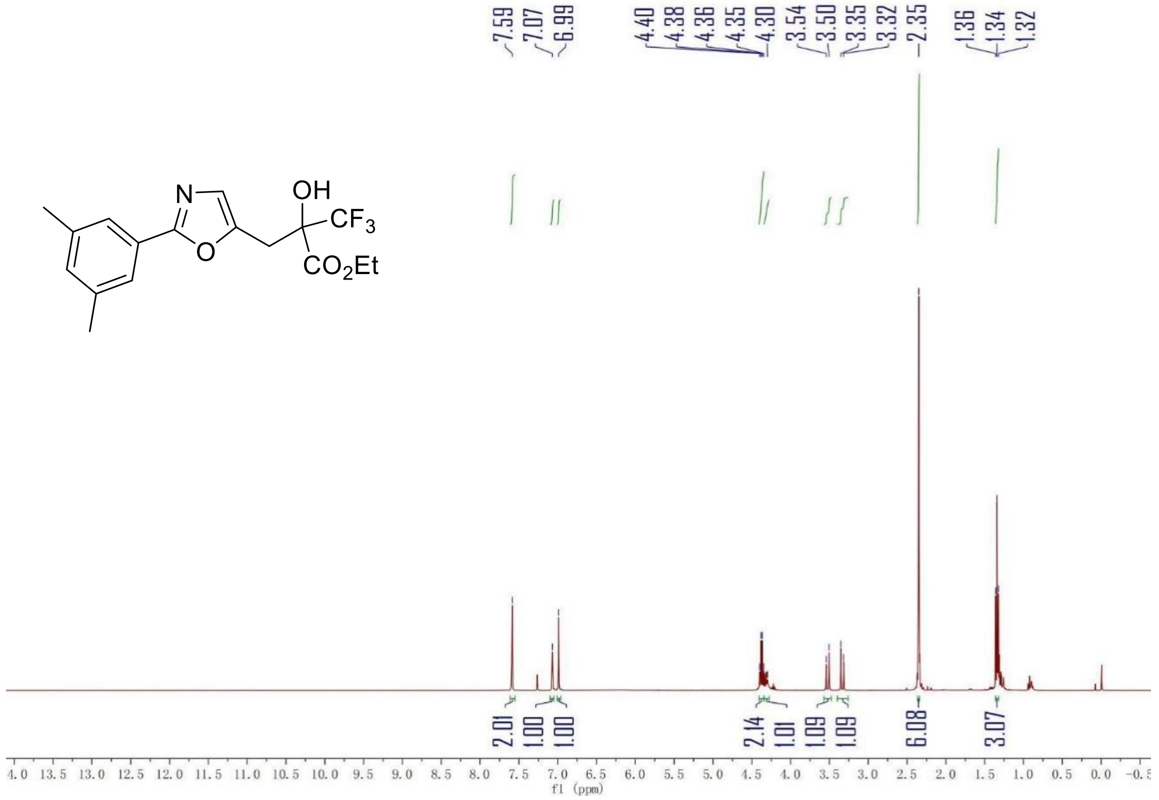

^{1}H NMR spectrum of compound **3o**

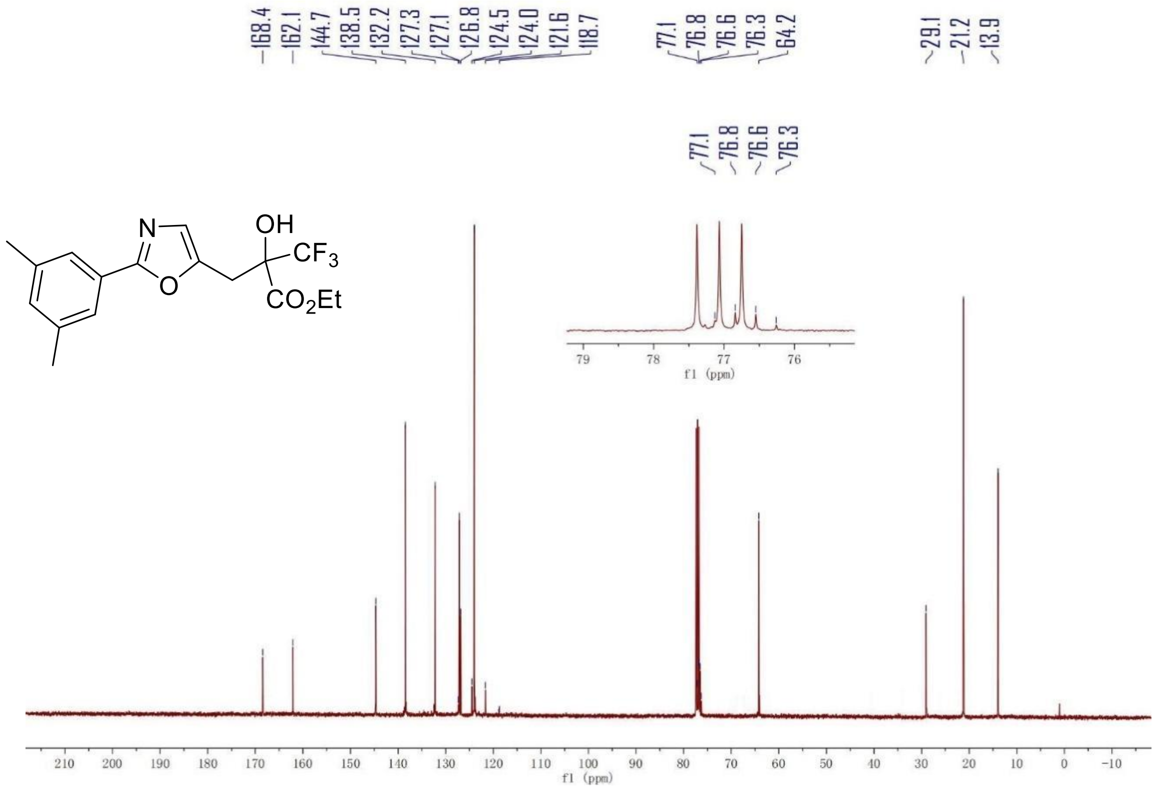

^{13}C NMR spectrum of compound **3o**

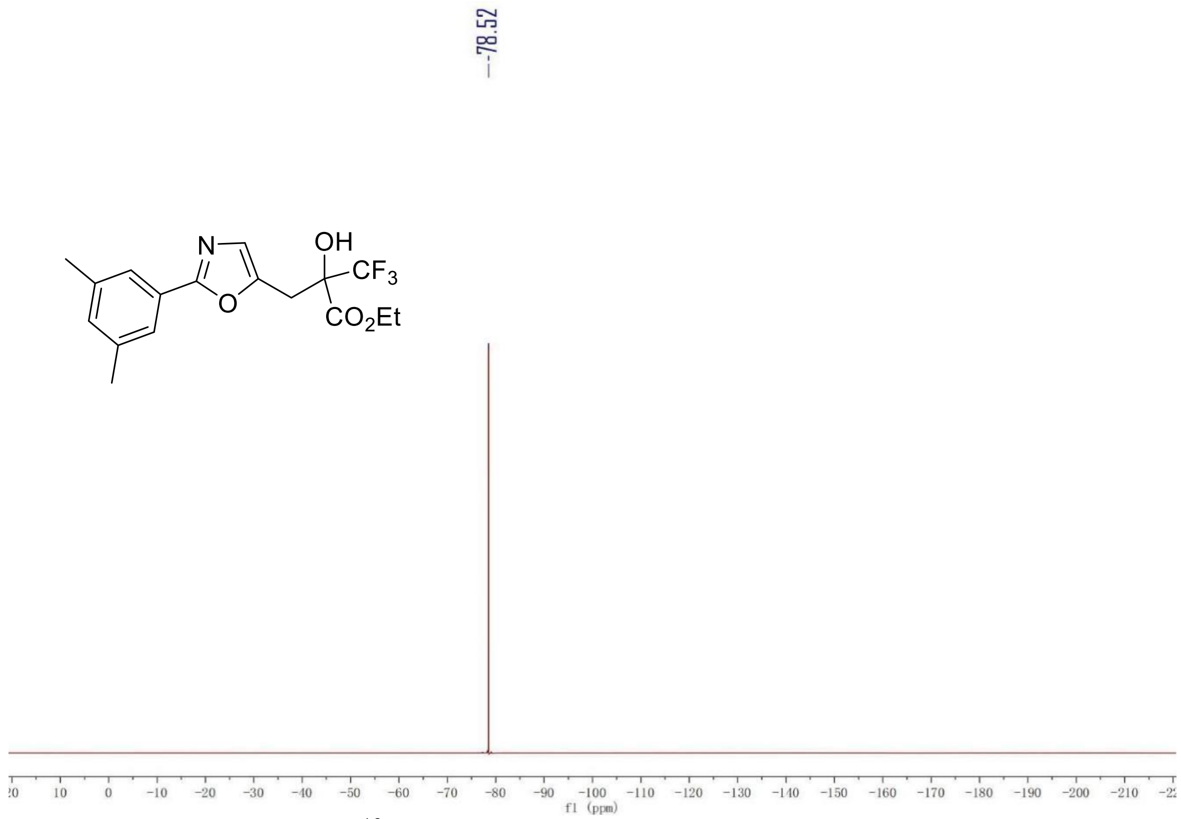

^{19}F NMR spectrum of compound **3o**

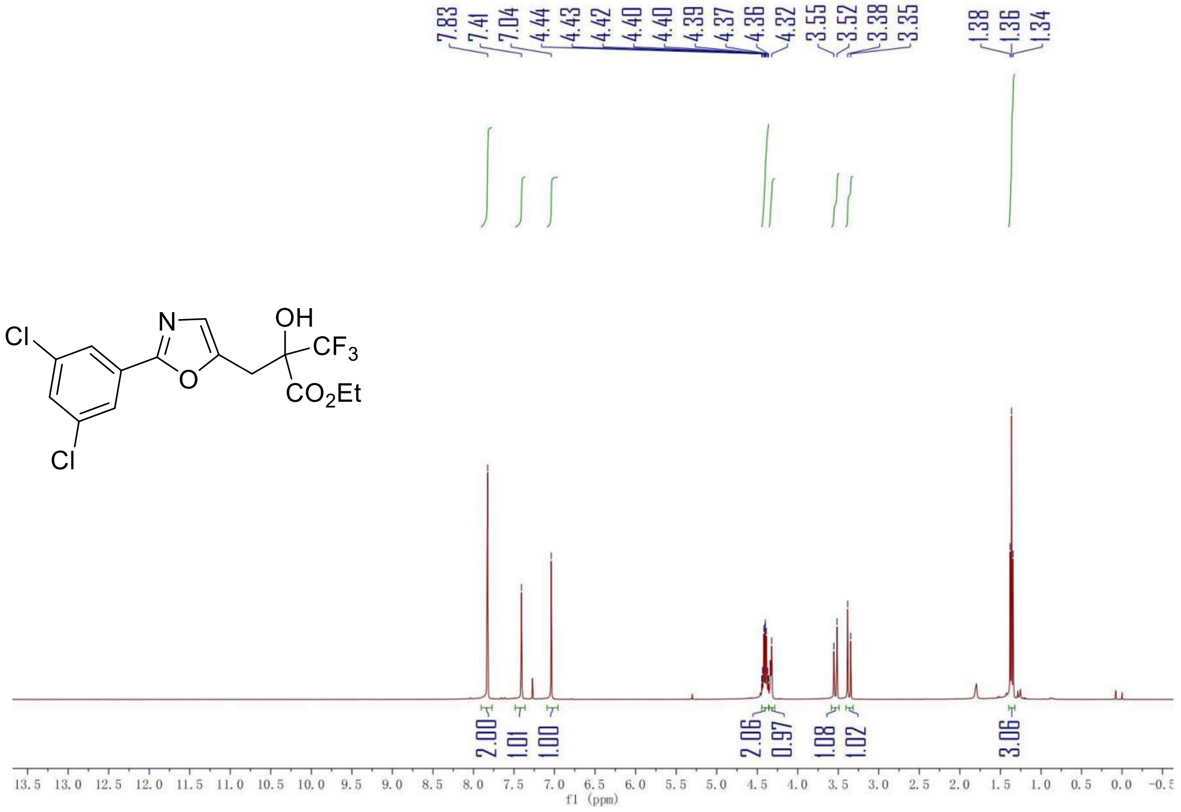

^{1}H NMR spectrum of compound **3p**

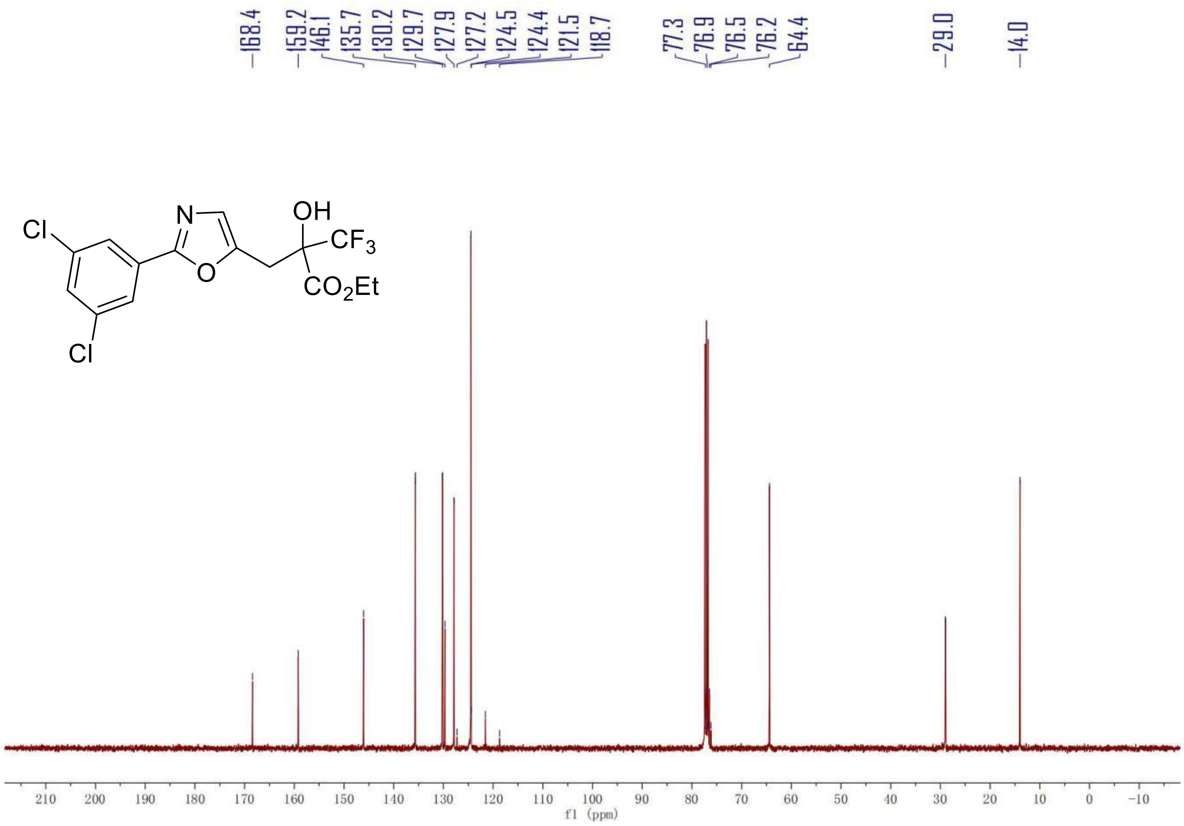

^{13}C NMR spectrum of compound **3p**

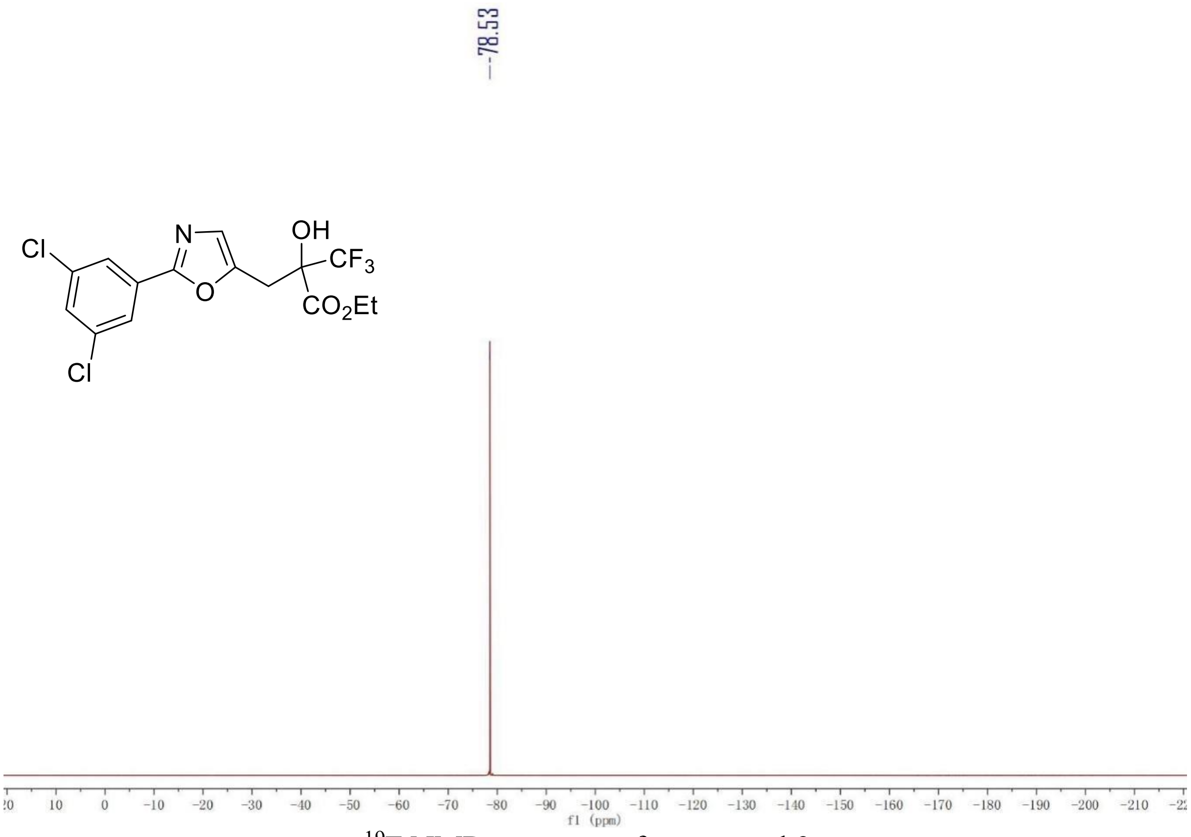

^{19}F NMR spectrum of compound **3p**

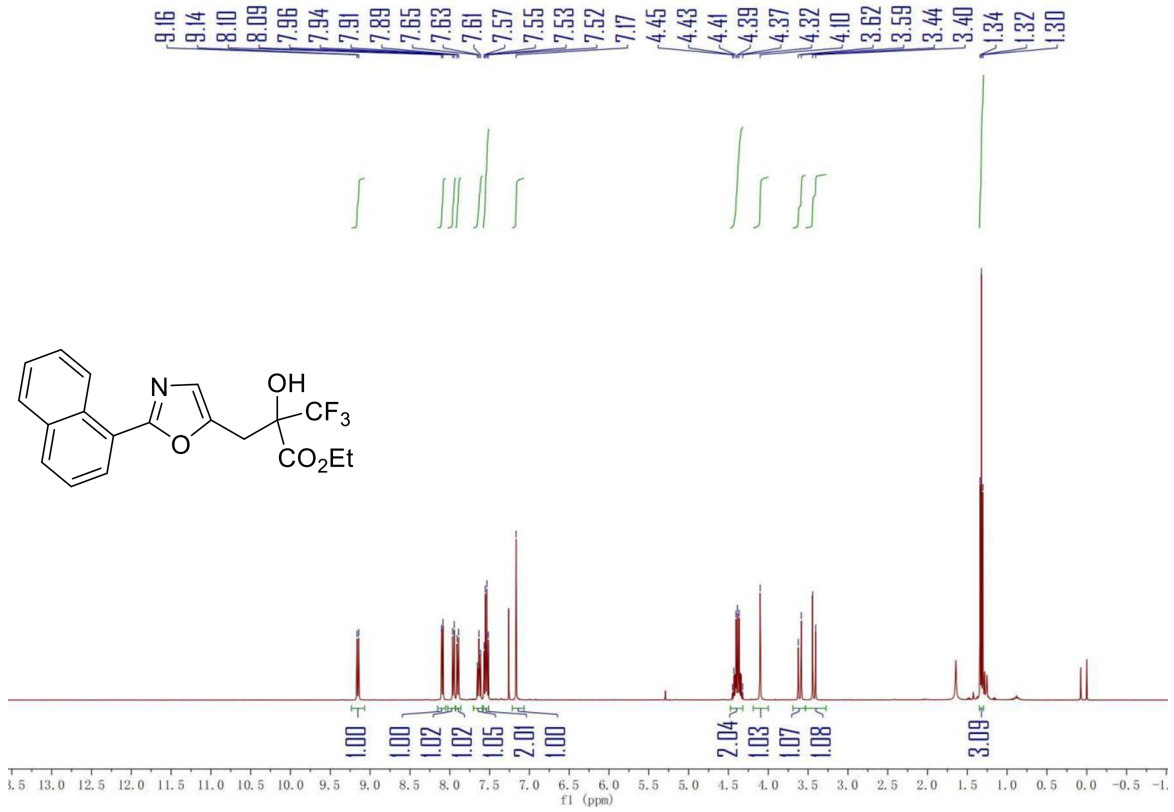

^{1}H NMR spectrum of compound **3q**

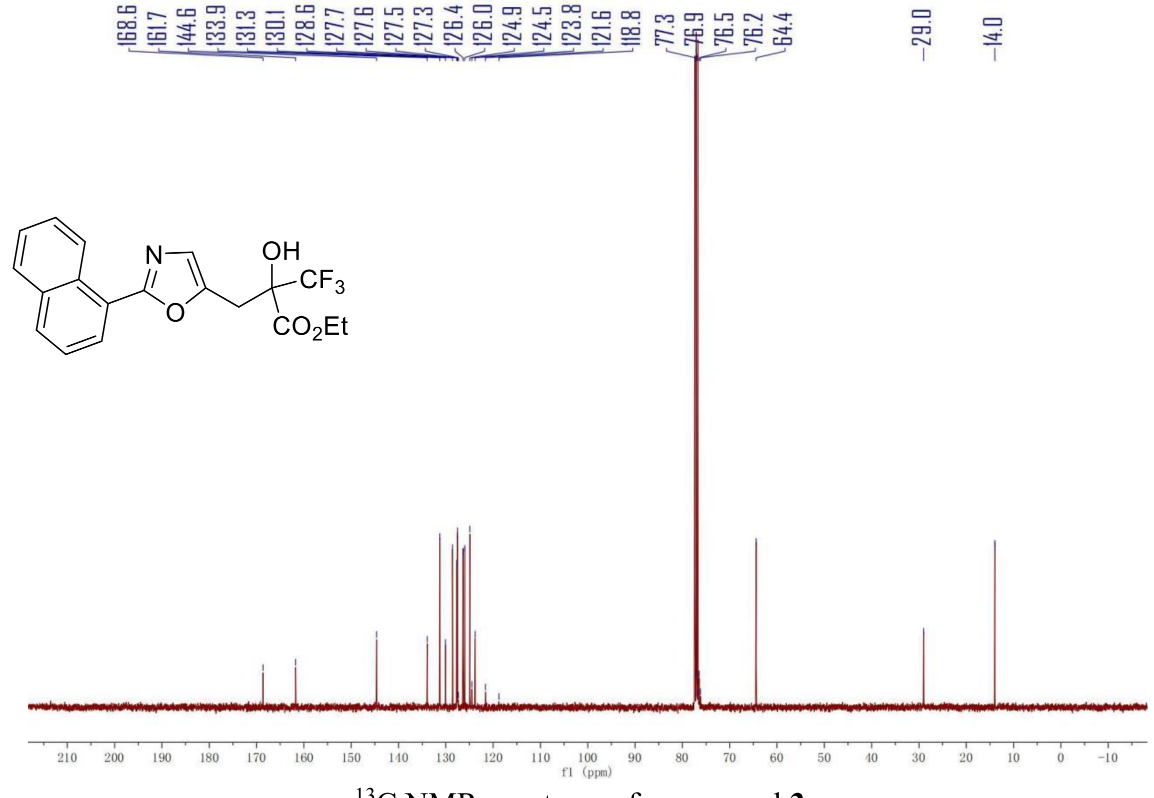

^{13}C NMR spectrum of compound **3q**

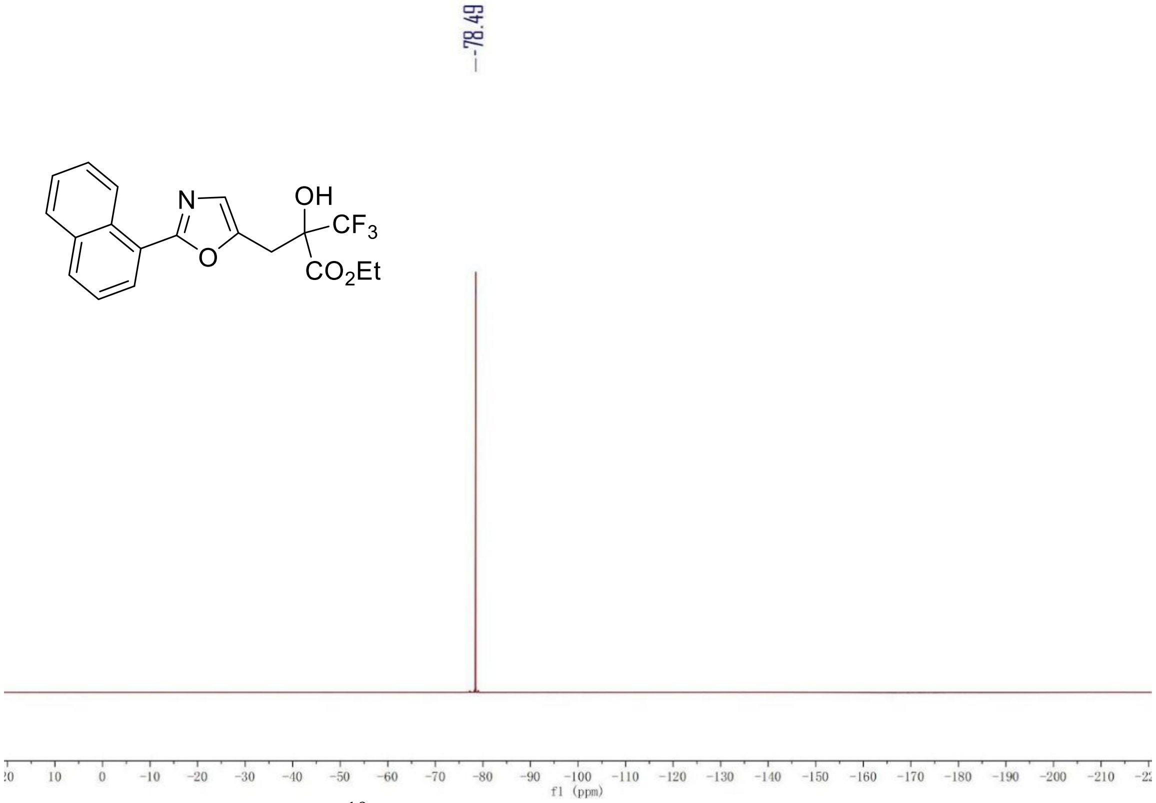

^{19}F NMR spectrum of compound **3q**

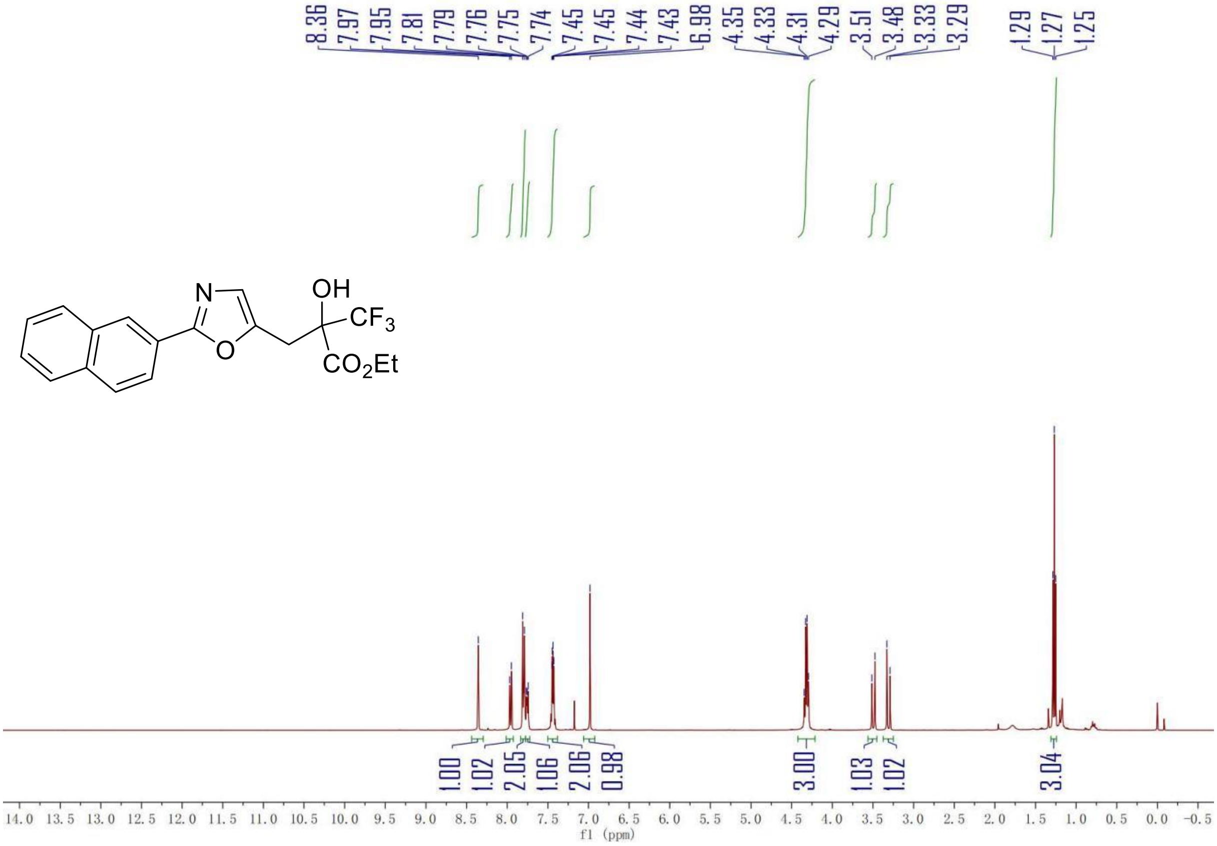

^{1}H NMR spectrum of compound **3r**

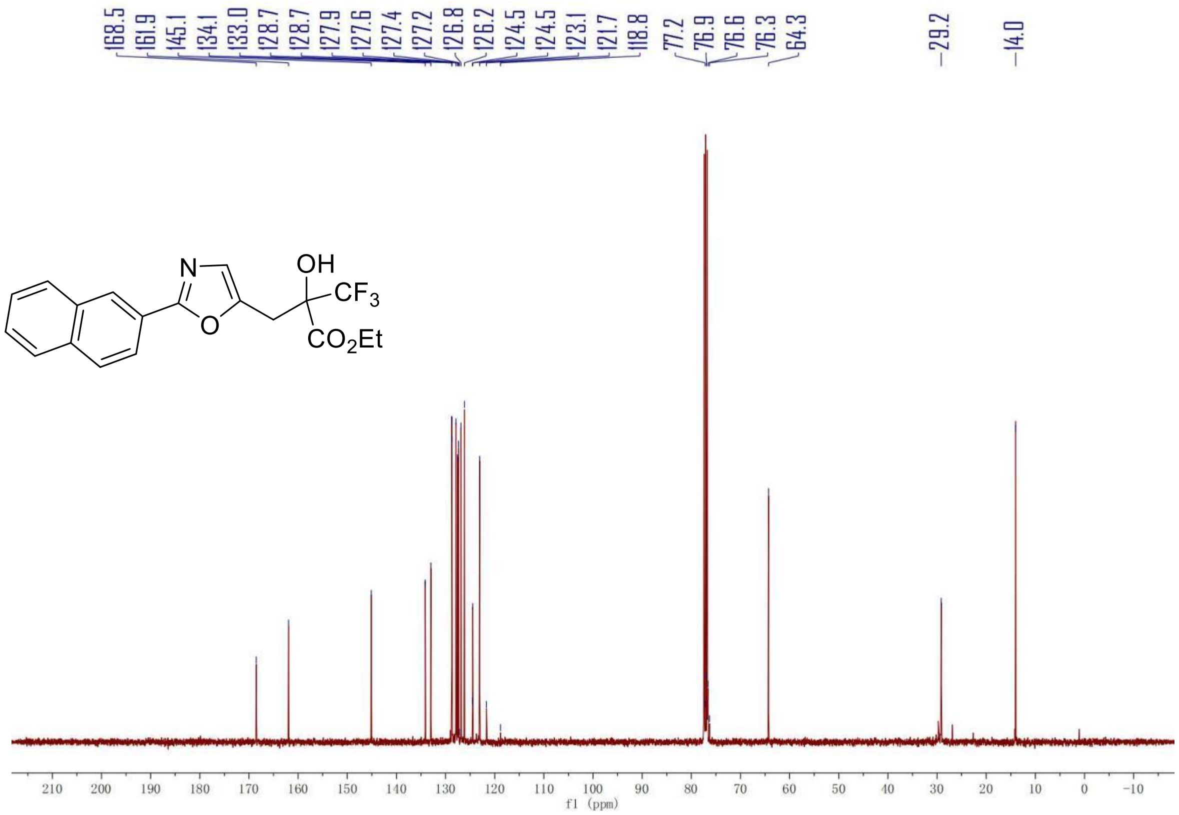

^{13}C NMR spectrum of compound **3r**

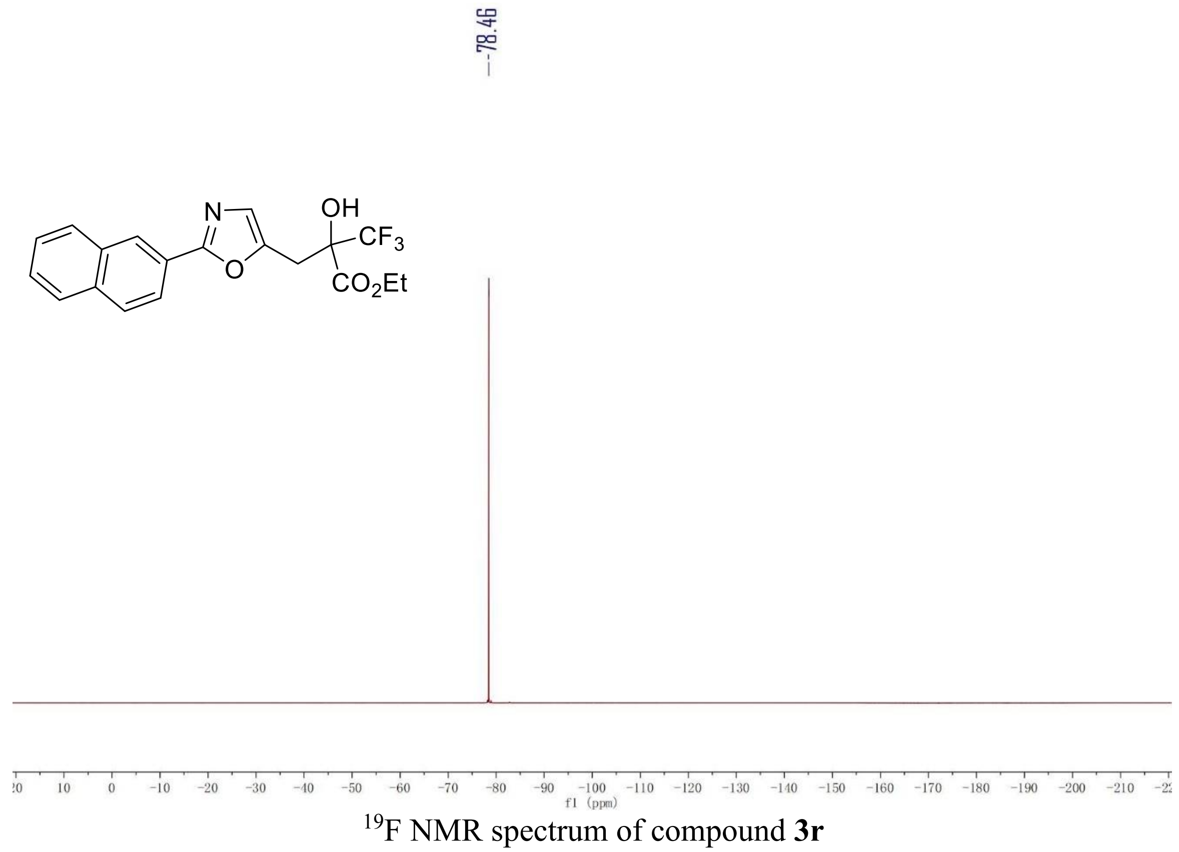

^{19}F NMR spectrum of compound **3r**

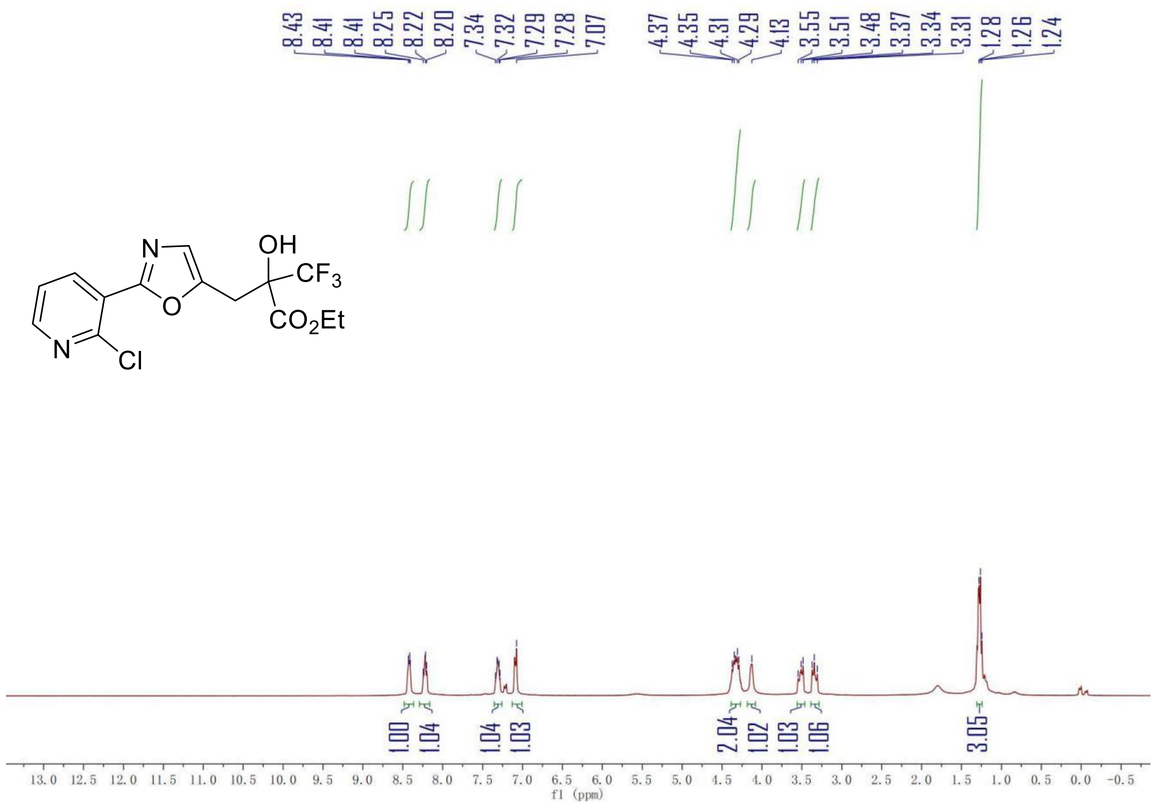

^{1}H NMR spectrum of compound **3s**

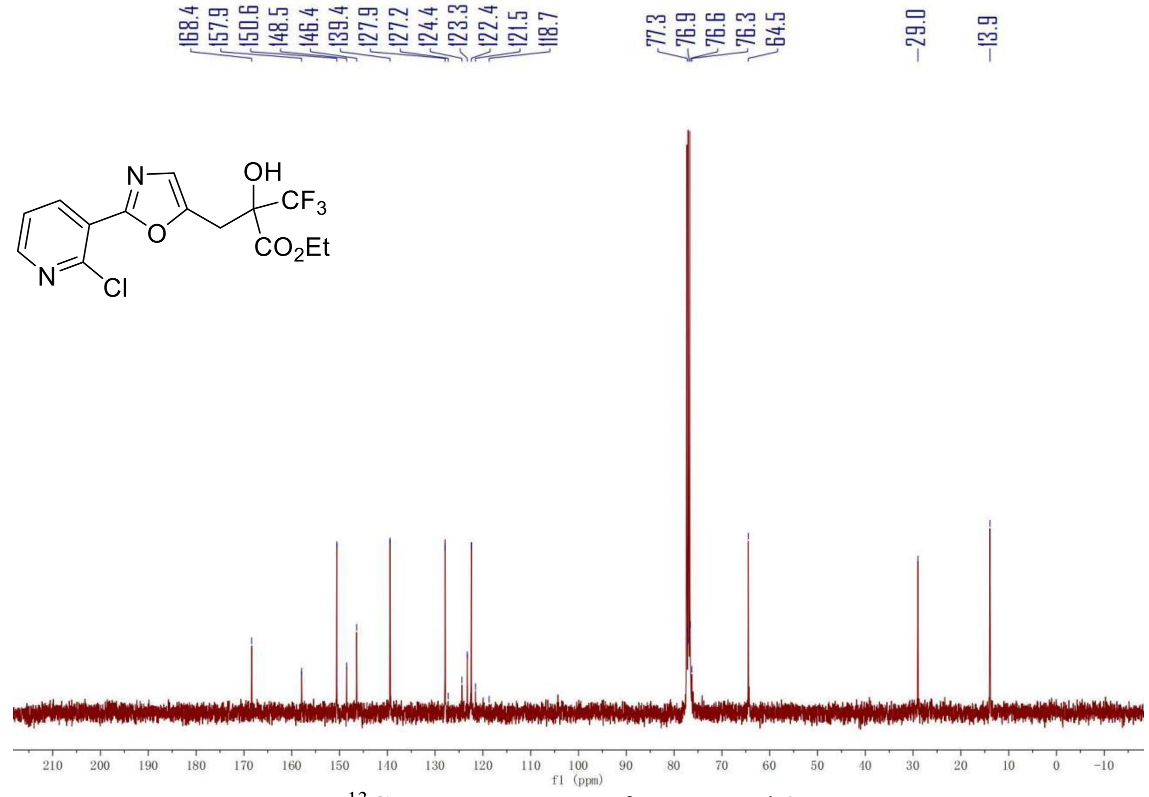

^{13}C NMR spectrum of compound **3s**

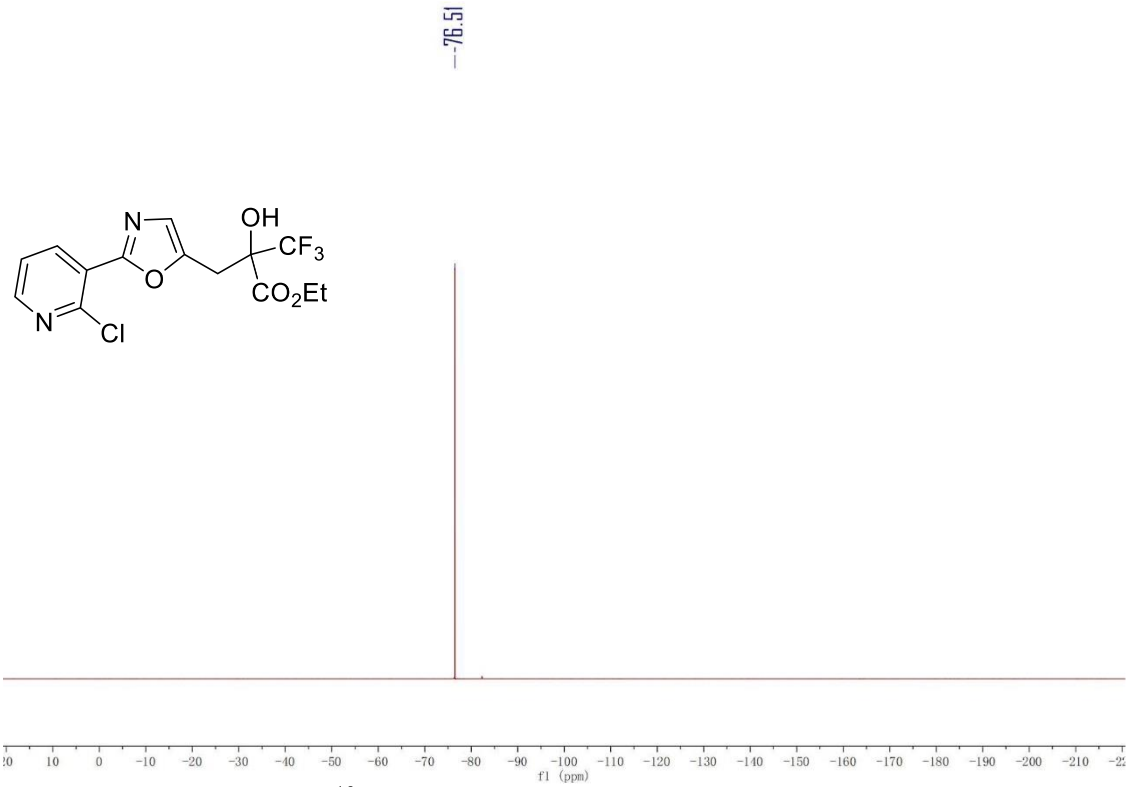

^{19}F NMR spectrum of compound **3s**

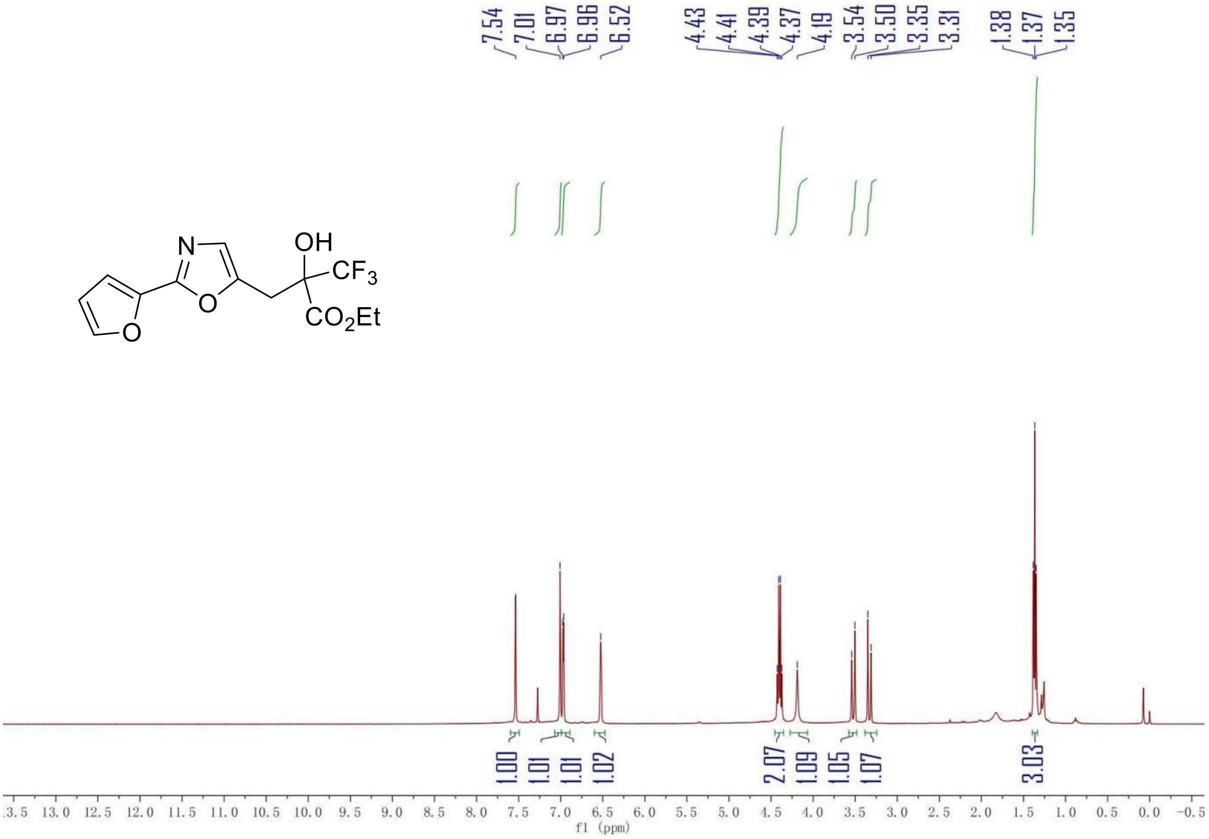

^{1}H NMR spectrum of compound **3t**

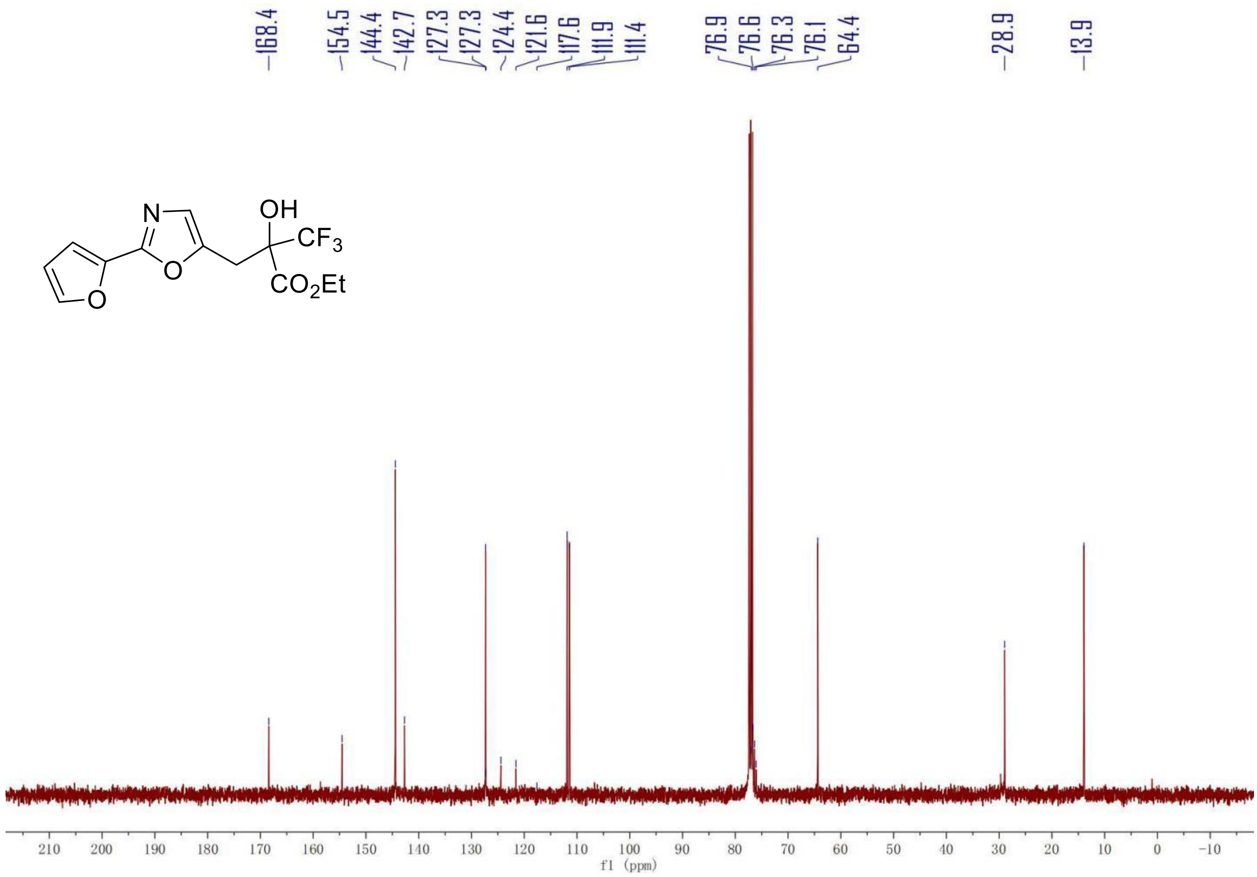

^{13}C NMR spectrum of compound **3t**

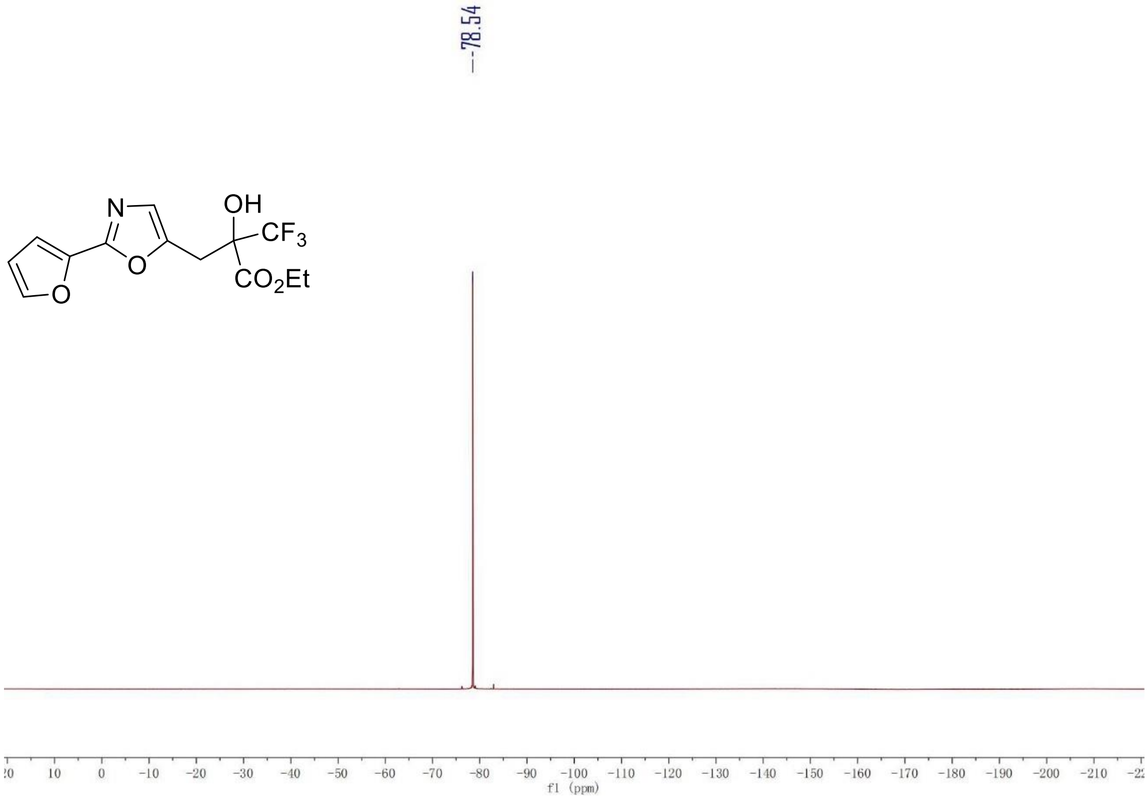

^{19}F NMR spectrum of compound **3t**

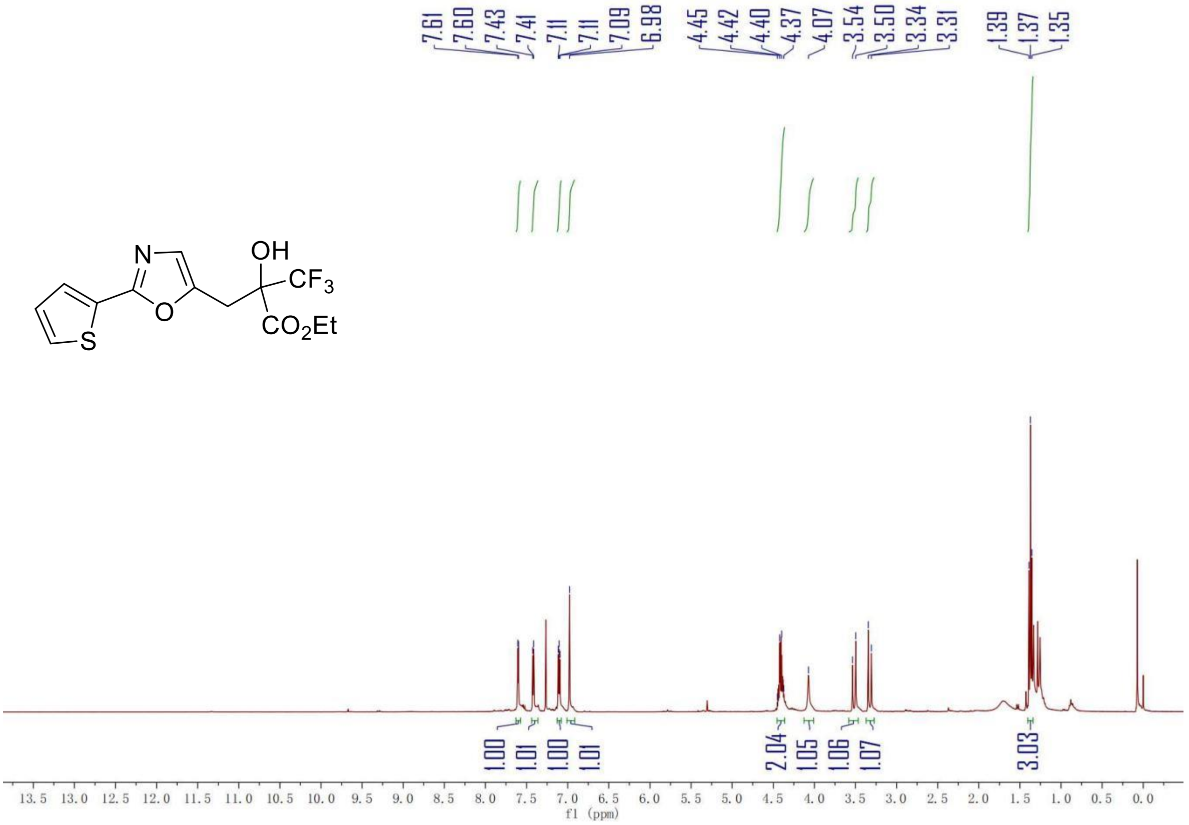

^{1}H NMR spectrum of compound **3u**

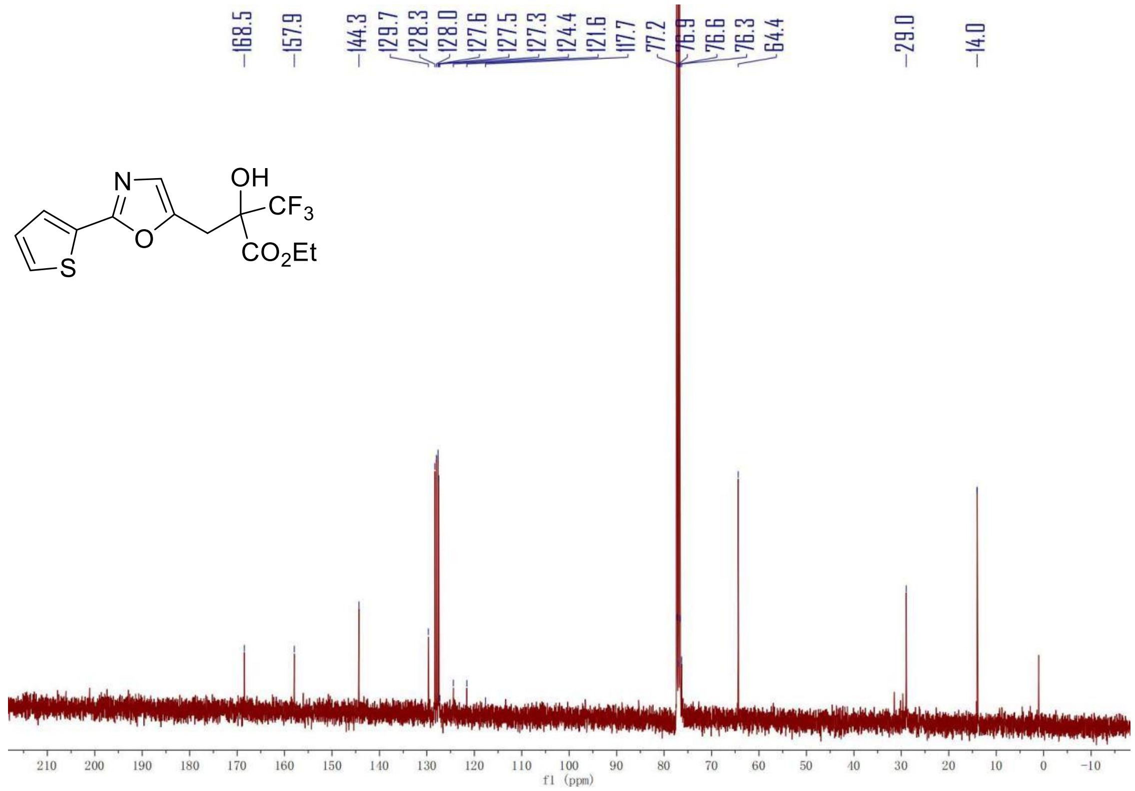

^{13}C NMR spectrum of compound **3u**

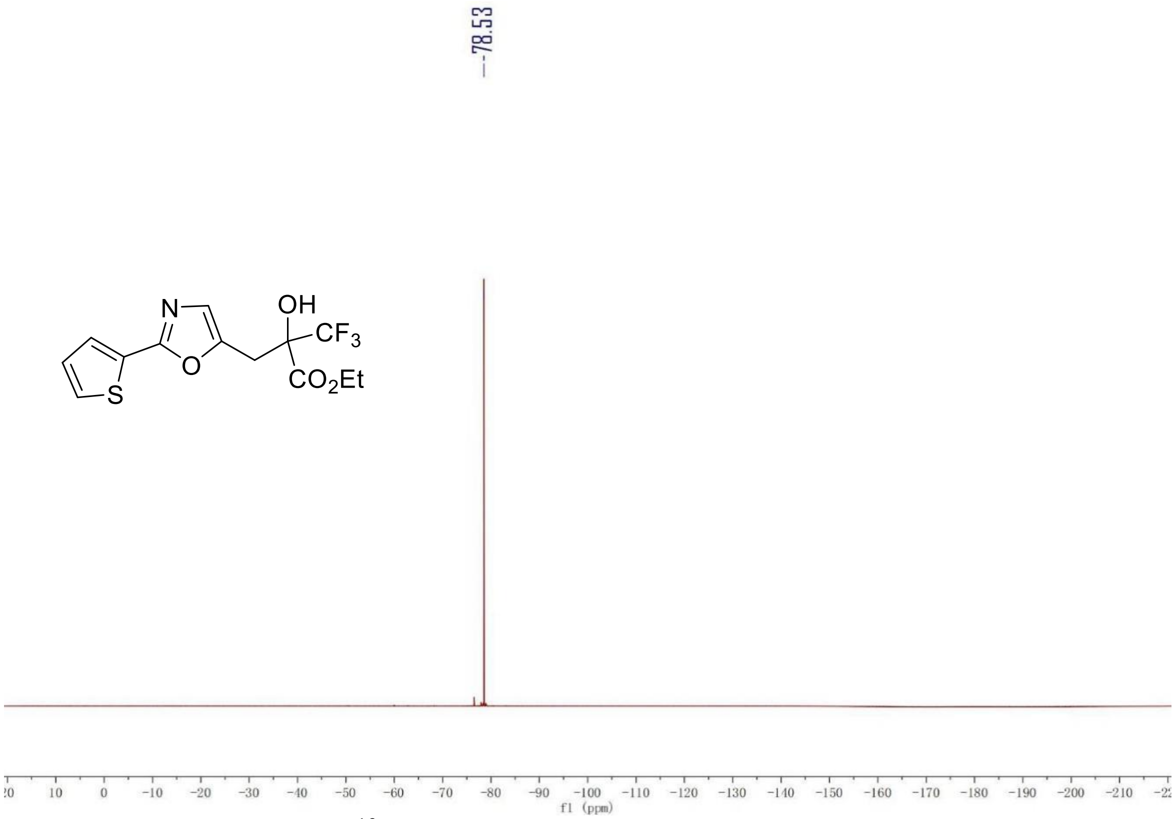

^{19}F NMR spectrum of compound **3u**

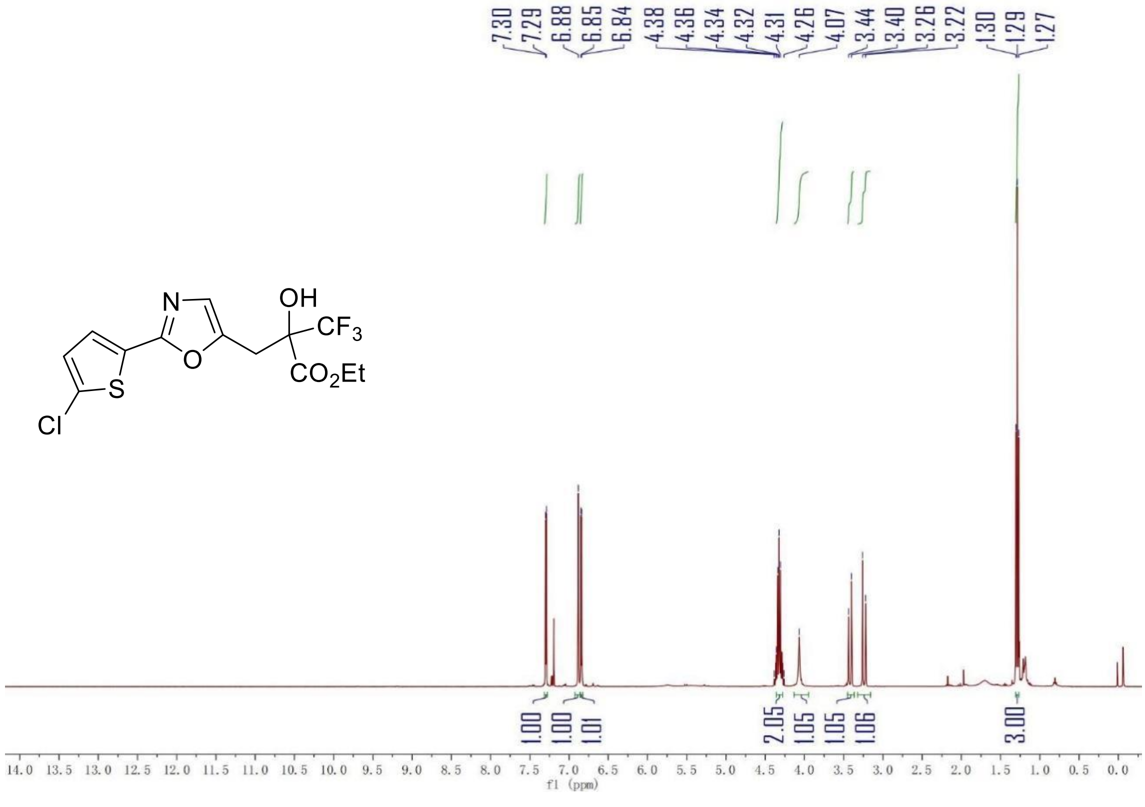

^{1}H NMR spectrum of compound **3v**

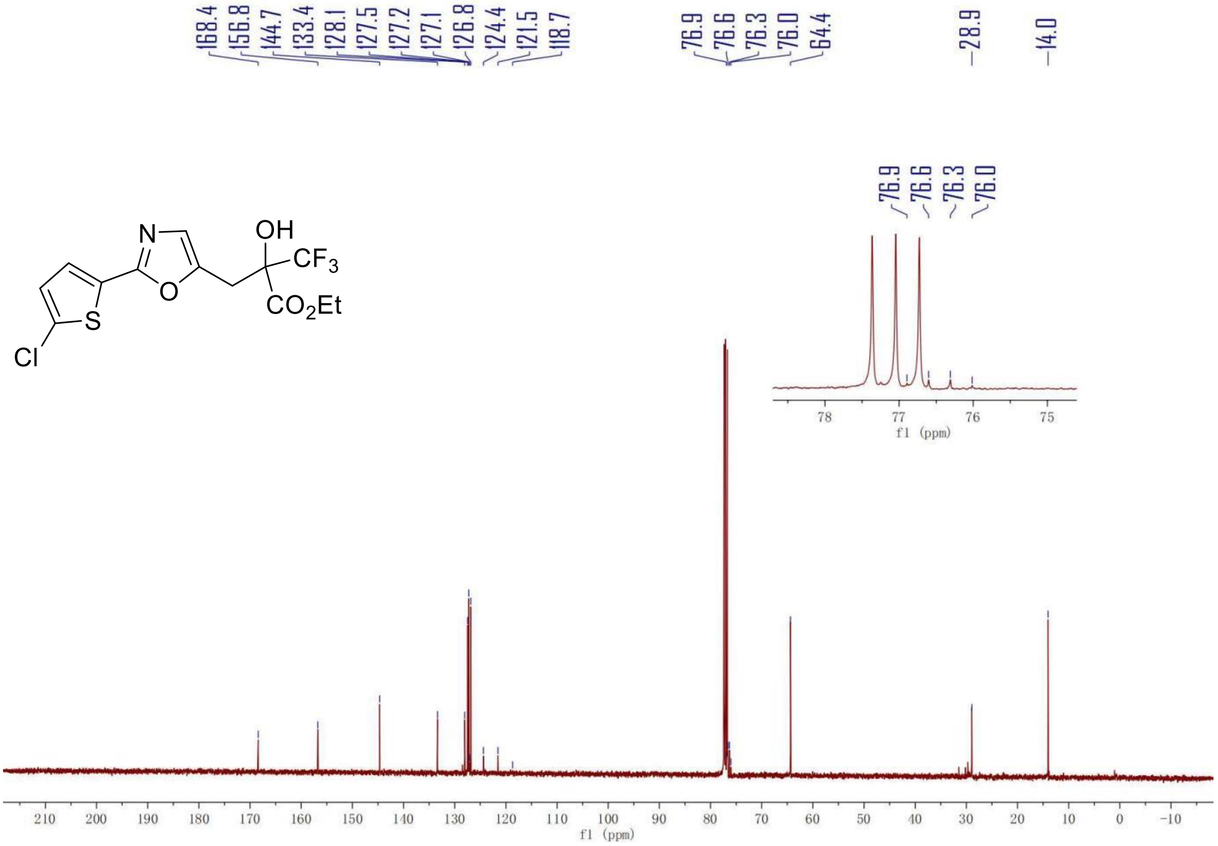

^{13}C NMR spectrum of compound **3v**

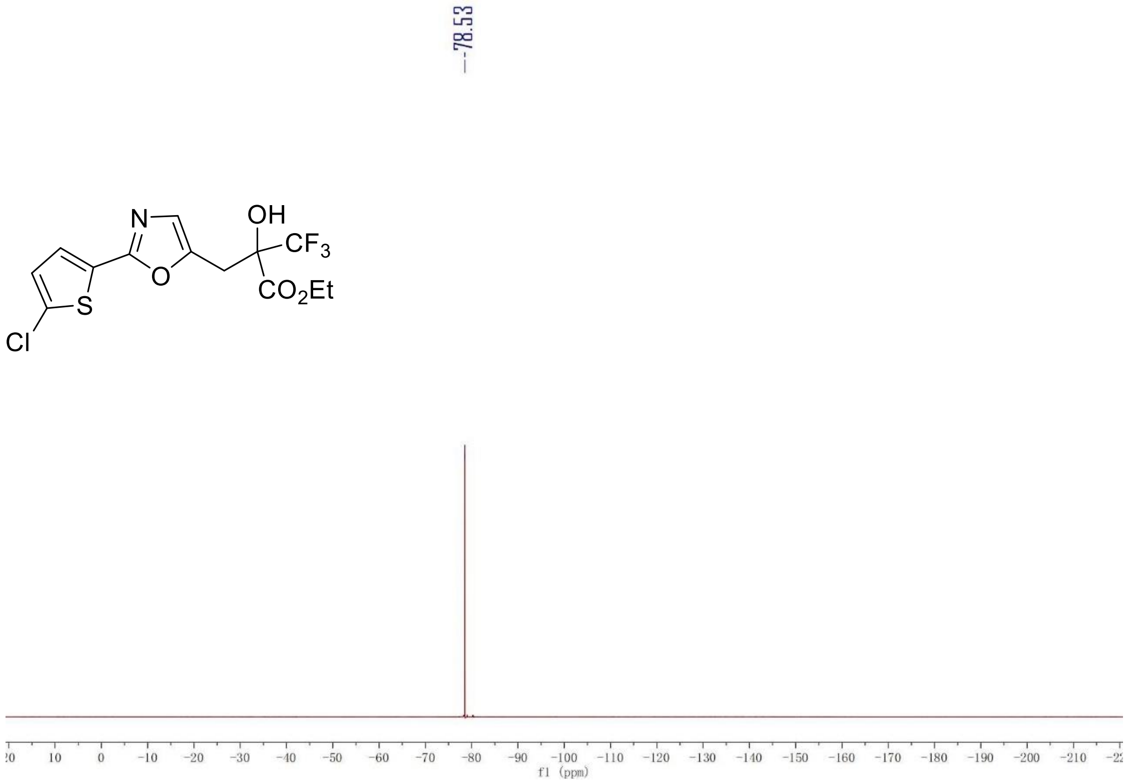

^{19}F NMR spectrum of compound **3v**

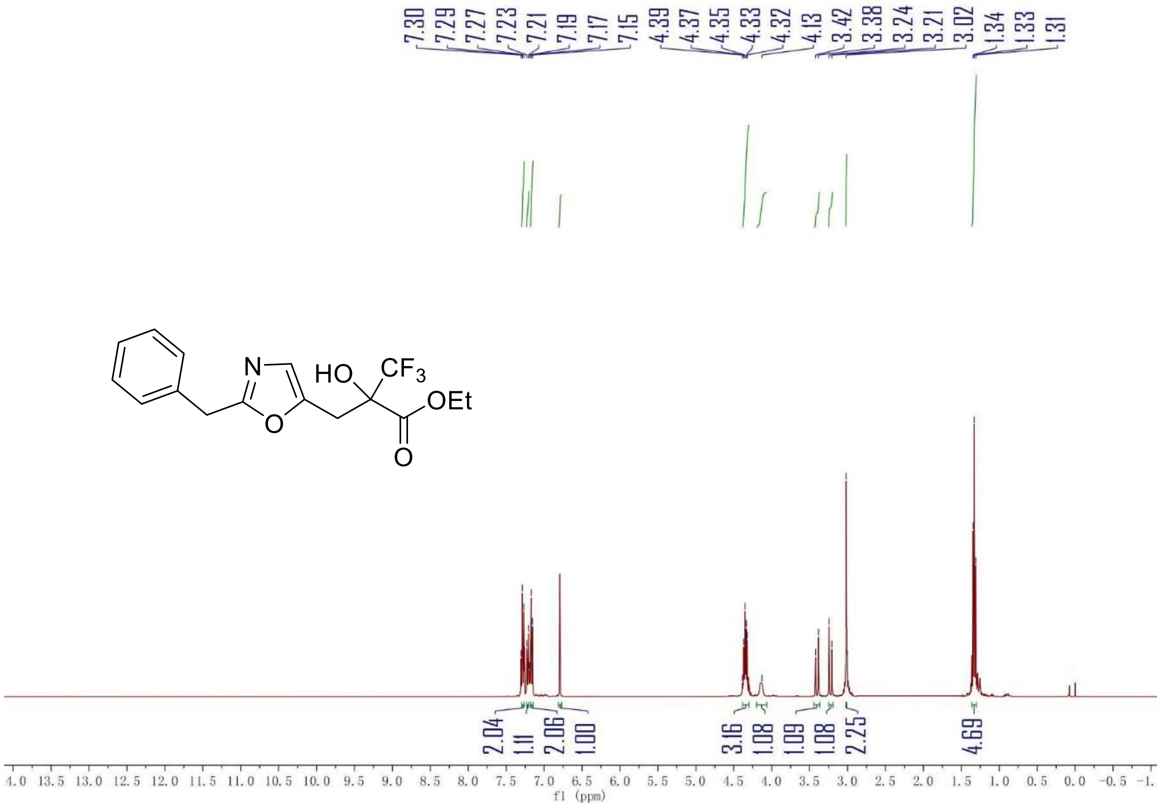

^{1}H NMR spectrum of compound **3w**

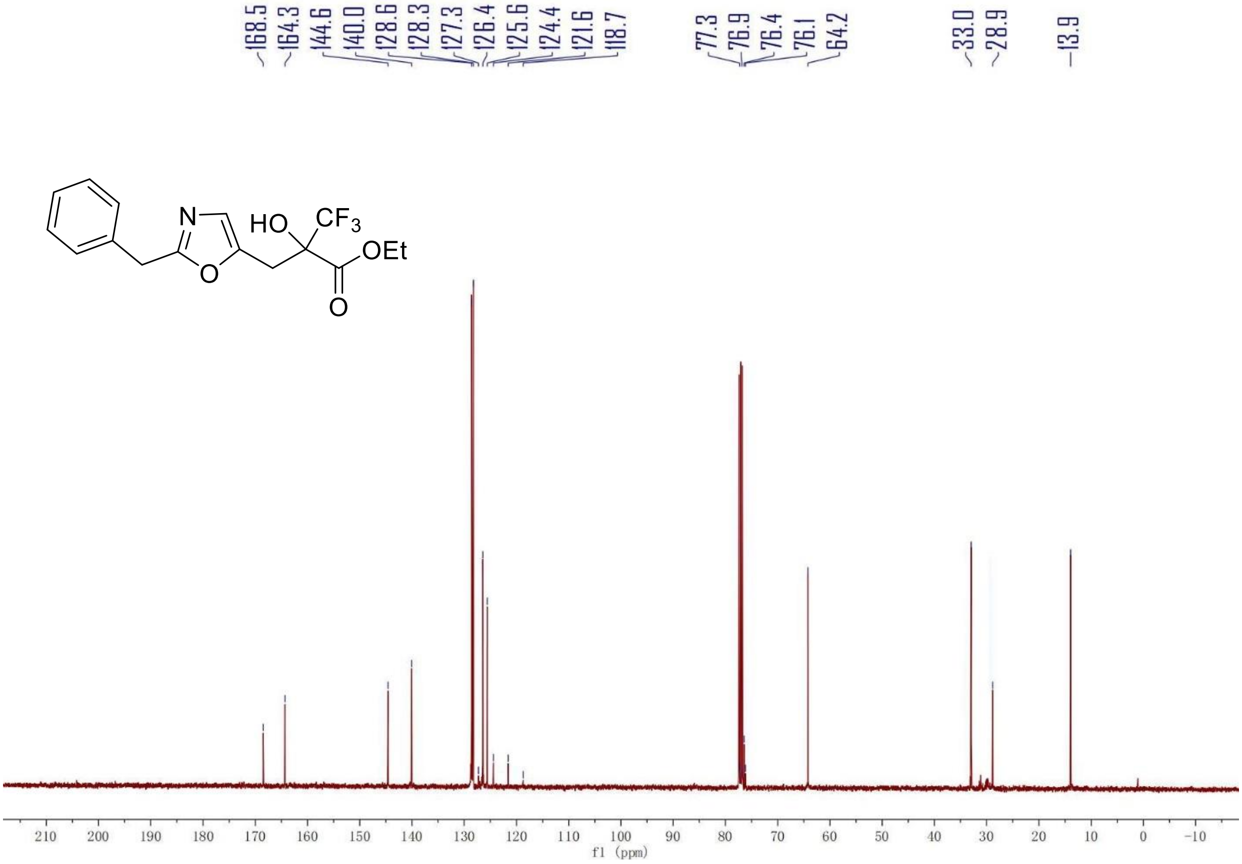

^{13}C NMR spectrum of compound **3w**

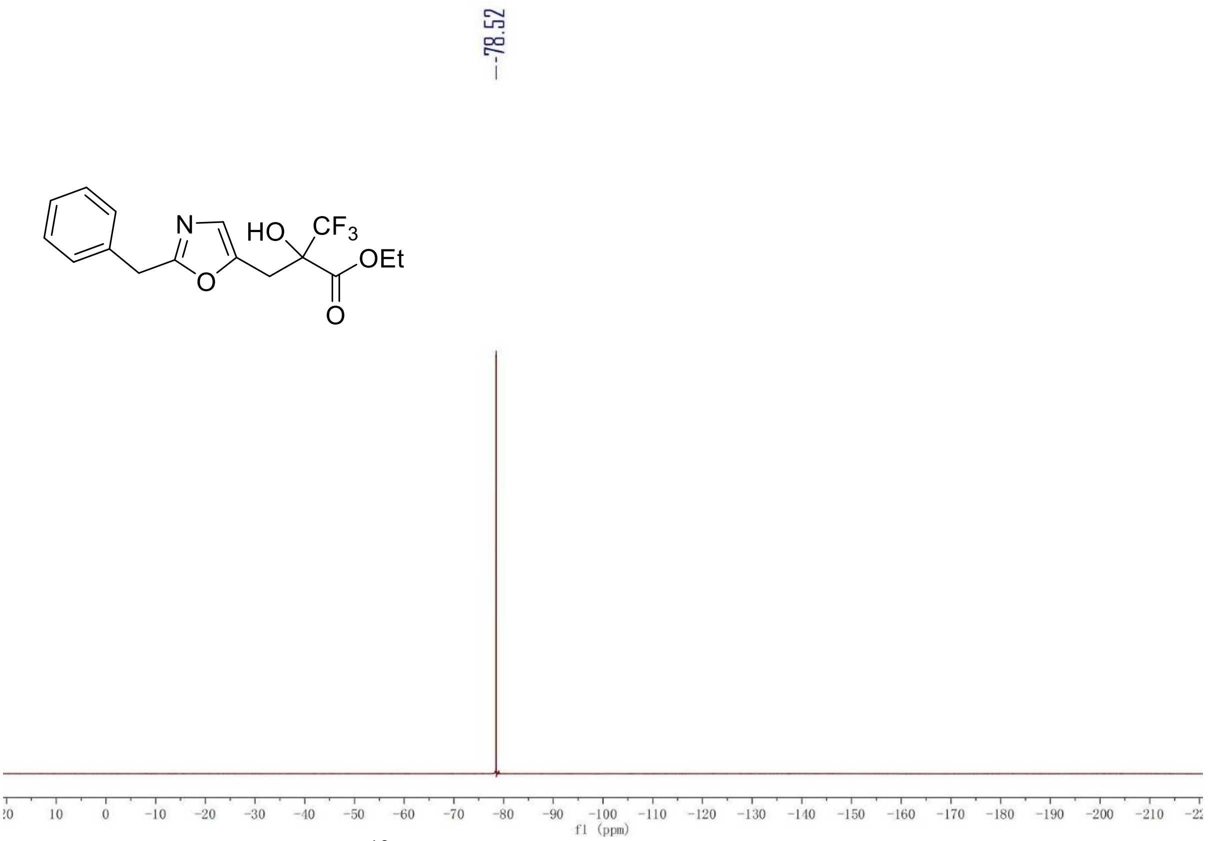

^{19}F NMR spectrum of compound **3w**

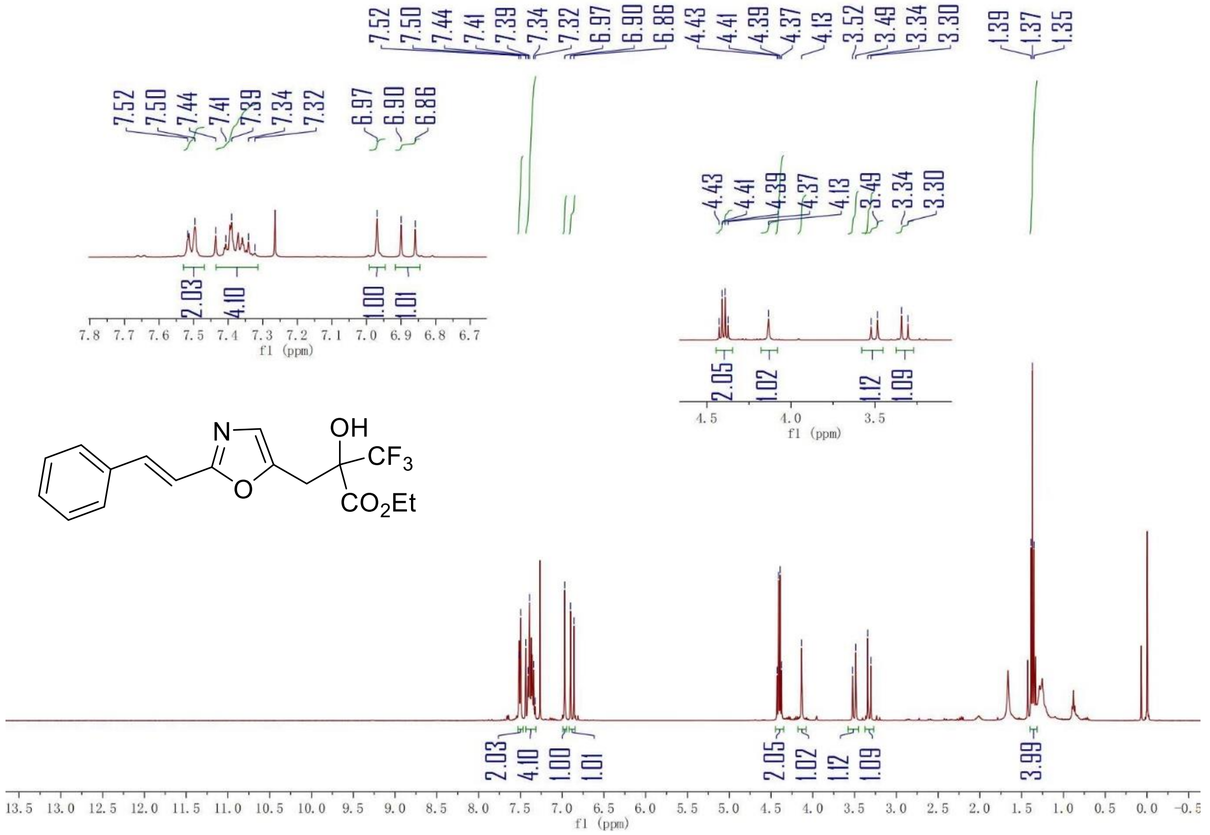

^{1}H NMR spectrum of compound **3x**

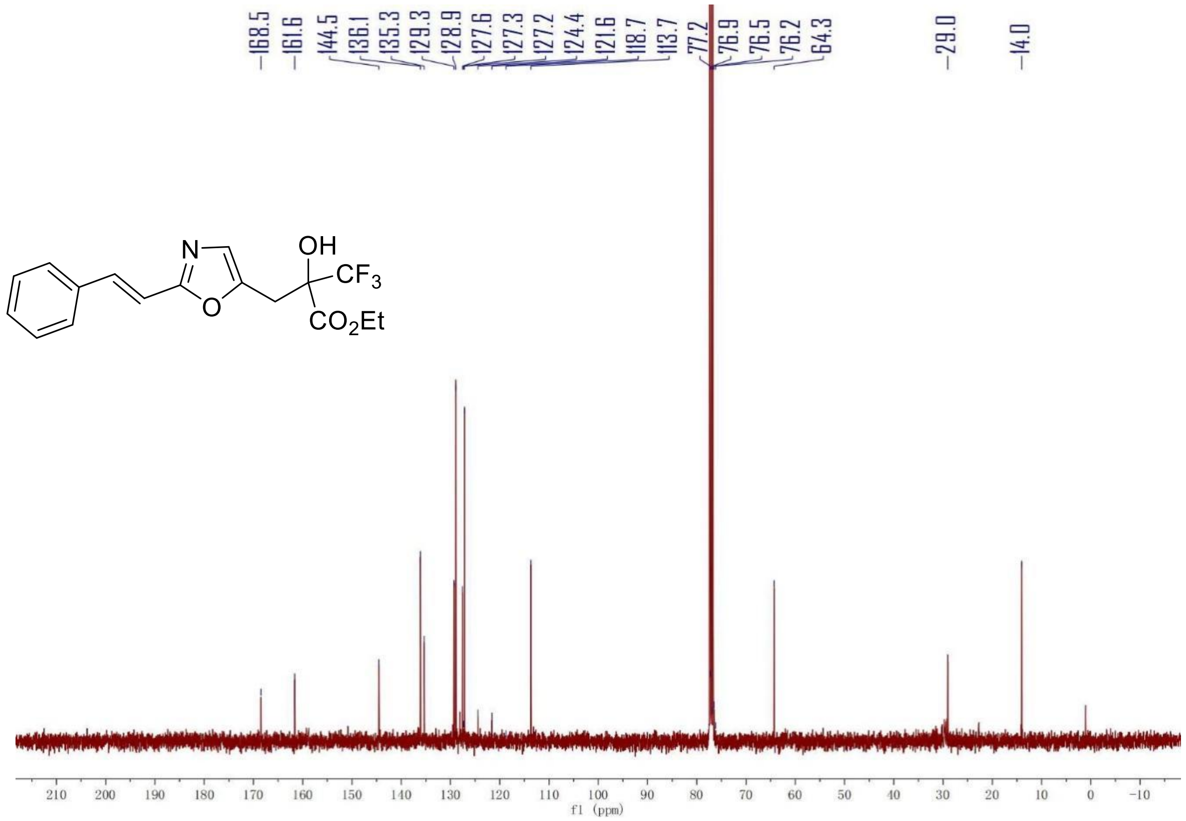

^{13}C NMR spectrum of compound **3x**

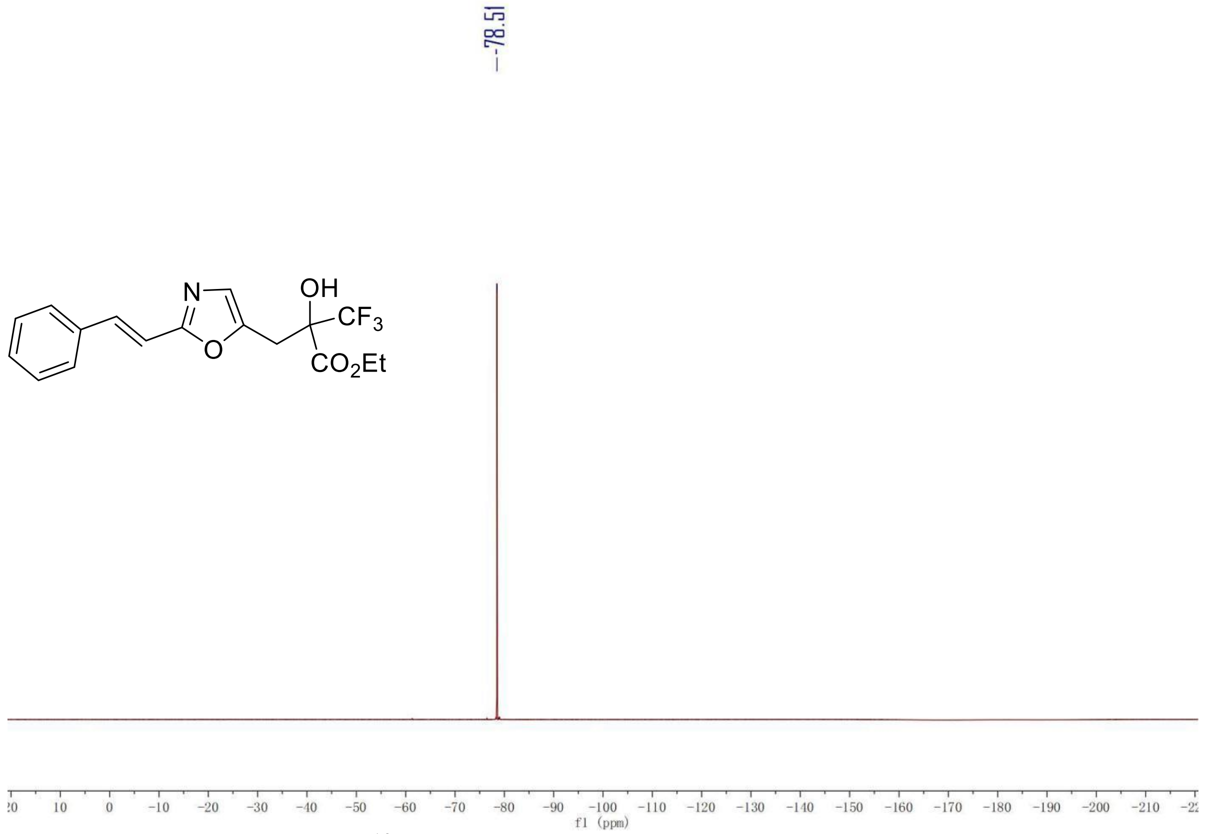

^{19}F NMR spectrum of compound **3x**

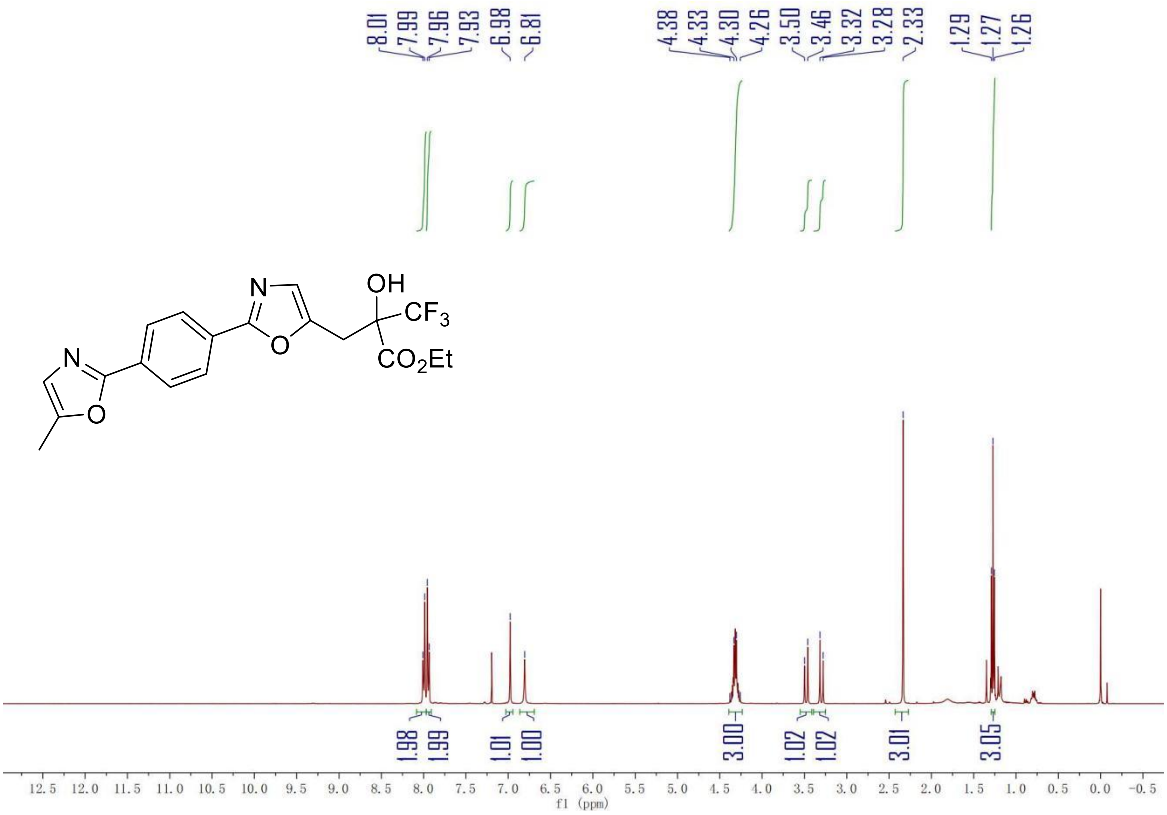

^{1}H NMR spectrum of compound **3y**

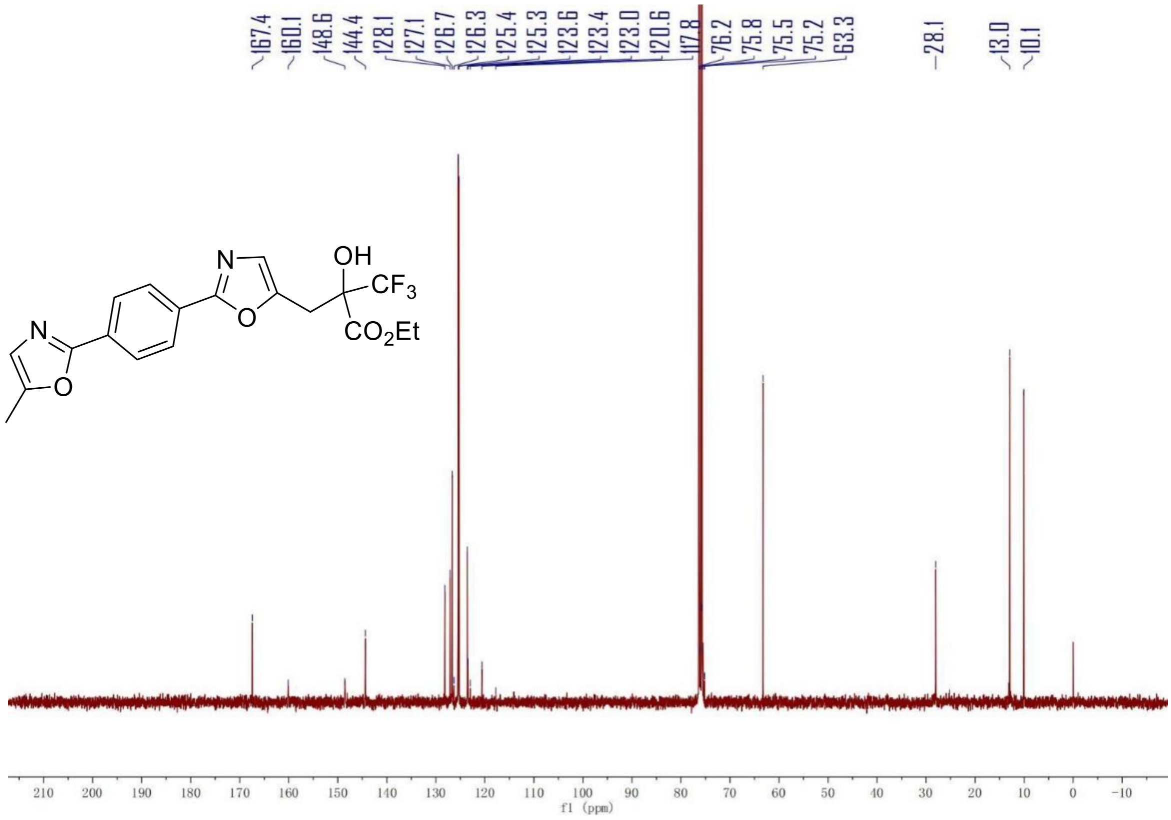

^{13}C NMR spectrum of compound **3y**

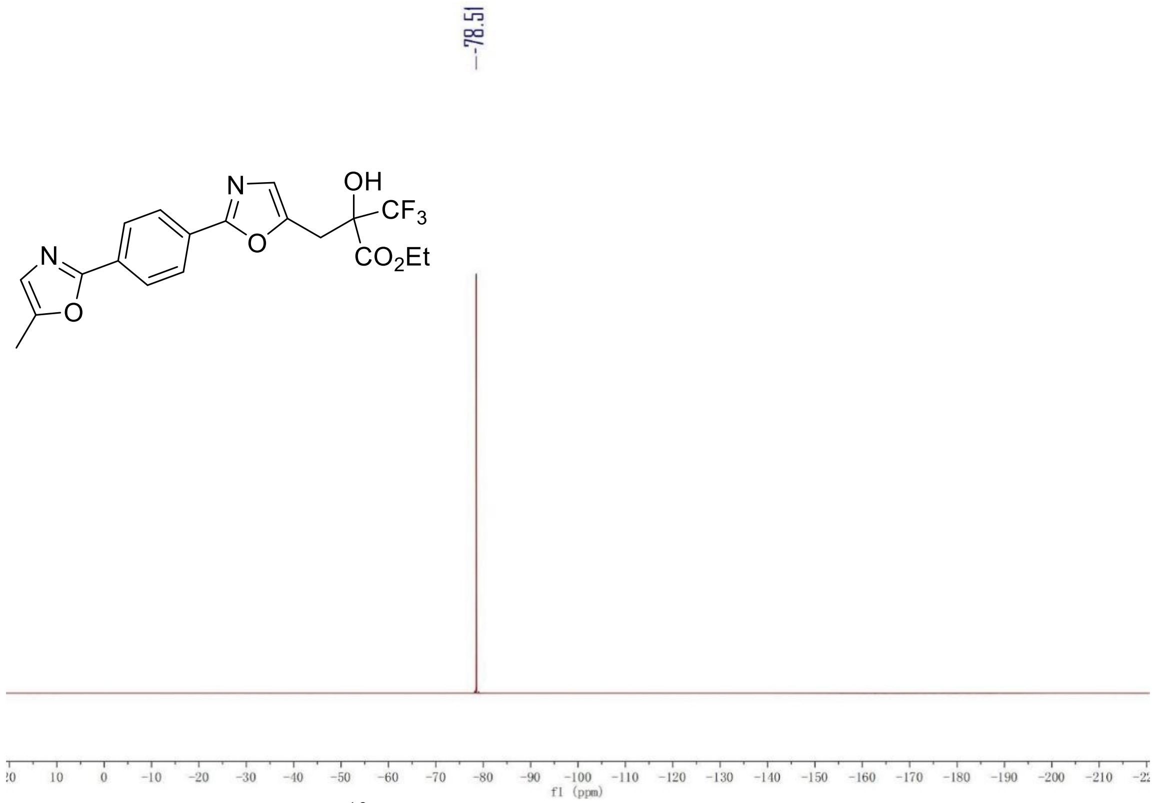

^{19}F NMR spectrum of compound **3y**

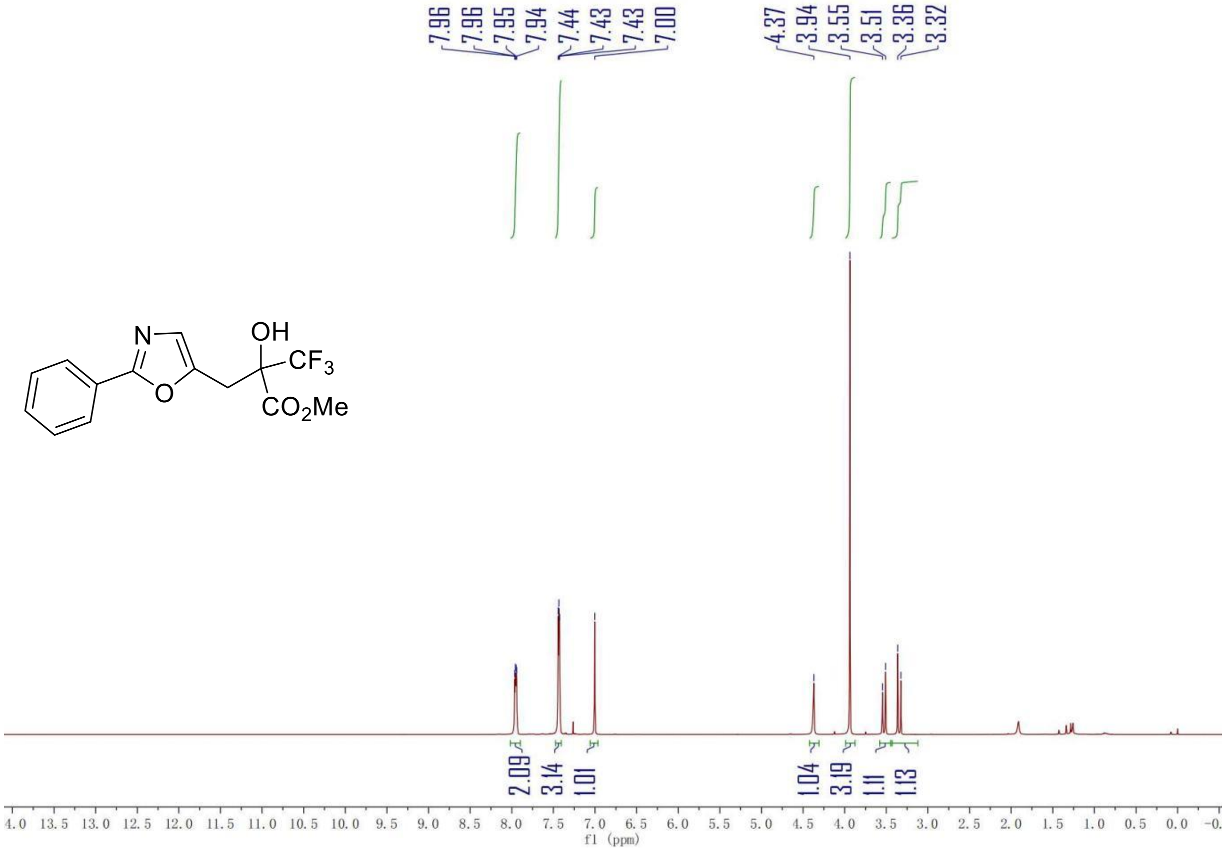

^{1}H NMR spectrum of compound **3z**

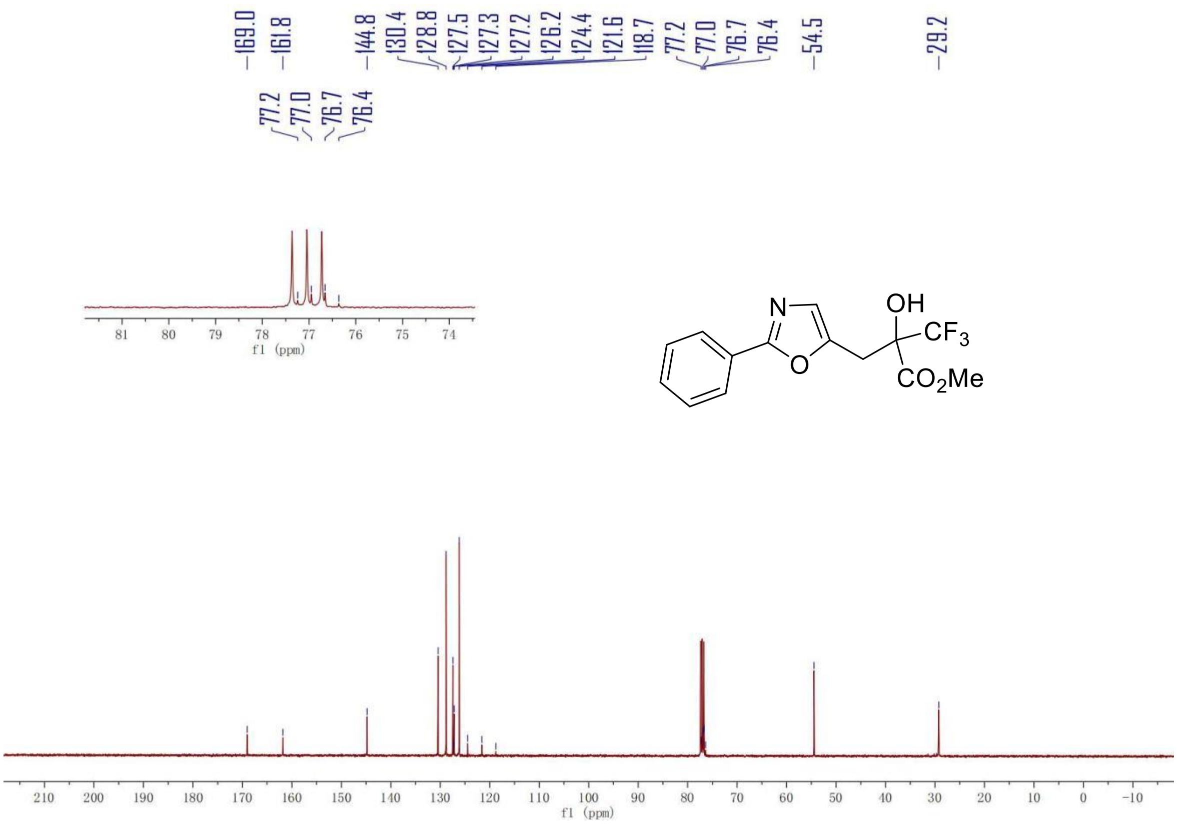

^{13}C NMR spectrum of compound **3z**

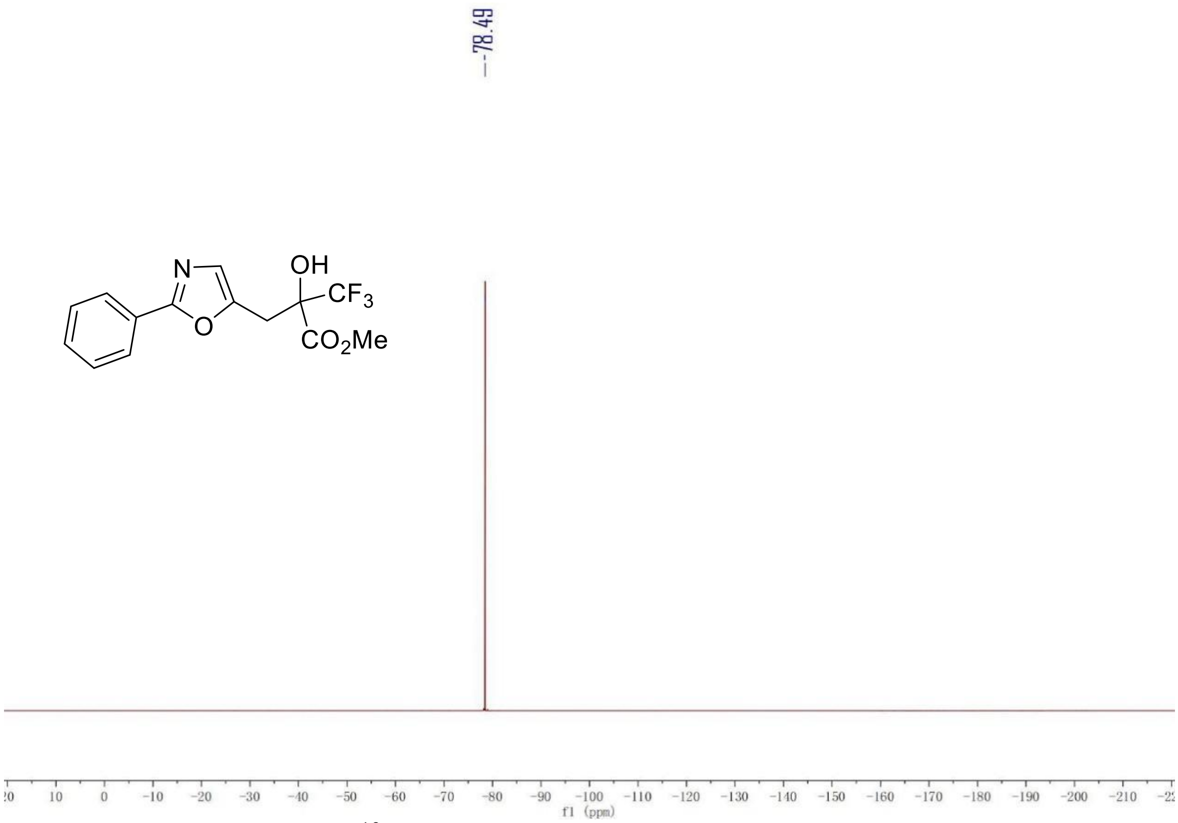

^{19}F NMR spectrum of compound **3z**

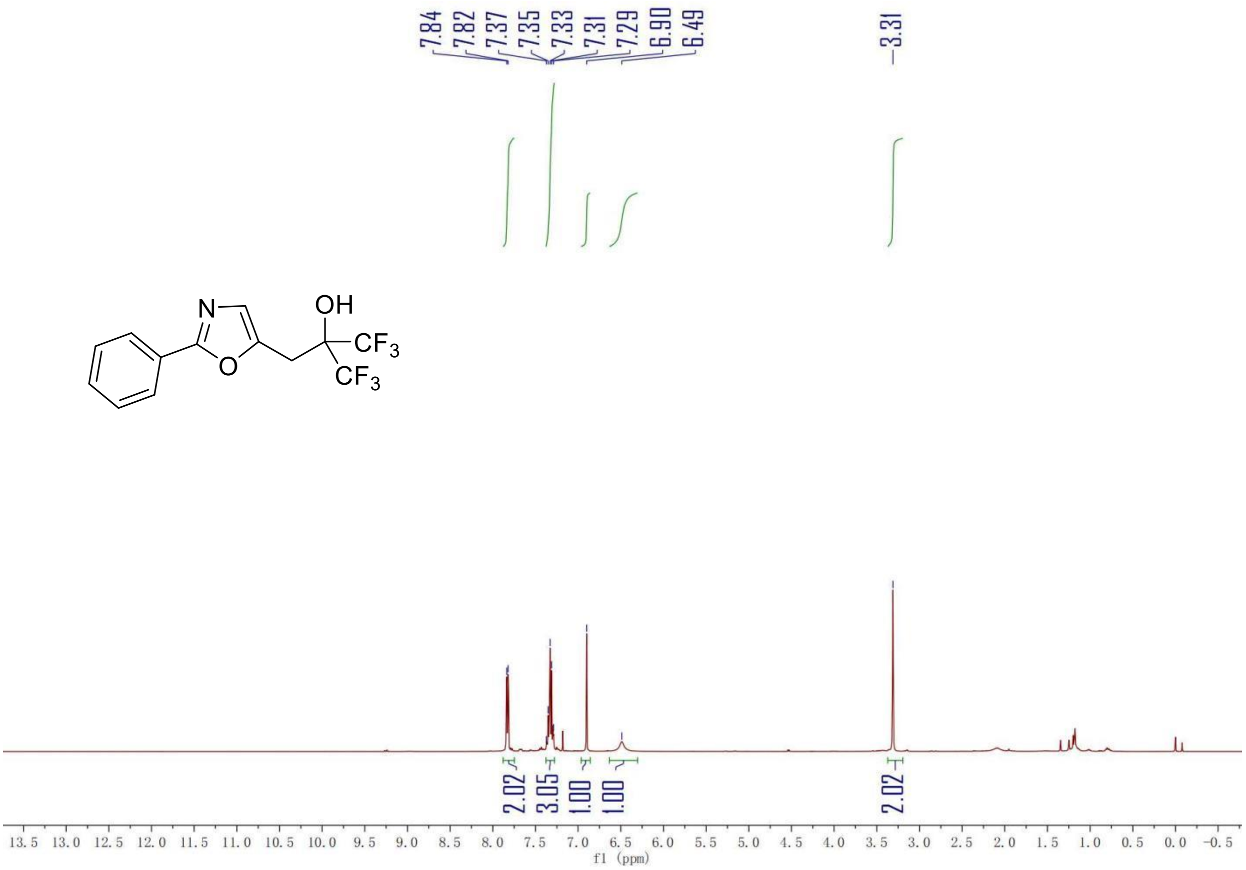

^{1}H NMR spectrum of compound **3aa**

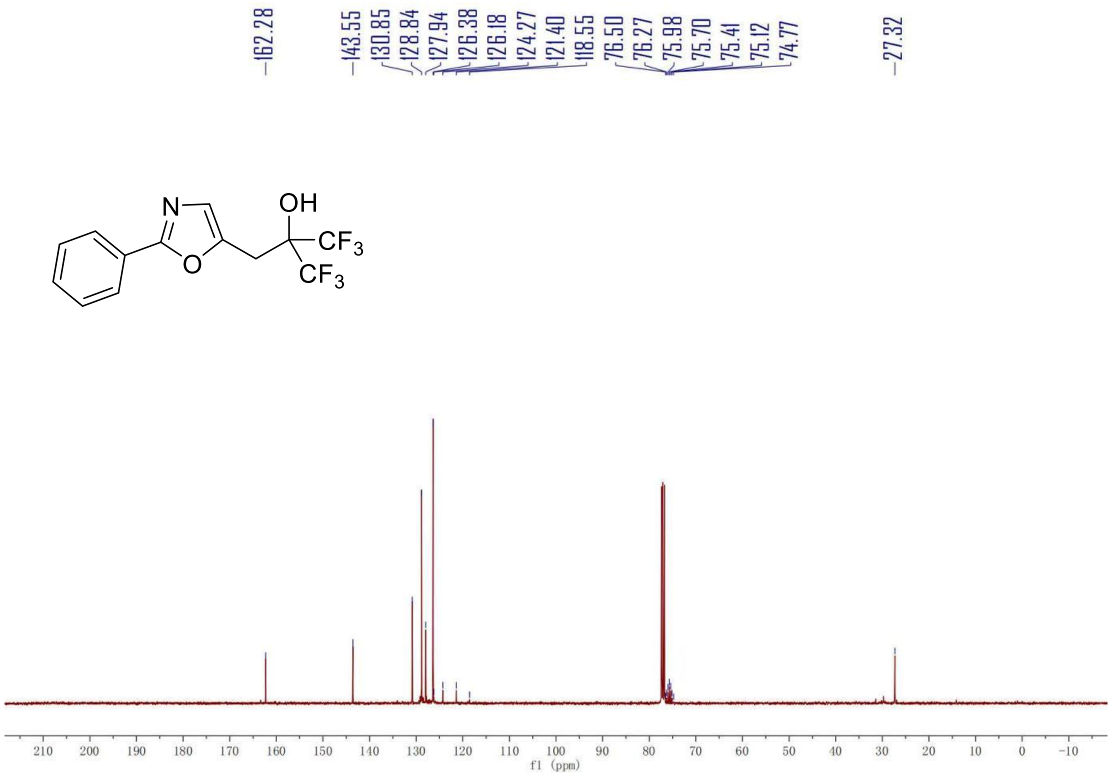

^{13}C NMR spectrum of compound **3aa**

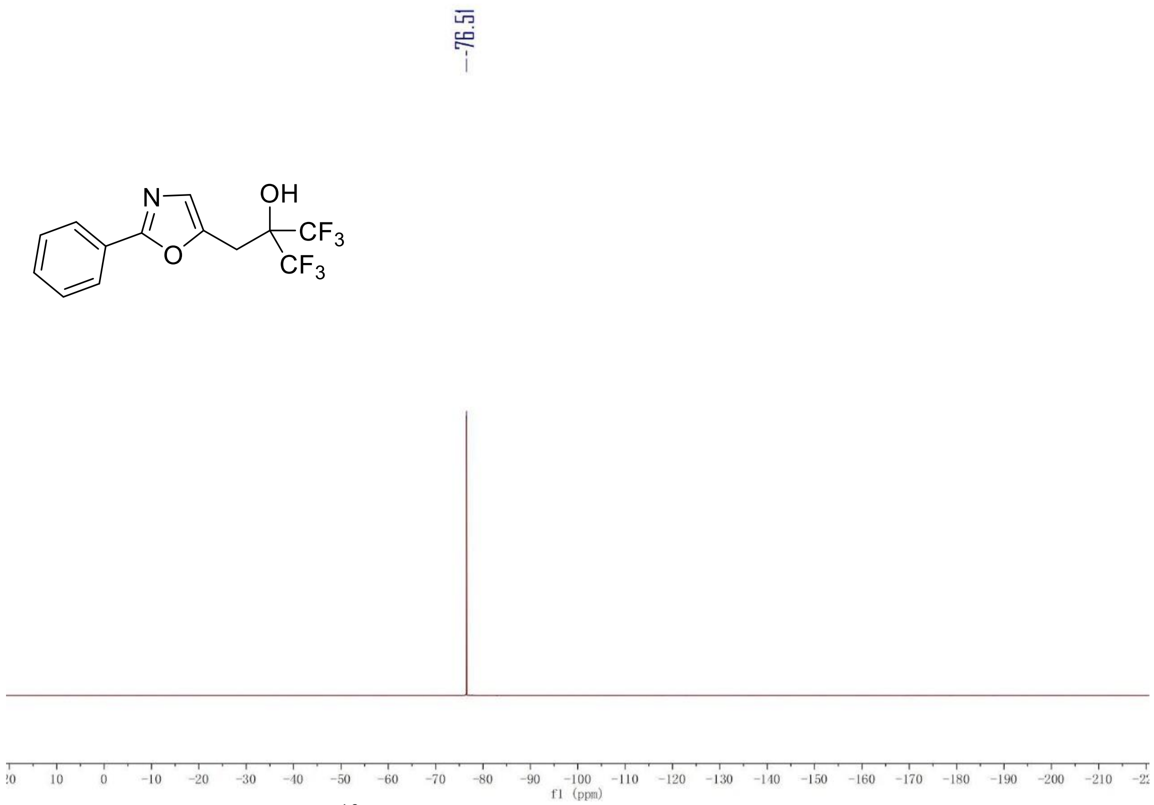

^{19}F NMR spectrum of compound **3aa**

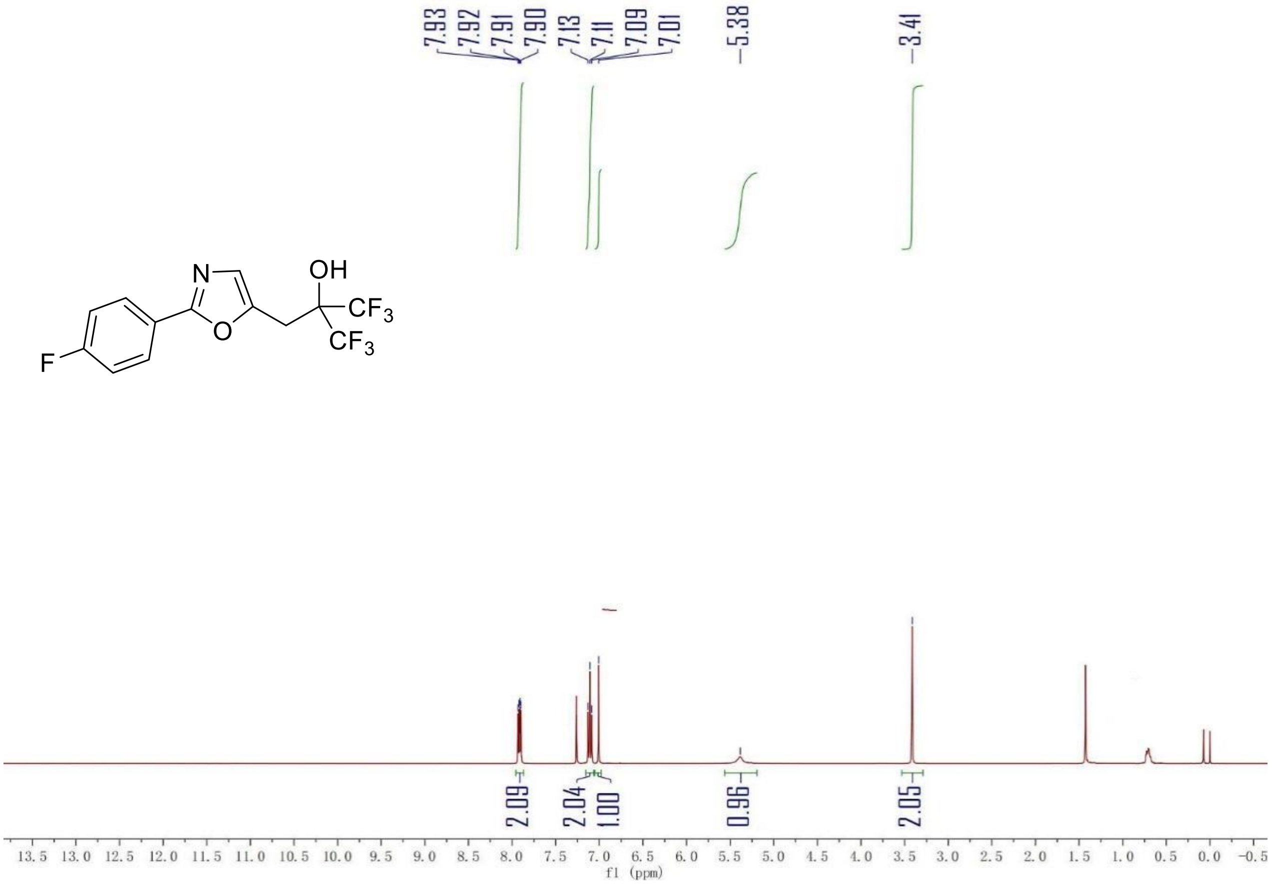

^{1}H NMR spectrum of compound **3ab**

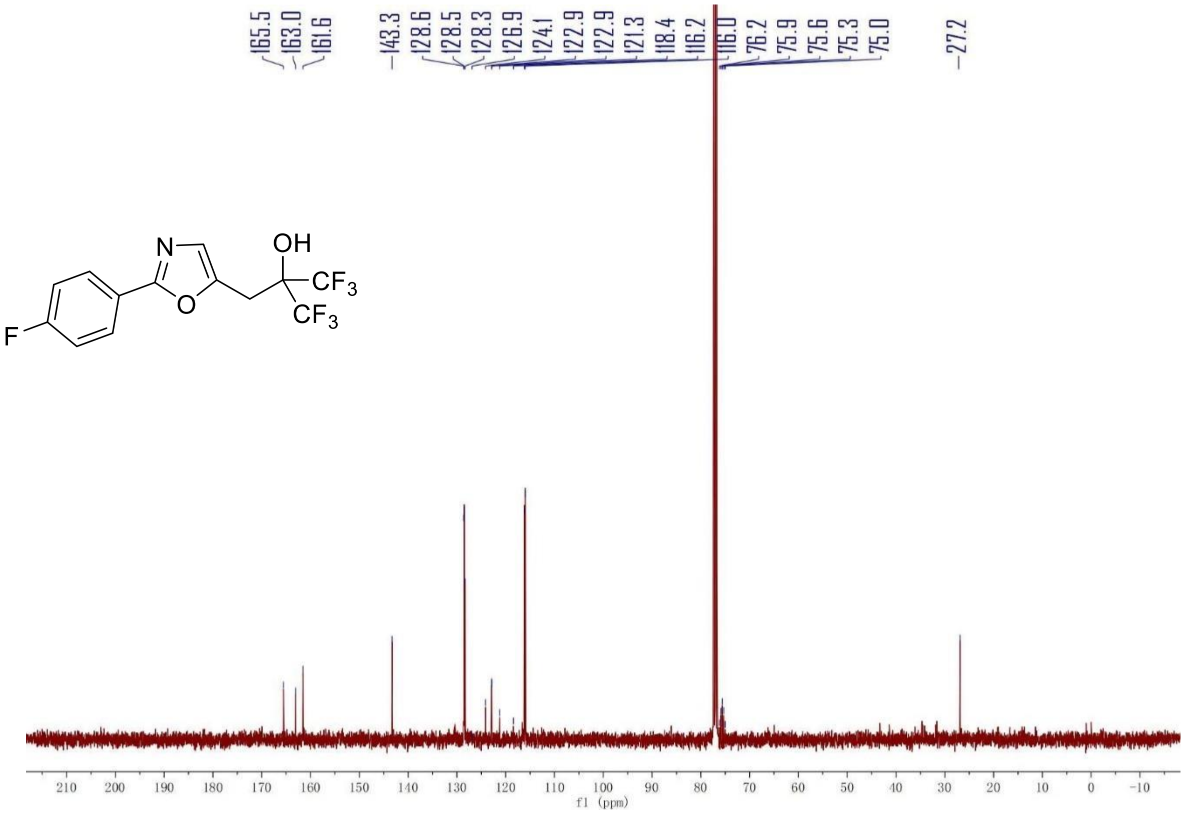

^{13}C NMR spectrum of compound **3ab**

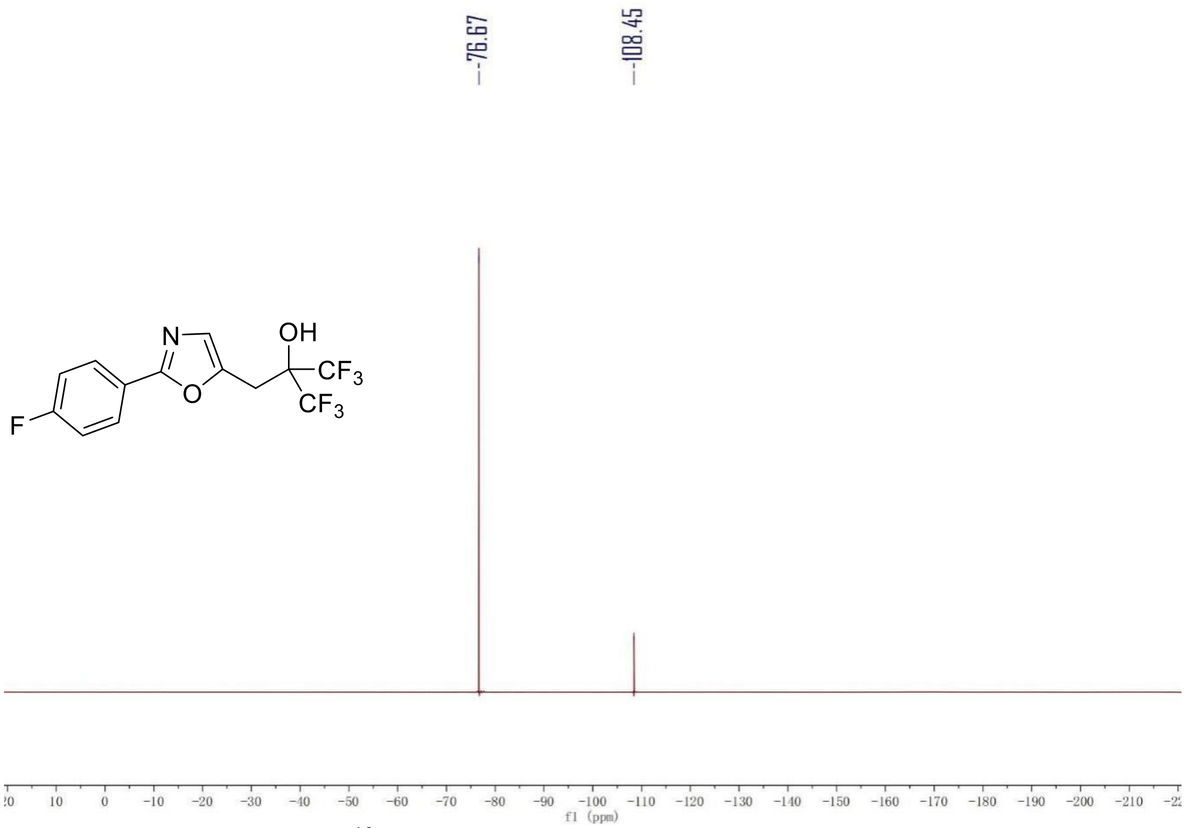

^{19}F NMR spectrum of compound **3ab**

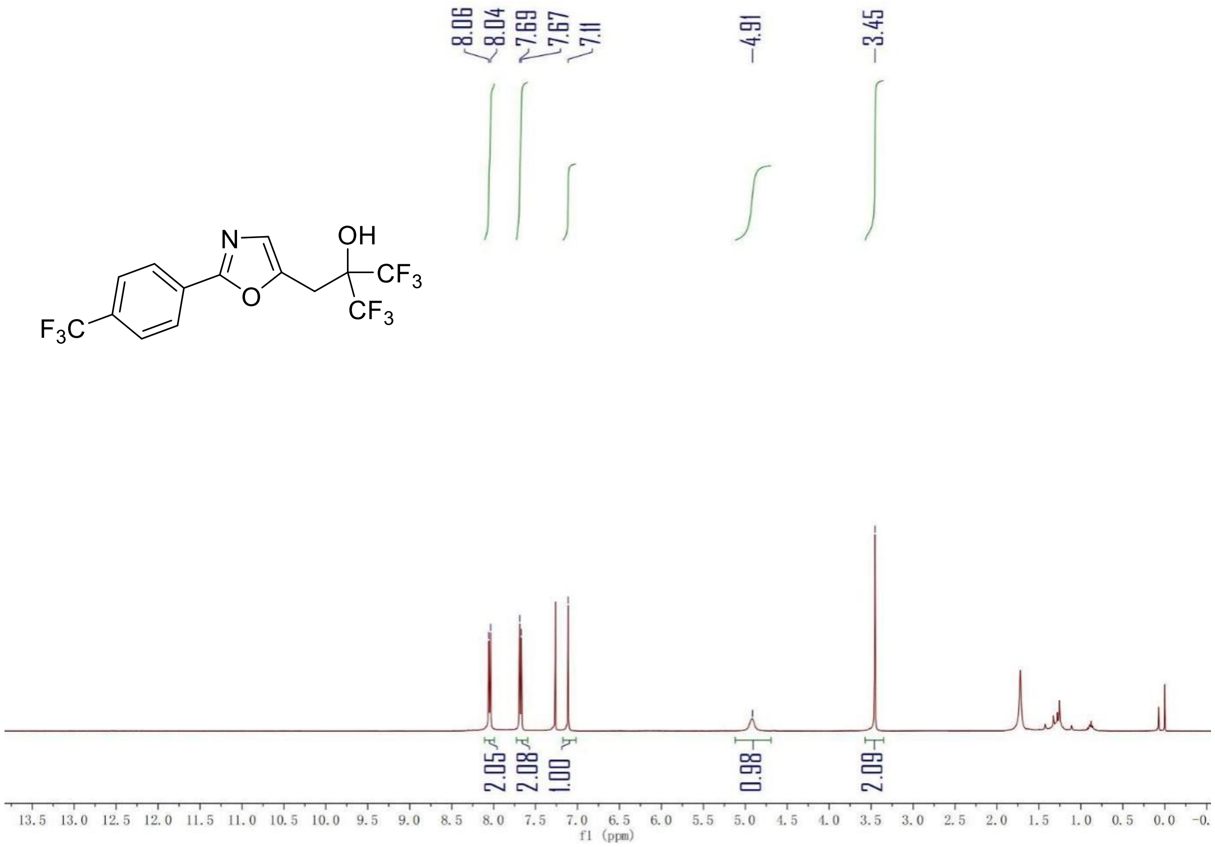

^{1}H NMR spectrum of compound **3ac**

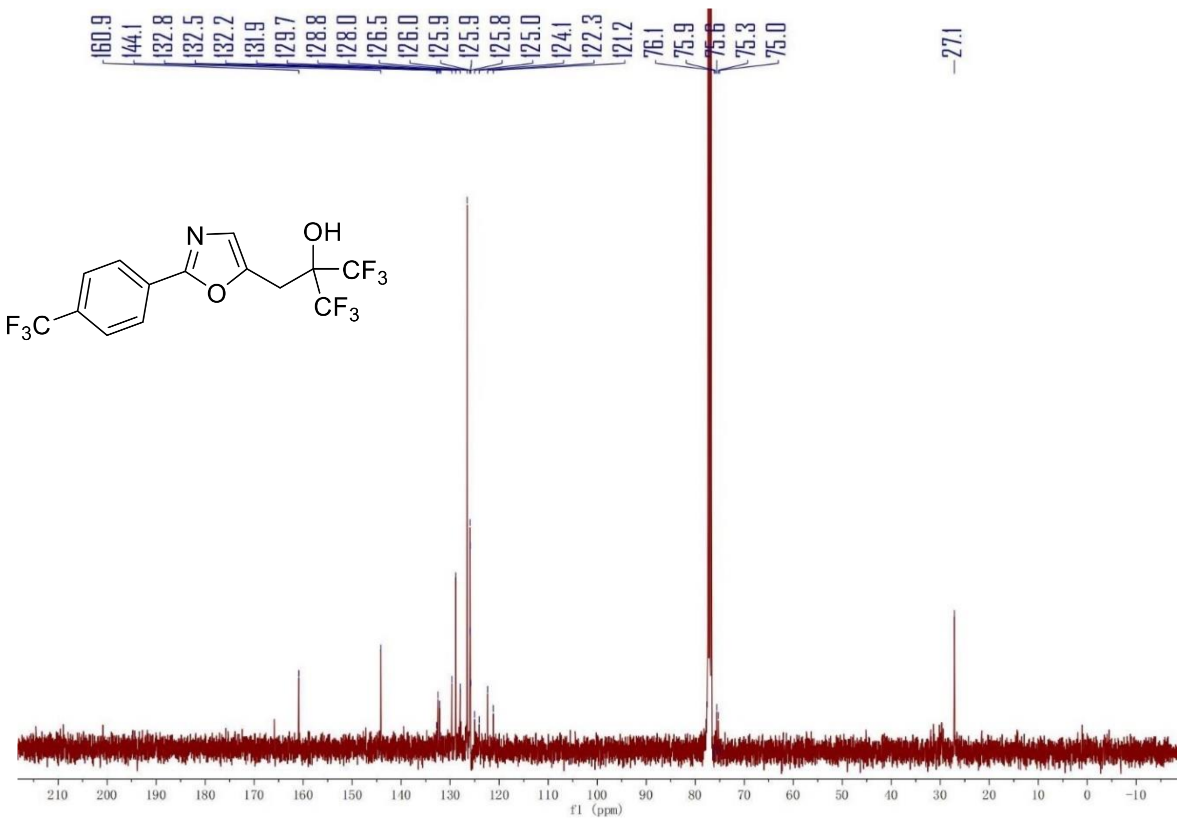

^{13}C NMR spectrum of compound **3ac**

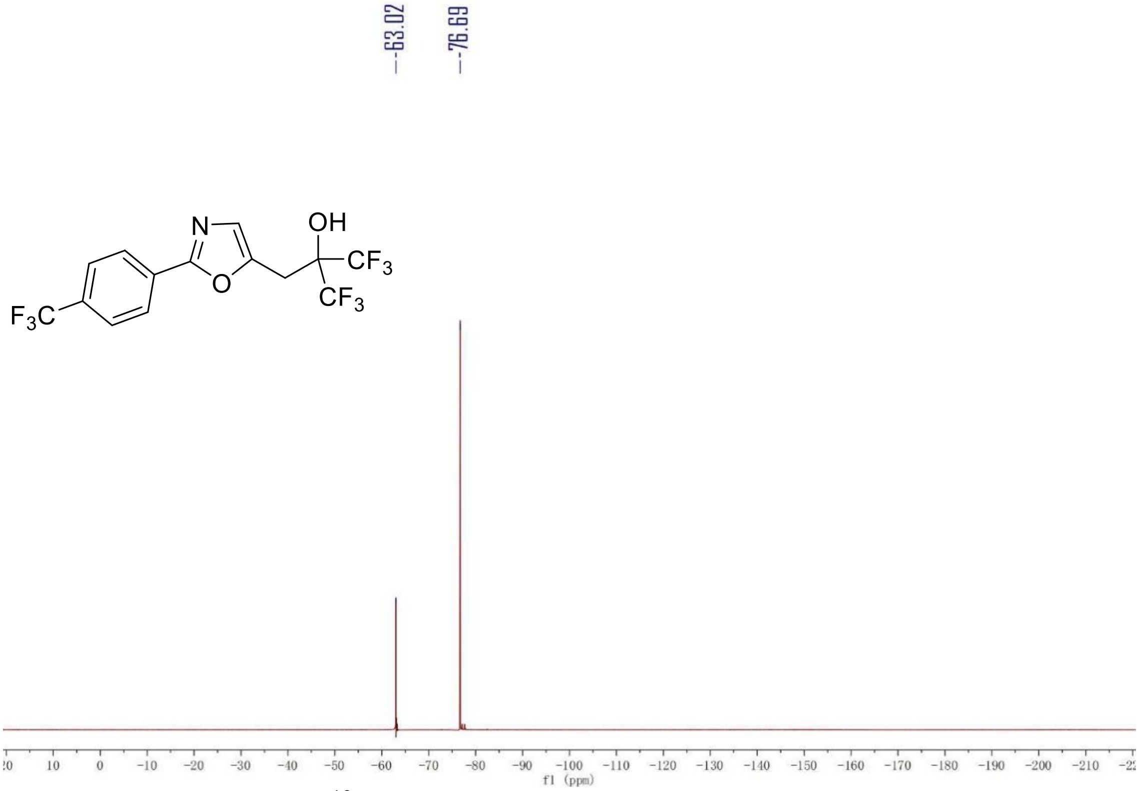

^{19}F NMR spectrum of compound **3ac**

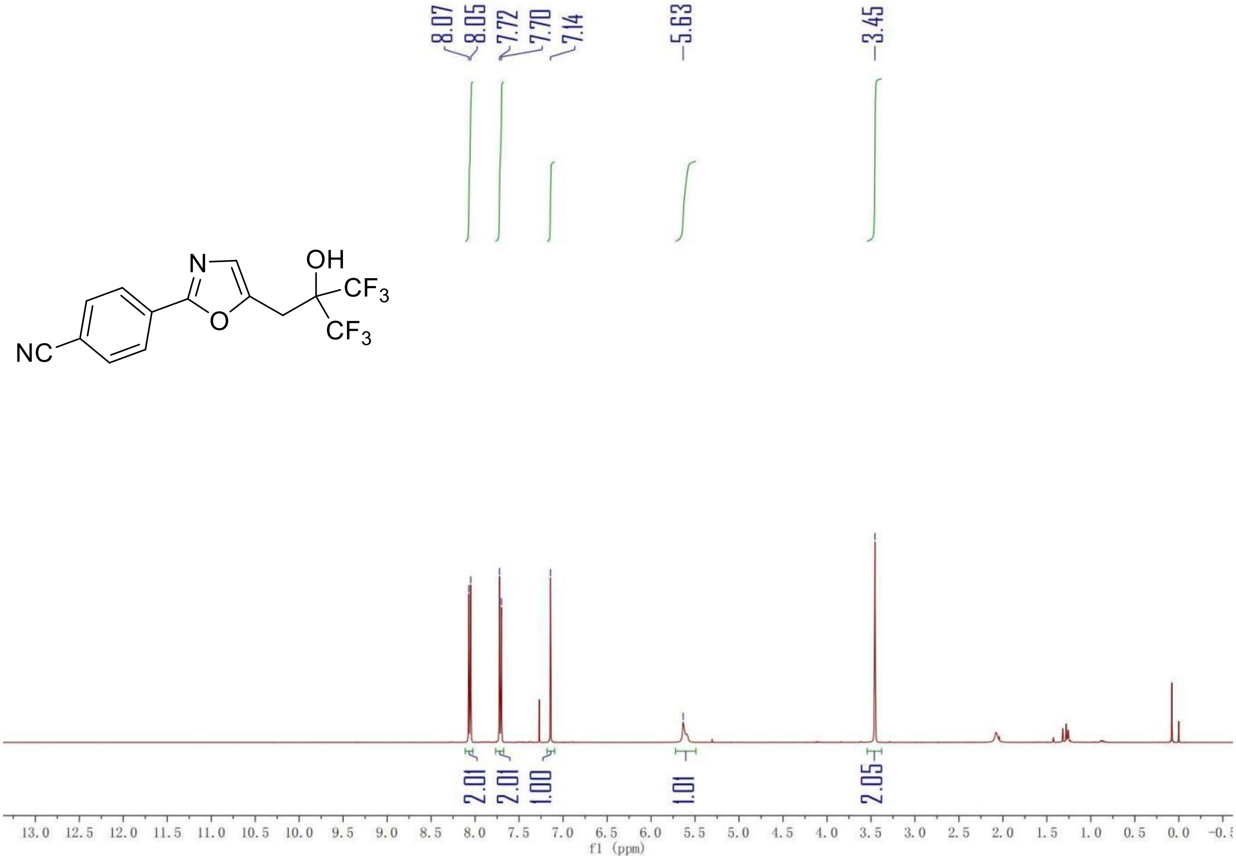

^{1}H NMR spectrum of compound **3ad**

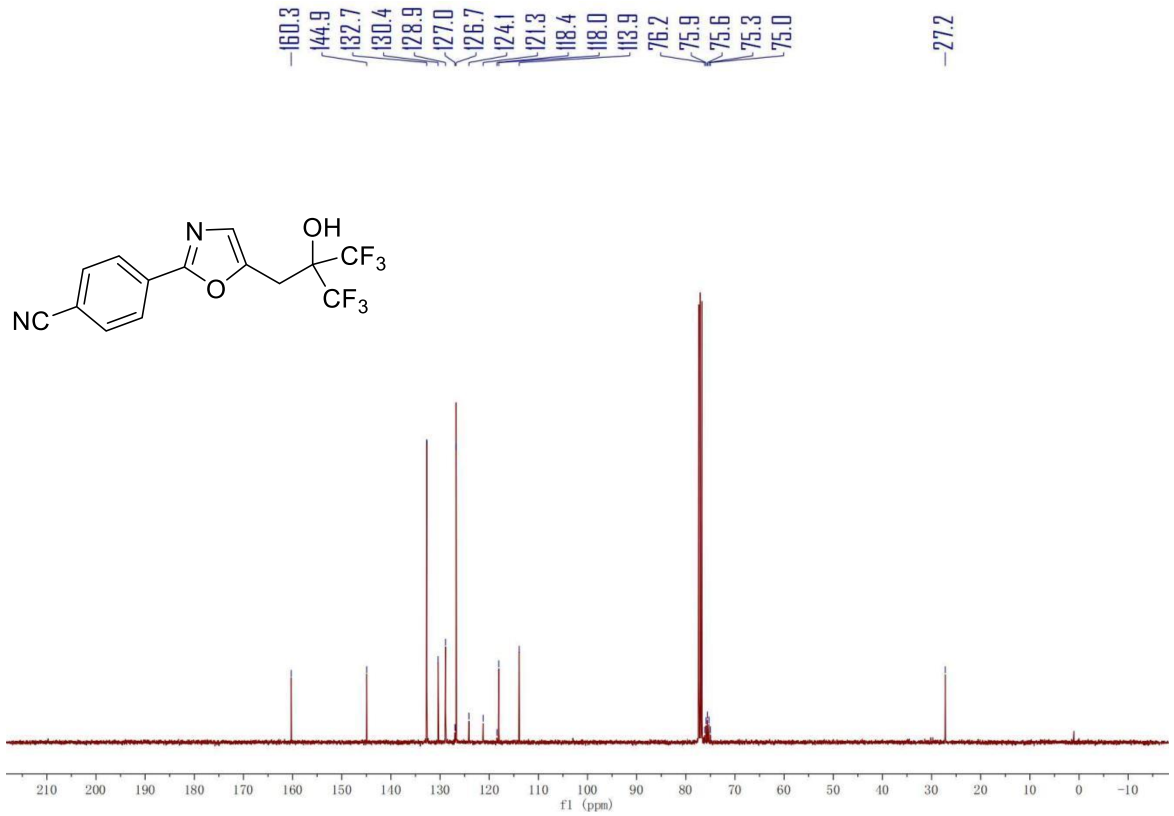

^{13}C NMR spectrum of compound **3ad**

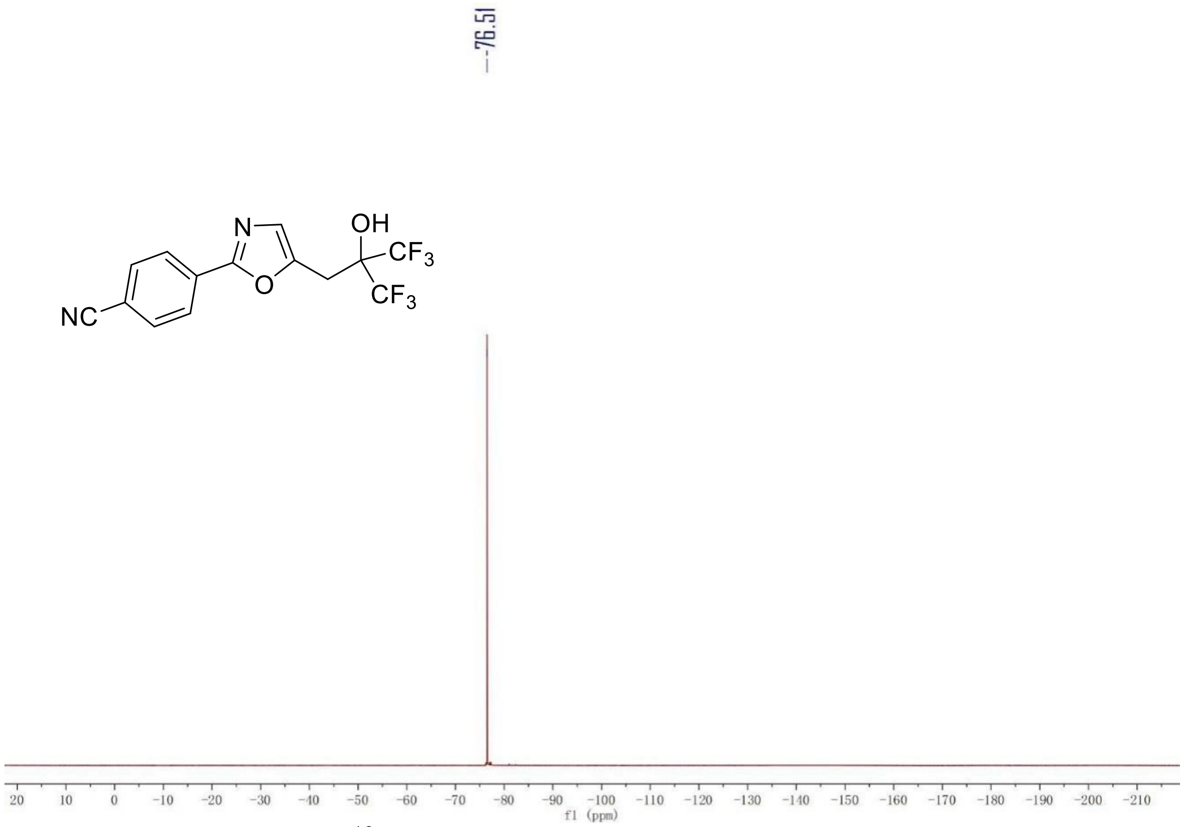

^{19}F NMR spectrum of compound **3ad**

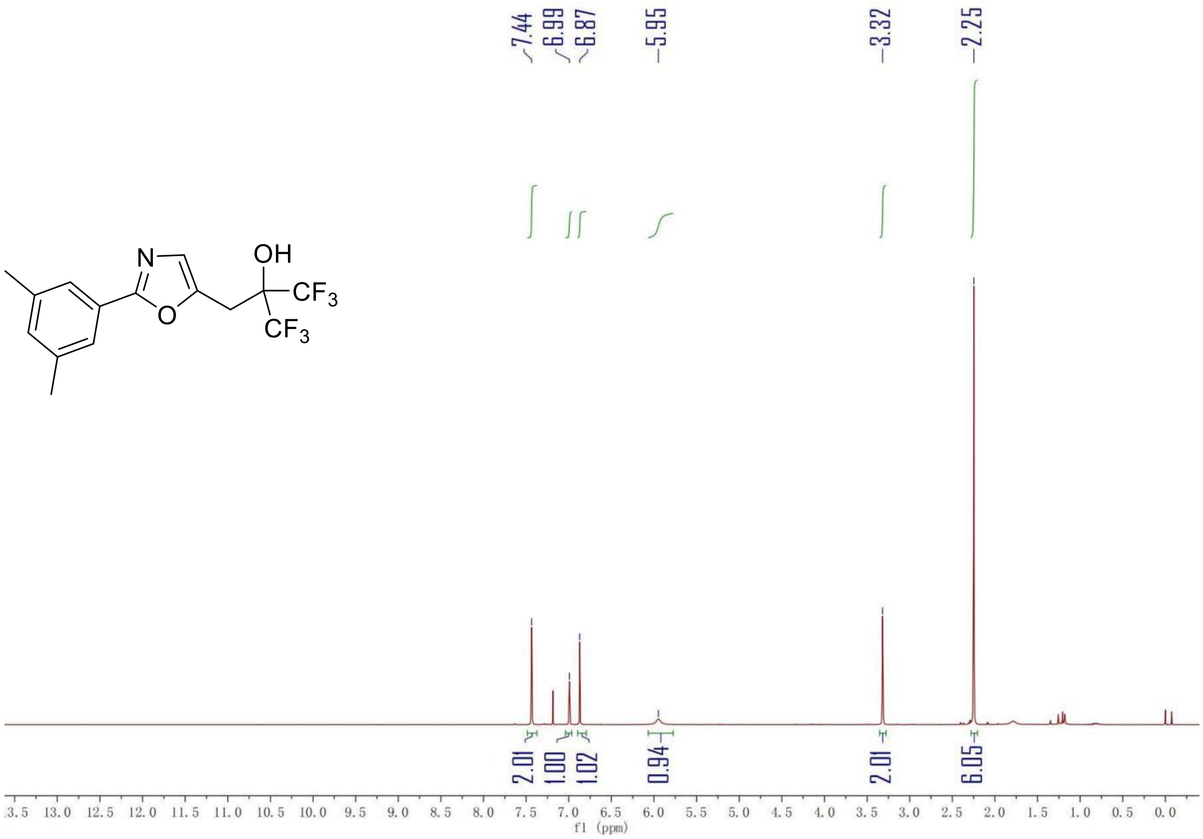

^{1}H NMR spectrum of compound **3ae**

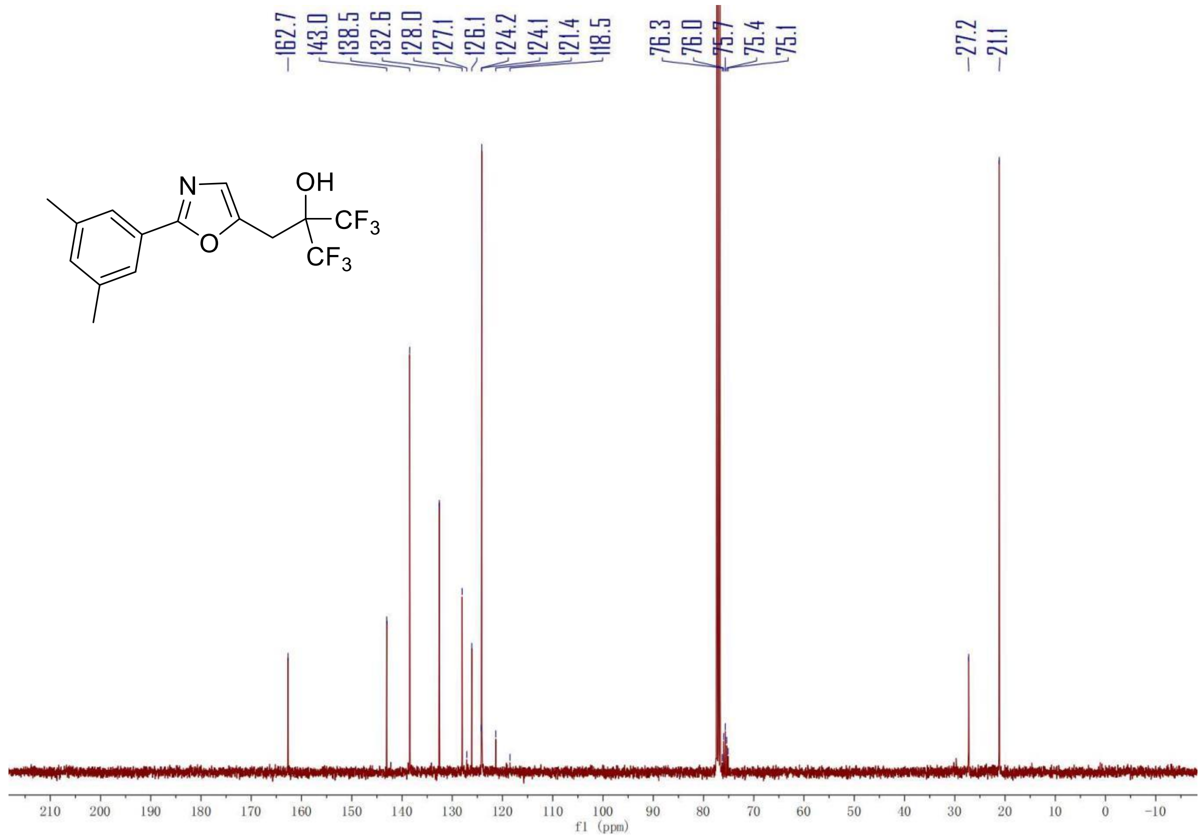

^{13}C NMR spectrum of compound **3ae**

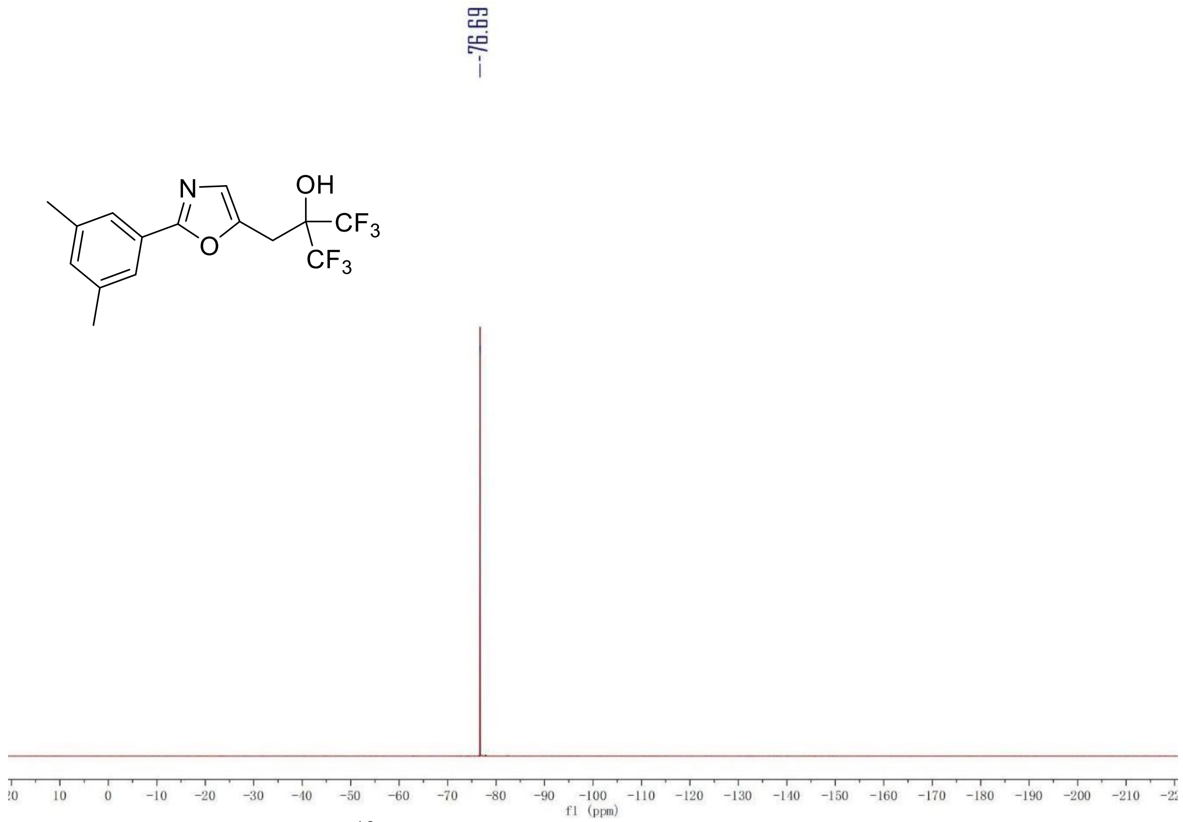

^{19}F NMR spectrum of compound **3ae**

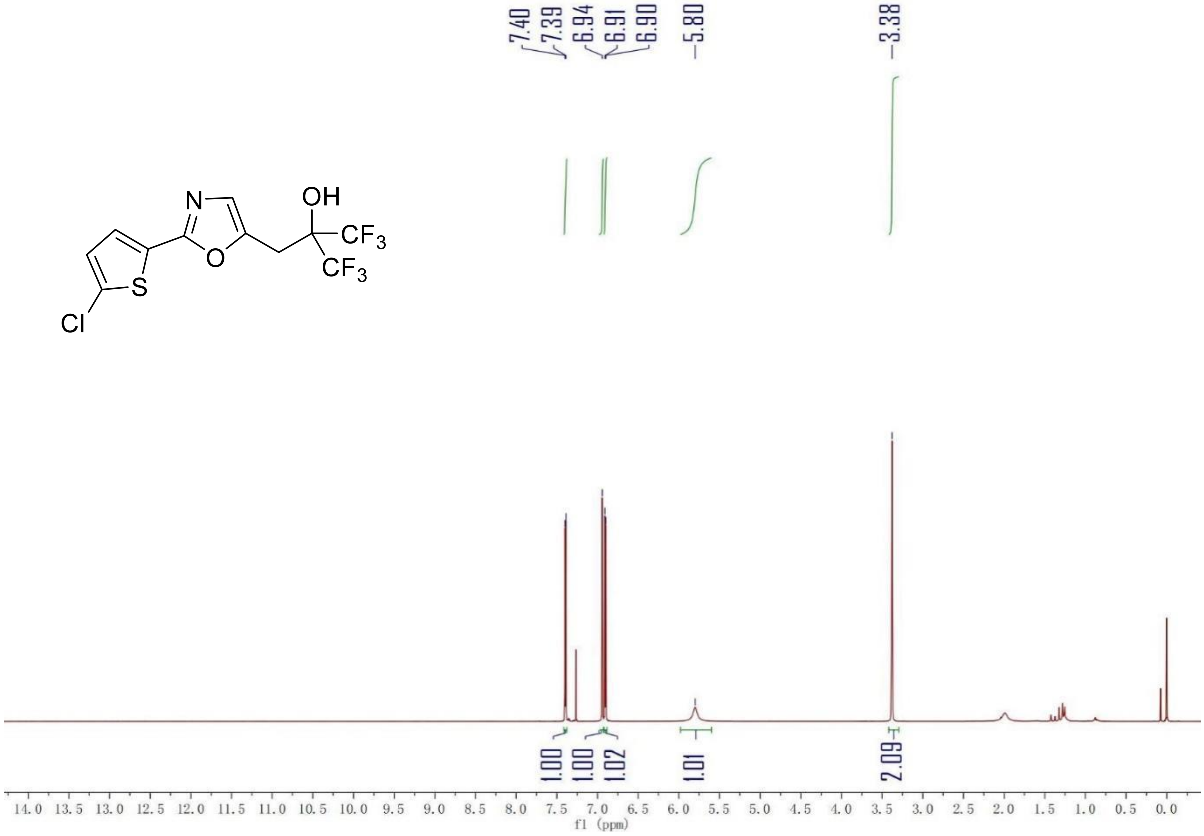

^{1}H NMR spectrum of compound **3af**

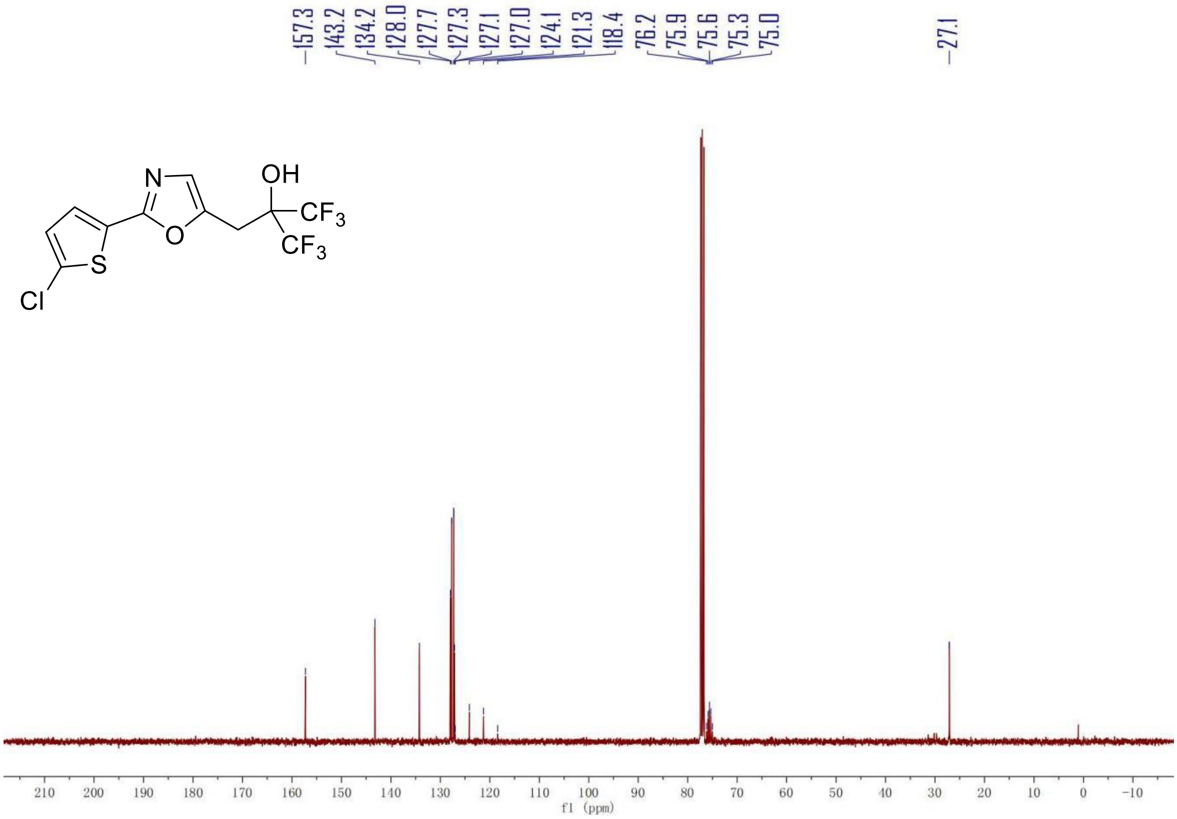

^{13}C NMR spectrum of compound **3af**

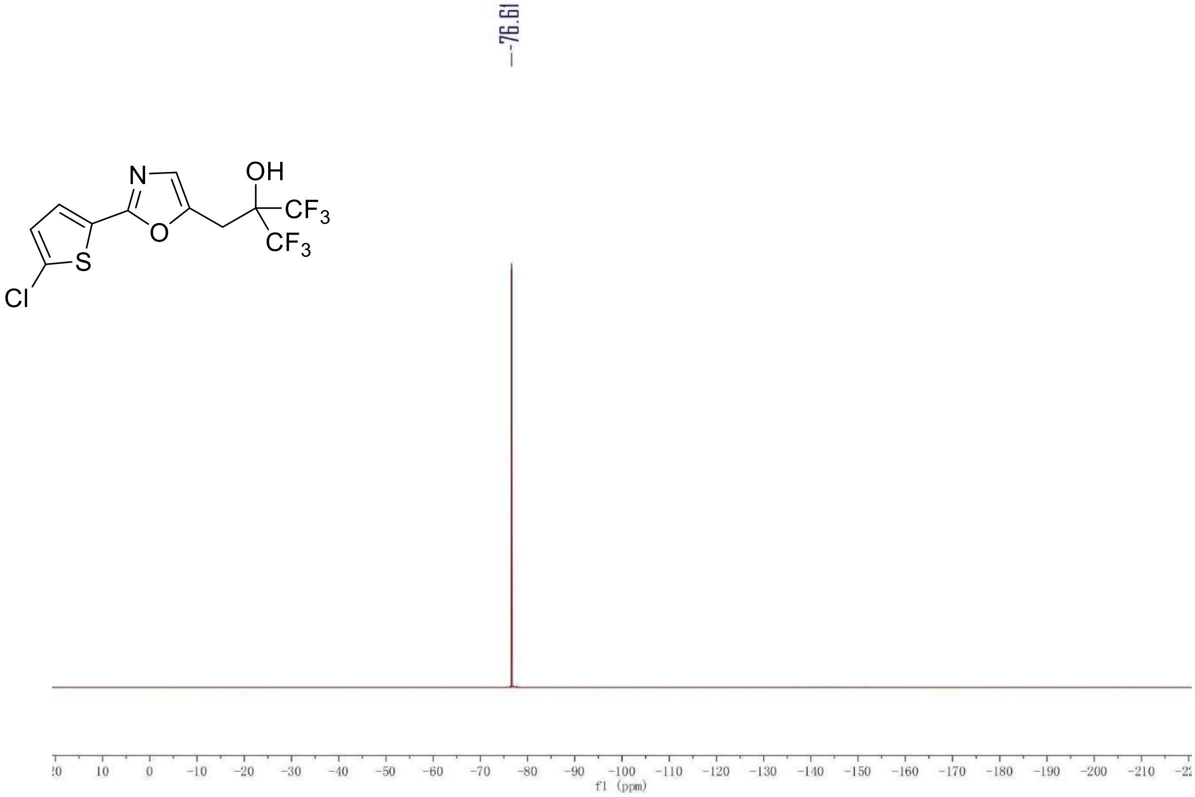

^{19}F NMR spectrum of compound **3af**

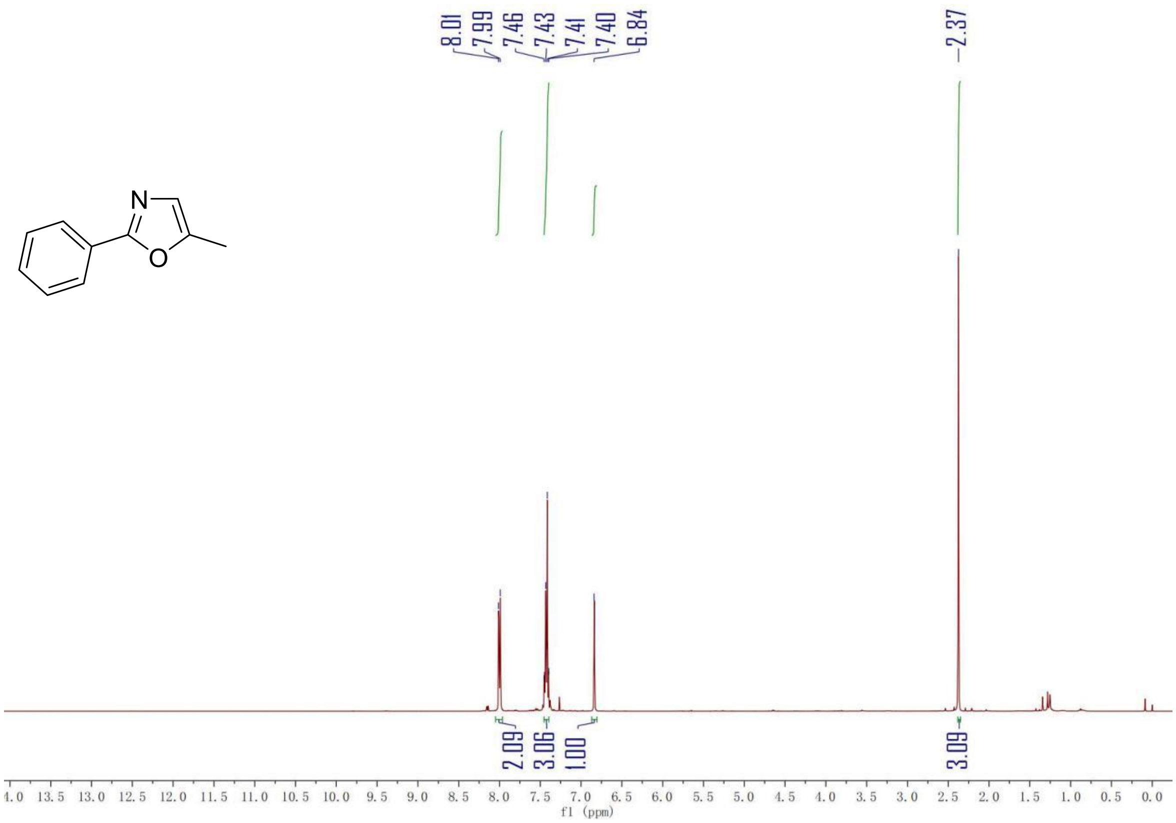

^{1}H NMR spectrum of compound **4a**

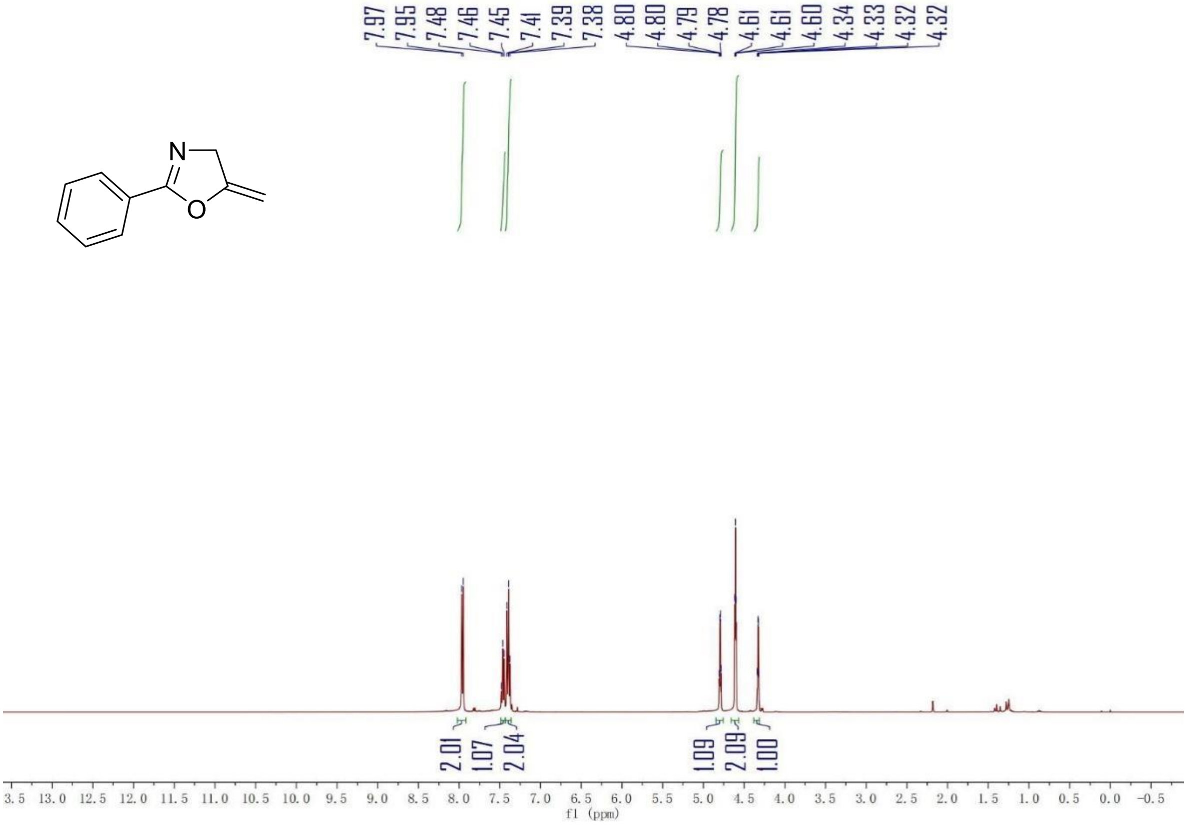

^{1}H NMR spectrum of compound **5a**
